# Regulatory mechanisms of maternal imprinting at the murine Dlk1-Dio3 domain

**DOI:** 10.1038/s41467-026-77129-4

**Published:** 2026-09-02

**Authors:** Ariella Weinberg-Shukron, Frances L. Dearden, Ana Moreno-Barriga, Lok Ting Nick New, Marco Müller, Rebecca C. Rancourt, Carol A. Edwards, Anne C. Ferguson-Smith

**Affiliations:** 1 Department of Genetics, University of Cambridge, Cambridge, UK; 2 Present Address: Medical Research Council (MRC) Laboratory of Medical Sciences (LMS), London, UK; 3 Present Address: BioNTech SE, Mainz, Germany; 4 Present Address: Institute of Clinical Research, Medical School Berlin (MSB), Berlin, Germany

**Keywords:** Imprinting, Gene expression, Dosage compensation, Transcriptional regulatory elements

## Abstract

Genomic imprinting is an epigenetic process causing parent-of-origin specific gene expression. The Dlk1-Dio3 domain is one of the largest imprinted clusters. While DNA methylation at an intergenic CpG-island (IG-CGI) within the imprinting control region (ICR) controls expression from the paternal chromosome, mechanisms regulating the unmethylated maternal chromosome remain unknown. Within the transcriptional regulatory element (IG-TRE) of the ICR, deletions identified a minimal region in vitro exhibiting both silencing and enhancing activity, with SOX2 and ZFP281 contributing to enhancer function on the maternal chromosome. In vivo, however, this deletion did not affect maternal expression in mouse embryos; instead it activated *Dlk1* on both parental chromosomes. Combining deletion of this IG-TRE with the lethal IG-CGI deletion rescued lethality in mice by balancing *Dlk1* expression, despite persistent maternal gene upregulation. These results demonstrate that loss of expression at this domain is more detrimental than gain, highlighting the importance of in vivo analysis. Identification of active regulatory factors on the unmethylated maternal chromosome challenges the prevailing view that imprinting is primarily a methylation-driven phenomenon, further revealing the sophisticated hierarchical mechanisms governing imprinting control.

## Abstract (web summary)

Researchers reveal that genomic imprinting is regulated by mechanisms beyond DNA methylation, identifying key transcription factors acting on the unmethylated maternal chromosome and providing insights into gene dosage control during development.

## Introduction

Genome function is regulated temporally and tissue-specifically through the orchestrated interplay of regulatory factors, genomic features, and epigenetic states. Epigenetic modifications are dynamic during development and across the cell cycle. A hierarchy of successive epigenetic states, including DNA methylation, ensures the creation of healthy individuals. In mammals, extensive epigenetic reprogramming events occur during germ cell development, fertilization, and early embryogenesis^1^.

One major process that maintains epigenetic memory in the germline is that of mammalian genomic imprinting^2,3^. Genomic imprinting is an epigenetically regulated process causing genes to be expressed according to the parent-of-origin. Imprinting is highly conserved in eutherian mammals, with mouse studies providing insights into the repertoire of developmental and physiological pathways regulated by imprinted genes^4,5^.

Differentially DNA methylated regions (DMRs) at imprinting control regions (ICRs) are established in the parental germlines^6^. They are protected from the global wave of demethylation in the early embryo by the KRAB zinc finger proteins ZFP57 and ZFP445 in order to maintain the epigenetic memory of parental origin^7^. Failure to correctly establish and maintain imprints is associated with developmental syndromes, including growth abnormalities, neurological and metabolic disorders, and numerous forms of cancer^8,9^. The extreme dosage sensitivity of imprinted genes highlights the complex and dynamic nature of their gene expression and importance in human biology.

Genomic imprinting is a tractable model for studying epigenetic regulation, as the two genetically identical parental genomes within the same nucleus express a different repertoire of genes according to their parental origin. However, the dynamic hierarchy of events initiated by the ICR, which leads to the long-range, domain-wide, temporal, and tissue-specific behavior of imprinted genes, is not fully understood. By employing a systemic set of mutants at a single imprinted domain, we can dissect the relationship between allelic expression, dosage, epigenetic control, and phenotypic outcomes.

The developmentally regulated Dlk1-Dio3 imprinted domain, is conserved between mouse and human, spans 1.2 Mb and is one of the largest imprinted clusters. Failure to correctly imprint genes in this cluster in mice causes prenatal lethality, and in humans leads to Temple or Kagami–Ogata Syndromes, both exhibiting neurological, developmental, and behavioral impairments^10,11^.

**Fig. 1:**
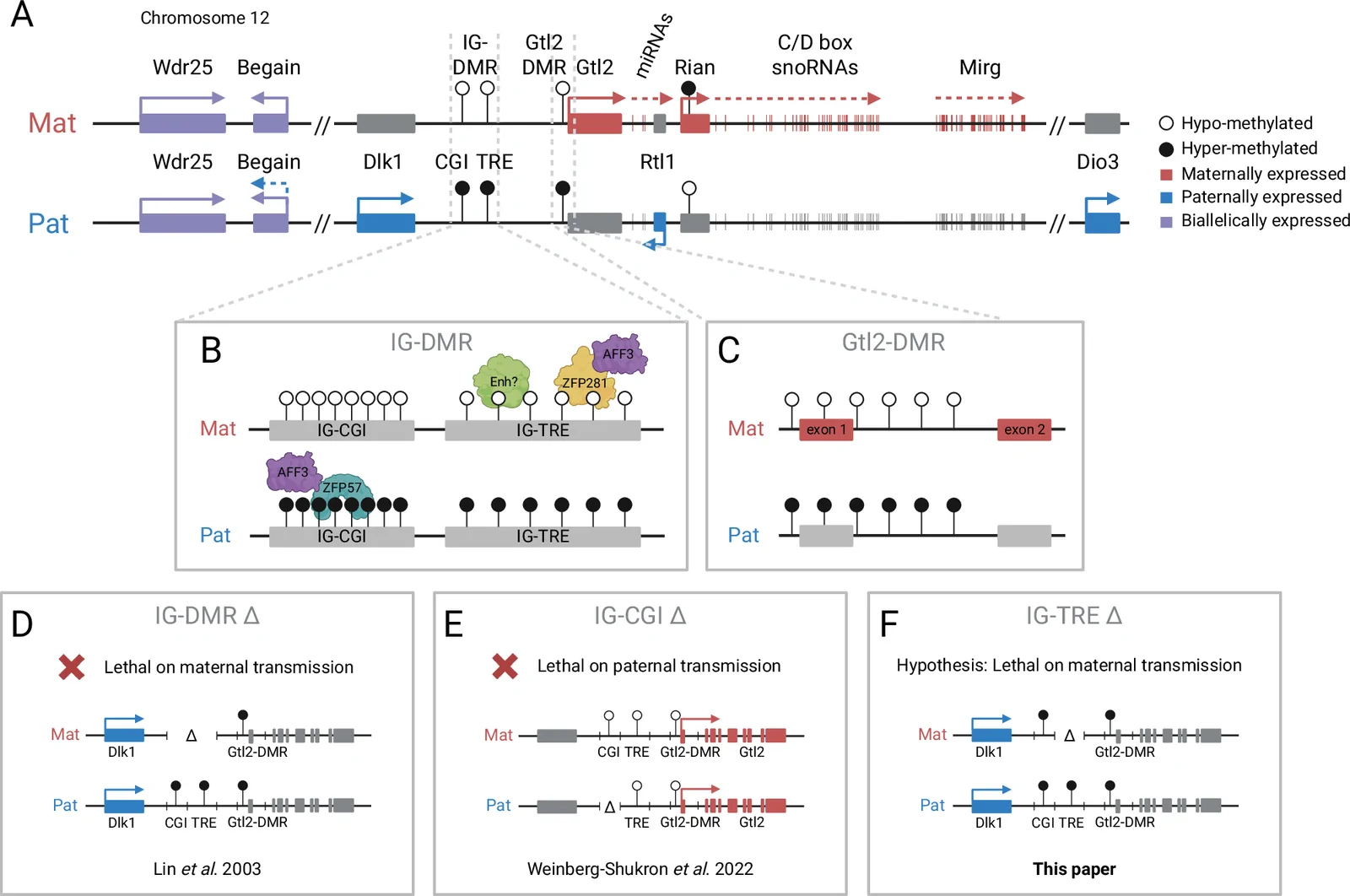
The Dlk1-Dio3 imprinted domain, key regulatory features, and deletion models. **A** The paternally inherited chromosome expresses *Dlk1*, *Rtl1*, *Dio3*, and one isoform of *Begain* (blue). The maternally inherited chromosome expresses *Gtl2*/*Meg3*, *antiRtl1*, and arrays of snoRNAs and miRNAs (red). The IG-DMR is methylated in sperm and unmethylated in oocytes and is the imprinting control region for the domain. DMRs are indicated by circles: black (methylated), white (unmethylated). **B** The IG-DMR contains a CpG island (IG-CGI) that binds ZFP57 (turquoise) on the methylated paternal copy and a transcriptional regulatory element (IG-TRE) that has an enhancer-like function on the unmethylated maternal copy (green). The IG-DMR also binds AFF3 (purple) through ZFP57 at the IG-CGI on the methylated paternal chromosome and co-bound with ZFP281 (yellow) at the IG-TRE on the unmethylated maternal chromosome. **C** The secondary somatic Gtl2-DMR is methylated in *cis* according to the methylation pattern of the IG-DMR. **D** Maternal deletion of the entire IG-DMR results in a maternal-to-paternal epigenotype switch^20^. **E** Paternal deletion of the IG-CGI results in a paternal-to-maternal epigenotype switch^30^. **F** Maternal deletion of the IG-TRE is hypothesized to result in a maternal-to-paternal epigenotype switch, similar to deletion of the entire IG-DMR. Created in BioRender. Edwards, C. (2026) https://BioRender.com/g67o4d9.

Four protein-coding genes: *Dlk1*, *Rtl1*, *Dio3*, and one isoform of *Begain* are preferentially expressed from the paternally inherited chromosome, and the maternally inherited chromosome expresses multiple imprinted noncoding transcripts, including *Gtl2* (also known as *Meg3*), *Rian*, and *Mirg* (Fig. 1A)^12^. *Gtl2* also forms a long polycistronic transcript along with its associated transcripts *Rian* and *Mirg*, acting as a host for multiple snoRNAs and miRNAs, including the miR-379/miR-410 cluster, all of which are driven by the *Gtl2* promoter^13–15^. Appropriate expression of genes in this region is essential for the lifelong health of mammals and understanding their epigenetic regulation has significant biomedical relevance for a diverse range of processes^16–19^.

Imprinting at the Dlk1-Dio3 domain is regulated by the germline-derived intergenic DMR (IG-DMR)^20^, which spans approximately 5 kb between *Dlk1* and *Gtl2* and is required for regulating parent-specific expression across the locus^21^. The IG-DMR is normally methylated in sperm and unmethylated in oocytes and maintains this parent-specific pattern throughout development (Fig. 1A, B). After implantation, a secondary somatic DMR is established at the promoter of the *Gtl2* gene (the Gtl2-DMR), which is also hypermethylated on the paternal chromosome^22^ (Fig. 1C).

The IG-DMR was recently established as a bipartite element comprised of two distinct functional elements^23^ (Fig. 1B). The first, termed the IG-CGI, is a small CpG island comprising seven tandemly repeated sequences of which five contain a ZFP57 binding motif^24^. The sequence-specific zinc finger protein, ZFP57, only binds to the methylated paternal chromosome, where it interacts with TRIM28, which recruits the repressive epigenetic machinery, including DNA methyltransferases (DNMTs) and heterochromatin-associated proteins, thereby maintaining the methylation memory of the germline imprint in early development at the time when most epigenetic modifications are being erased^25,26^. The IG-CGI has also been shown to bind AFF3, a component of the super elongation complex-like 3 (SEC-L3), on the methylated allele in mouse embryonic stem cells (mESCs) where it is thought to interact with the ZFP57/TRIM28 complex^27^ (Fig. 1B).

Maternal deletion of the IG-DMR leads to paternalization of the maternal chromosome and mice die between E16.5 and birth^20,28^ (Fig. 1D). Surprisingly, in contrast to the full IG-DMR deletion, paternal deletion of only the IG-CGI results in the reciprocal paternal-to-maternal epigenotype switch with the Gtl2-DMR becoming hypomethylated on the paternal chromosome^29,30^ (Fig. 1E). These data indicate that the IG-CGI is required to maintain a repressive chromatin landscape on the paternal chromosome, while the same element is dispensable on the maternal copy^30–32^. The experiments therefore indicate that the maternal epigenotype must be regulated by elements within the IG-DMR, independent of the CpG island. Together, the paradoxical effects imposed by distinct deletions within the IG-DMR represent an attractive experimental framework for dissecting the impact of changes in gene dosage on embryonic phenotypes.

The remaining part of the IG-DMR, located downstream of the IG-CGI, has the potential to be an enhancer. It binds pluripotency transcription factors in mESCs, exhibiting active enhancer marks (H3K27ac) and nascent transcription^27,33^. It was therefore termed the transcriptional regulatory element (IG-TRE) and suggested to drive expression of the maternally inherited genes within the domain (Fig. 1B)^23^.

The transcription factor AFF3 also binds to the 3′ side of the IG-DMR at the IG-TRE. There, AFF3 is co-bound with the zinc finger protein 281 (ZFP281/ZNF281), a zinc finger protein that has previously been reported to act as both a transcriptional activator and a repressor^34^ (Fig. 1B). In the Dlk1-Dio3 locus, depletion of ZFP281 from mESCs leads to decreased AFF3 binding at this downstream region. Since depletion of AFF3 leads to decreased expression of *Gtl2*, *Mirg*, and *Rian*, this suggests that the downstream bound region is relevant for AFF3 function and that it also acts as an enhancer for maternally expressed genes^35^. Whether this region is acting in concert with the IG-TRE to control expression of *Gtl2* and its associated transcripts remains to be established.

Recently, Hara et al. demonstrated that deletion of a 2.7 kb segment containing the IG-TRE results in postnatal lethality when maternally inherited^36^. However, smaller deletions of the IG-TRE subregions, including the AFF3/ZFP281-binding site alone, did not produce any obvious phenotype, suggesting the involvement of multiple elements spanning the IG-TRE in the regulation of the maternal epigenotype.

There is evidence suggesting a role for pluripotency-associated transcription factors such as OCT4, SOX2, and KLF4 in maintaining the unmethylated state of the maternal allele at imprints, potentially by forming a regulatory complex that promotes enhancer activity in pluripotent cells. For example, at the Igf2-H19 imprinted locus, SOX2 and OCT4 bind to the H19-ICR, and preserve the hypomethylated status of the maternal allele^37,38^. Whether this is a feature of these factors at other imprinted loci such as the IG-DMR is useful to ascertain. However, while the multiple transcription factor binding motifs suggest that the IG-TRE is most likely a positive regulator for *Gtl2*, it is possible that the IG-TRE also contains a silencer element that is capable of directly repressing the paternally expressed genes on the maternally inherited chromosome. Experiments dissecting this region further are necessary to tease apart these options (Fig. 1F).

Here, we defined the minimal region of activity at the IG-TRE in vitro, generated an in vivo knockout mouse model to dissect the function of the IG-TRE during mouse development, and pieced these together with the previous deletion models to understand how the parental-origin-specific epigenetic landscapes, genetic elements, and transcription are exquisitely coordinated to regulate genome function at this domain.

## Results

### Functional dissection of the IG-DMR in vitro

The IG-DMR knockout in mice demonstrated that it regulates the activity and repression of multiple imprinted genes in *cis* and influences differential methylation at the Gtl2-DMR in the Dlk1-Dio3 domain^20^ but, though studies have shown it contains two features (IG-CGI and IG-TRE) acting individually on the paternally and maternally inherited imprinted gene cluster respectively, a systematic dissection of the regulatory features within the IG-DMR has not been conducted, making it unclear how it works. In order to address this, we used an in vitro luciferase reporter gene assay in a manner described for other imprinted clusters^39,40^.

**Fig. 2:**
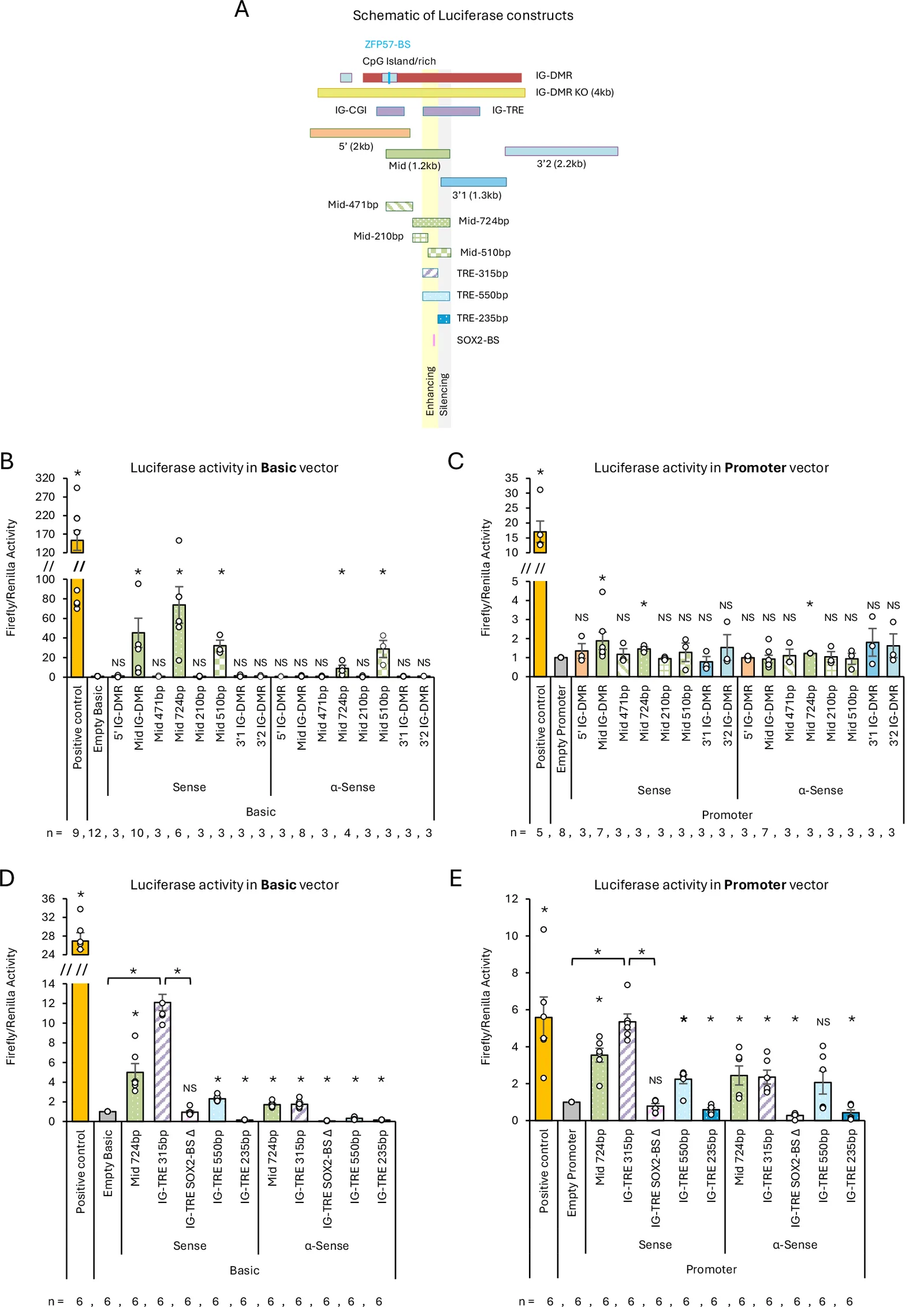
Dual activity at a minimal 550 bp region at the IG-TRE: The 315 bp region, and SOX2 binding within it, has an enhancer-like function, while the downstream 235 bp region is silencing. **A** Schematic of the IG-DMR fragments tested for activity by a luciferase reporter assay. Each fragment was cloned in both sense and antisense orientation into pGL3 vectors upstream of the firefly luciferase reporter gene. **B** Graphical representation of the luciferase activity in the pGL3 Basic vector. For positive control, empty basic, 5′ IG-DMR, Mid IG-DMR, Mid 471 bp, Mid 742 bp, Mid 210 bp, Mid 510 bp, 3′1 IG-DMR and 3′2 IG-DMR in sense and α-sense, *n* = 9, 12, 3, 10, 3, 6, 3, 3, 3, 3, 3, 8, 3, 4, 3, 3, 3 and 3, respectively, and significant *P* values are: 2.31E^−06^, 0.004, 3.2E^−05^, 1.1E^−08^, 0.0003, 6.4E^−06^. **C** Graphical representation of the luciferase activity in the pGL3 Promoter vector. For positive control, empty basic, 5′ IG-DMR, Mid IG-DMR, Mid 471 bp, Mid 742 bp, Mid 210 bp, Mid 510 bp, 3′1 IG-DMR and 3′2 IG-DMR in sense and α-sense, *n* = 5, 8, 3, 7, 3, 3, 3, 3, 3, 3, 3, 7, 3, 3, 3, 3, 3 and 3 respectively, and significant *P* values are: 0.0001, 0.05, 2.9E^−05^, 8.9E^−17^. The Mid 724 bp fragment shows activity in basic and promoter vectors in sense orientation only, suggesting promoter activity. **D** Graphical representation of the luciferase activity in the pGL3 Basic vector. For positive control, empty basic, Mid 742 bp, IG-TRE 315 bp, IG-TRE SOX2-BS ∆, IG-TRE 550 bp, and IG-TRE 235 bp in sense and α-sense, *n* = 6, 6, 6, 6, 6, 6, 6, 6, 6, 6, 6, and 6, respectively, and significant *P* values are: 5.4E^−08^, 0.001, 1.1E^−07^, 1.1E^−07^, 1.3E^−06^, 6.2E^−16^, 1.2E^−04^, 0.002, 3E^−15^,7.6E^−07^, 1.03E^−16^. **E** Graphical representation of the luciferase activity in the pGL3 Promoter vector. For positive control, empty promoter, Mid 742 bp, IG-TRE 315 bp, IG-TRE SOX2-BS ∆, IG-TRE 550 bp, and IG-TRE 235 bp in sense and α-sense, *n* = 6, 6, 6, 6, 6, 6, 6, 6, 6, 6, 6, and 6, respectively, and significant *P* values are: 0.002, 5.4E^−05^, 1.4E^−06^, 1.5E^−06^, 0.0004, 0.003, 0.01, 0.005, 1.5E^−07^, 0.003. The Mid 315 bp fragment shows activity in basic and promoter vectors, similar to the 724 bp fragment, suggesting enhancer activity. This activity is lost upon deletion of the SOX2-binding site from within the 315 bp fragment. In parallel, the 235 bp fragment, downstream to the 315 bp region, shows silencing activity, indicating dual activity in a minimal region of 550 bp in the IG-TRE with both enhancing and silencing regulation. Each construct was measured twice in triplicate wells. The firefly luciferase values were normalized to the *Renilla* values, and then each test construct was normalized to the corresponding empty vector. Data are presented as mean values +/− SEM. NS- not significant. Asterisks indicate statistical significance in comparison to the empty vector using a two-tailed unpaired Student’s *t* test.

A 6.125 kb region of the IG-DMR was divided into four overlapping fragments (Fig. 2A). The first 2 kb of the IG-DMR knockout region is referred to as the 5′ fragment, which encompasses the IG-CGI and contains tandem repeat arrays identified by Paulsen et al.^41^ The next fragment, referred to as Mid, overlaps the 5′ fragment and is located in the core of the differentially methylated region. It is 1.2 kb and also contains the CpG island and the tandem repeat array. At the end of the original knockout region^18^ is the 3′1 fragment, which is 1.3 kb and located within the differentially methylated region. Finally, the 3′2 fragment spanning 2.2 kb includes the 3′ sequence downstream of the IG-DMR knockout region. All fragments were cloned in both orientations, with the sense (S) referring to the direction towards *Gtl2* and antisense (AS) referring to the direction towards *Dlk1*.

The 1.2 kb Mid fragment (mid IG-DMR in Fig. 2) revealed promoter-like and some enhancer activity in mESCs only in the sense orientation compared to the empty vectors (Figs. 2B,C and  S1A, *P*_Basic_= 0.004; *P*_Promoter_= 0.05; *P*_Enhancer_ = 0.04). Activity was not detected from any of the antisense constructs.

In order to narrow down the positive regulatory region, the Mid fragment was divided into smaller sections. The original 1.2 kb Mid fragment was first separated into two fragments: 471 bp (Mid-471) and 724 bp (Mid-724), and assayed in ES cells. The results showed that the promoter-like activity was present in the Mid-724 (sense orientation) while the Mid-471 fragment had no activity (Figs. 2B,C and  S1A, *P*_Basic_=3.2E^−05^, 0.0003; *P*_Promoter_=2.9E^−05^, 8.9E^−17^; *P*_Enhancer_=8.8E^−09^,9.04E^−06^). Next, to further test the minimal region of activity, the 724 bp Mid region was divided into two additional fragments: 210 bp (Mid-210) and 510 bp (Mid-510). No activity was observed from Mid-210, while promoter-like activity was seen in Mid-510. However, Mid-510 had a lower level of activity than that of Mid-724 (Figs. 2B, C and  S1A, *P*_Basic_=1.1E^−08^,6.4E^−06^). These results reveal that the 724 bp fragment is the minimal region for effective positive regulation, which the smaller fragments alone could not achieve.

### Deleting the IG-TRE in vitro results in dysregulation of the maternal epigenotype

**Fig. 3:**
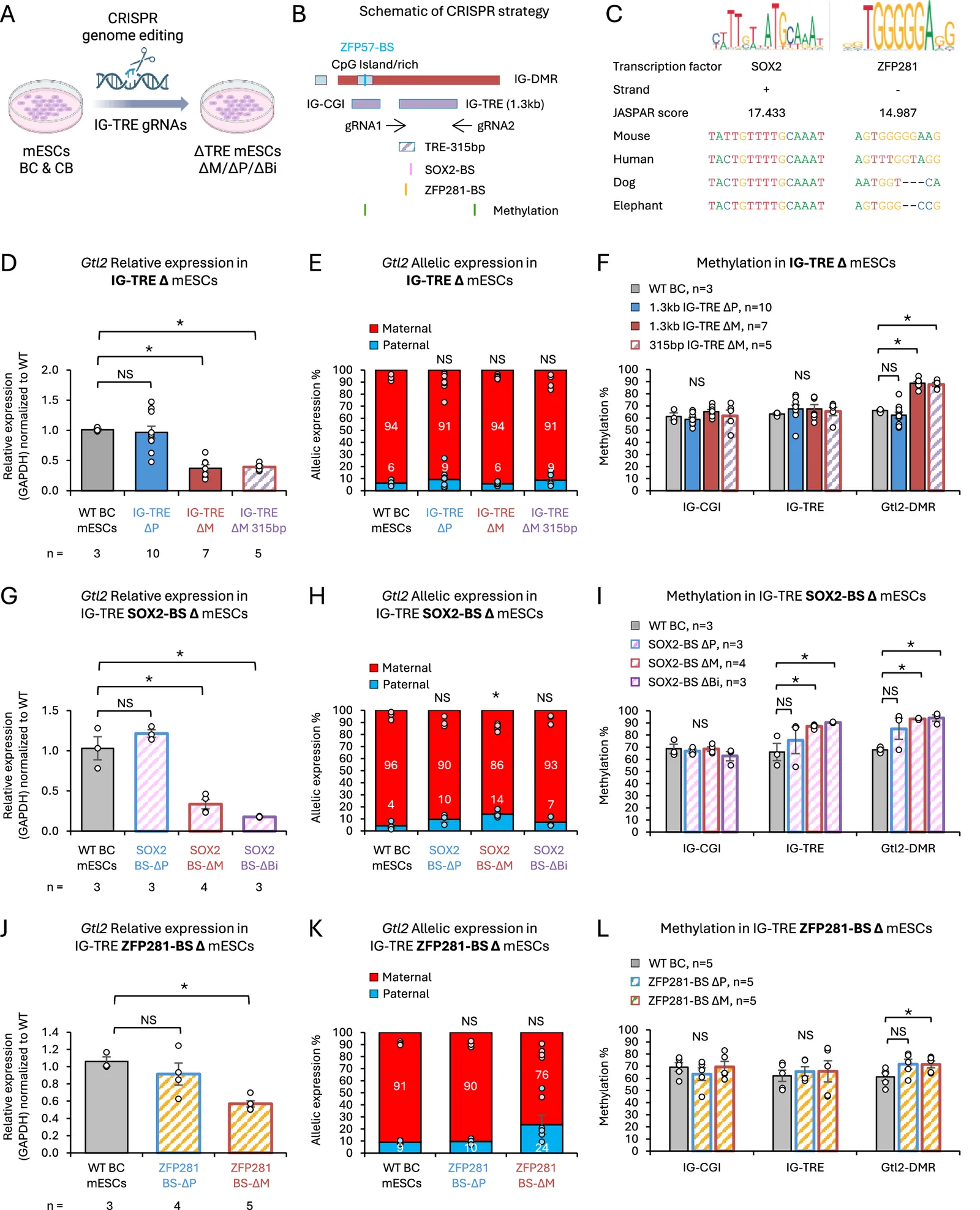
Deleting the IG-TRE in vitro results in dysregulation of the maternal epigenotype. **A** Description of the CRISPR genome editing strategy in mESCs. Created in BioRender. Edwards, C. (2026) https://BioRender.com/69rewbe. **B** Schematic of the gRNA locations, IG-TRE deletions, SOX2 and ZFP281-binding sites, and methylation analysis regions. **C** Motifs and conservation of the SOX2 and ZFP281 transcription factor binding sites within the IG-TRE, showing they are well conserved in placental mammals. **D** Graphical representation of *Gtl2* relative expression in mESC clones deleted for the entire IG-TRE (1.3 kb) showing major reduction in *Gtl2* expression upon full maternal or 315 bp maternal deletions (*P*_∆M_ = 0.0002, *P*_∆M-315bp_ = 4.1E^−06^). **E** Graphical representation of *Gtl2* allelic expression in mESC clones deleted for the entire IG-TRE (1.3 kb) or for 315 bp from the maternal chromosome. **F** Graphical representation of the methylation at three regulatory elements at the Dlk1-Dio3 domain, analyzed by bisulfite pyrosequencing, shows increased methylation at the Gtl2-DMR upon deletion of the entire 1.3 kb IG-TRE and even just 315 bp from the maternal chromosome (*P*_∆M_ = 1.3E^−05^, *P*_∆M-315bp_ = 8.9E^−05^). *n*_WTBC_ = 3, *n*_IG-TRE-∆P_ = 10, *n*_IG-TRE-∆M_ = 7, *n*_IG-TRE-∆M-315bp_ = 5 independent biological replicates. **G** Graphical representation of *Gtl2* relative expression in mESC clones deleted for the SOX2-binding site within the 315 bp of the IG-TRE, showing major reduction in *Gtl2* expression upon maternal or biallelic deletions (*P*_∆M_ = 0.004, *P*_∆Bi_ = 0.004). **H** Graphical representation of *Gtl2* allelic expression in mESC clones deleted for the SOX2-binding site within the 315 bp of the IG-TRE (*P*_∆M_ = 0.01). **I** Graphical representation of the methylation at three regulatory elements at the Dlk1-Dio3 domain, analyzed by bisulfite pyrosequencing, shows increased methylation at the IG-TRE and Gtl2-DMR upon deletion of the SOX2-binding site from within the 315 bp of the IG-TRE from the maternal but not paternal chromosome (IG-TRE *P*_∆M_ = 0.02, *P*_∆Bi_ = 0.03; Gtl2-DMR *P*_∆M_ = 8.7E^−06^, *P*_∆Bi_ = 0.001). *n*_WTBC_ = 3, *n*_SOX2-BS-∆P_ = 3, *n*_SOX2-BS-∆M_ = 4, *n*_SOX2-BS-∆Bi_ = 3 independent biological replicates. **J** Graphical representation of *Gtl2* relative expression in mESC clones deleted for the ZFP281-binding site within the 315 bp of the IG-TRE, showing major reduction in *Gtl2* expression upon maternal deletion (*P*_∆M_ = 0.0002). **K** Graphical representation of *Gtl2* allelic expression in mESC clones deleted for the ZFP281-binding site within the 315 bp of the IG-TRE. **L** Graphical representation of the methylation at three regulatory elements at the Dlk1-Dio3 domain, analyzed by bisulfite pyrosequencing, shows increased methylation at the Gtl2-DMR upon deletion of the ZFP281-binding site from within the 315 bp of the IG-TRE from the maternal but not paternal chromosome (P_∆M_ = 0.05). *n*_WTBC_ = 3, *n*_ZFP281-BS-∆P_ = 4, *n*_ZFP281-BS-∆M_ = 5 independent biological replicates for expression analysis, and *n*_WTBC_ = 5, *n*_ZFP281-BS-∆P_ = 5, *n*_ZFP281-BS-∆M_ = 5 independent biological replicates for methylation analysis. Data are presented as mean values +/− SEM. NS not significant. Asterisks indicate statistical significance in comparison to wild-type using a two-tailed unpaired Student’s *t* test.

To determine the role of the transcriptional regulatory element in the regulation of expression at the Dlk1-Dio3 locus, we created mESCs deleted for the IG-TRE (Fig. 3A). We targeted 1.3 kb downstream of the IG-CGI, with two guide RNAs flanking this region, as defined by Aronson et al.^23^. Using SNPs between C57BL/6 and CAST/EiJ alleles in hybrid mESCs, we screened for clones with maternal, paternal, or biallelic deletions. In addition to the full 1.3 kb deletion, by chance we also obtained a smaller maternal deletion of 315 bp (Fig. 3B).

Maternal deletion of the entire 1.3 kb resulted in loss of maternal expression of *Gtl2*, *Rian*, and *Mirg* (Figs. 3D,E and  S2A–D, *Gtl2* *P*_∆M_ = 0.0002, *Rian* *P*_∆M_ = 0.02, *Mirg* *P*_∆M_ = 0.003), indicating that this region positively regulates the imprinted genes on the maternal chromosome. The same deletion on the paternal chromosome did not result in any change to gene expression at the Dlk1-Dio3 locus.

Furthermore, methylation analysis showed that loss of the maternal 1.3 kb IG-TRE resulted in increased methylation at the downstream Gtl2-DMR, while no change was observed in the upstream CpG island (IG-CGI) (Figs. 3F and  S3A–C, *P*_∆M_ = 1.3E^−05^), suggesting that the IG-TRE normally contributes to protection of the maternal chromosome from DNA methylation.

Deleting only 315 bp at the 5′ end of the TRE, had the same effect on maternal gene expression and methylation at the Gtl2-DMR as deleting the entire 1.3 kb on the maternal chromosome (Figs. 3D–F,  S2A–D and  S3A–C, *Gtl2* *P*_∆M-315bp_ = 4.1E^−06^, *Rian* *P*_∆M-315bp_ = 0.01, *Mirg* *P*_∆M-315bp_ = 3.02E^−05^, Gtl2-DMR *P*_∆M-315bp_ = 8.9E^−05^) indicating that the regulatory function of the IG-TRE is controlled by elements within that minimal 315 bp region.

### SOX2 and ZFP281 binding at the IG-TRE control maternal gene expression

To investigate which elements within the IG-TRE are responsible for maintaining the maternal epigenotype, we scanned the 315 bp region for transcription factor binding sites and identified highly conserved predicted binding sites for the SRY-Box Transcription Factor 2 (SOX2) and for the zinc finger protein 281 (ZFP281) (Fig. 3B, C).

We created mESC clones with targeted deletions for these transcription factor binding sites within the 315 bp of the IG-TRE (Fig. 3B). As a control, we also deleted the transcription factor binding site for PRDM14 (PR/SET Domain 14) from within the 315 bp of the IG-TRE, but found no change in expression or methylation at the region (Fig. S4).

Deletion of the SOX2-binding site on the maternal chromosome resulted in dysregulation of the maternal epigenotype, with decreased maternal gene expression (Figs. 3G, H and  S2E–H, *Gtl2* *P*_∆M_ = 0.004; *P*_∆Bi_ = 0.004, *Rian* *P*_∆M_ = 0.001; *P*_∆Bi_ = 0.001, *Mirg* *P*_∆M_ = 0.003; *P*_∆Bi_ = 0.007) and increased methylation at the IG-TRE and Gtl2-DMR (Figs. 3I and  S3D–F, IG-TRE *P*_∆M_ = 0.02; *P*_∆Bi_ = 0.03; Gtl2-DMR *P*_∆M_ = 8.7E^−06^; *P*_∆Bi_ = 0.001). No effect was observed with the deletion on the paternal chromosome.

Deletion of the ZFP281-binding site on the maternal chromosome also resulted in similar dysregulation of the maternal epigenotype, with decreased maternal gene expression (Figs. 3J, K and  S2I–L, *Gtl2* *P*_∆M_ = 0.0002, *Rian* *P*_∆M_ = 0.01, *Mirg* *P*_∆M_ = 0.004) and increased methylation at the Gtl2-DMR (Figs. 3L and  S3G–I, *P*_∆M_ = 0.05). No effect was observed with the deletion on the paternal chromosome.

These results indicate that SOX2 and ZFP281 binding play a key role in the *cis*-regulation of the maternally expressed genes in mESCs.

### The minimal 315 bp region of the IG-TRE, and SOX2 binding within it, has an enhancer-like function, while adjacent to it, there is an opposing silencing function

We dissected the previously identified 724 bp fragment into the 315 bp fragment, identified by our CRISPR deletion experiments, and 235 bp, which includes the rest of the downstream sequence, as well as a 550 bp fragment which encompasses both the 315 bp and 235 bp regions (Fig. 2A), and conducted luciferase assays in vitro to validate the ES cells' findings.

Similar to the 724 bp fragment, the minimal 315 bp fragment exhibited significant activity in the sense direction, indicating promoter-like function, as well as enhancer-like function in the promoter vector (Figs. 2D, E and  S1B, C, *P*_Basic_=1.01E^−07^,0.002; *P*_Promoter_=1.4E^−06^, 0.005; *P*_Enhancer_=2.6E^−06^, 0.002). However, when the SOX2-binding site was deleted from this minimal 315 bp fragment, this activity was lost (Figs. 2D, E and  S1B,C, *P*_Basic_=1.1E^−07^, 2.3E^−06^; *P*_Promoter_=1.5E^−06^, 0.0003; *P*_Enhancer_=5.9E^−06^, 0.001).

Strikingly, the 235 bp fragment, located downstream of the 315 bp region, exhibited the opposite silencing activity (Figs. 2D, E and  S1B, C, *P*_Basic_=6.2E^−16^, 1.03E^−16^; *P*_Promoter_=0.003,0.003; *P*_Enhancer_=9.95E^−11^, 3.04E^−09^). Finally, the 550 bp fragment exhibited a partially enhancing function, reflecting a combination of the enhancing elements from the 315 bp region and silencing from the 235 bp region (Figs. 2D, E and  S1B, C, *P*_Basic_=1.3E^−06^, 7.6E^−07^; *P*_Promoter_=0.0004; *P*_Enhancer_=3.02E^−13^, 5.7E^−08^).

We concluded that the 315 bp region at the IG-TRE is functionally important for expression of the imprinted noncoding RNAs from the maternal chromosome, and that this function is mediated by SOX2 binding. In contrast, the 235 bp region downstream of the 315 bp region contains a silencing element, and the combined 550 bp region is the minimal region of activity containing both regulatory elements at the IG-TRE.

### Maternal binding of ZFP281, together with SOX2 binding, regulates expression on the maternal chromosome

**Fig. 4:**
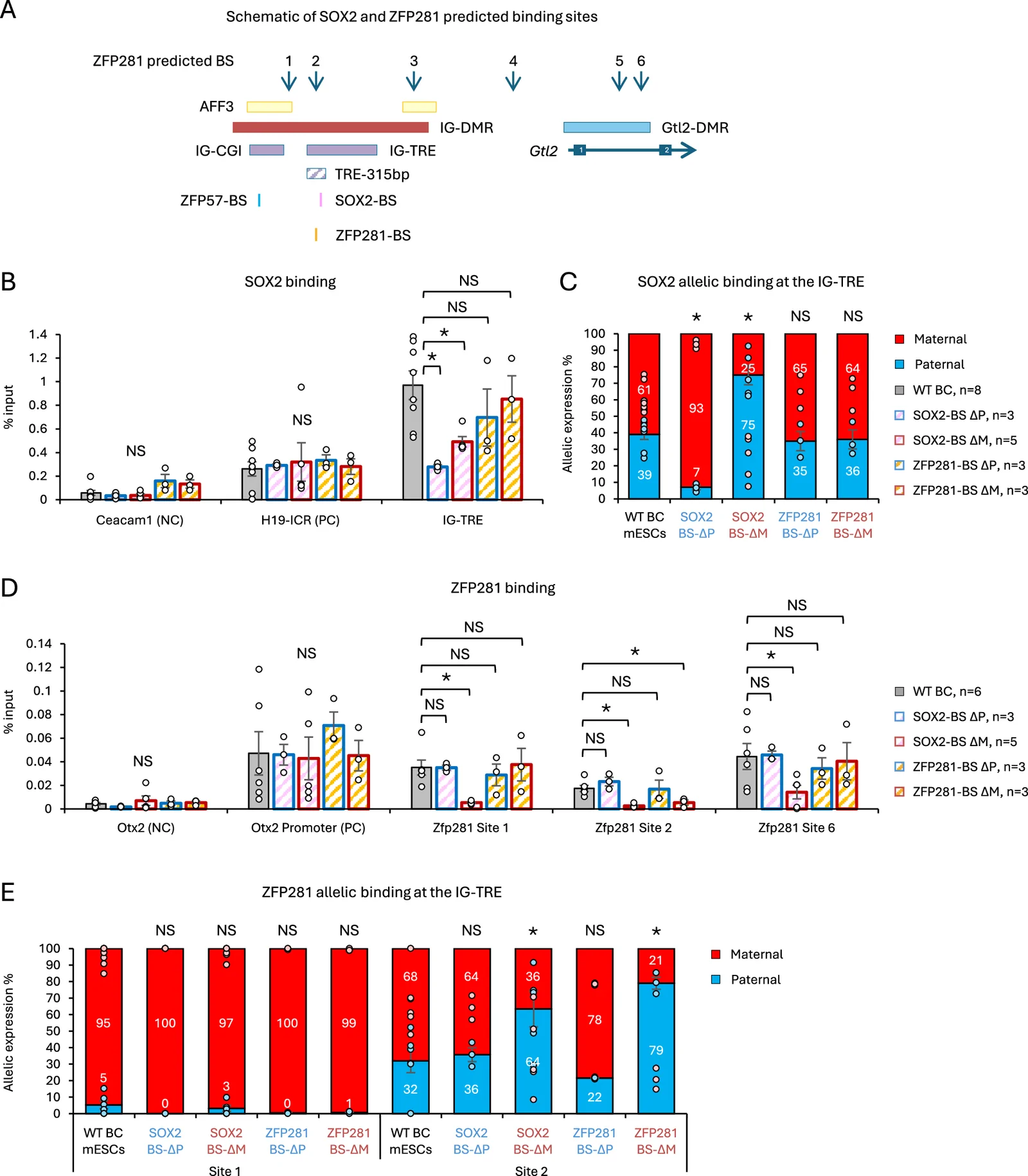
ZFP281 binding is dependent on SOX2 binding at the IG-TRE, and together they regulate expression on the maternal chromosome. **A** Schematic of SOX2 and ZFP281 predicted binding sites across the IG-DMR and Gtl2-DMR, assayed by ChIP-qPCR in (**B**–**E**). **B** Graphical representation of SOX2 binding at the IG-TRE, determined by ChIP-qPCR, shows depletion of SOX2 binding when the SOX2-binding site is deleted (as expected) but not when the ZFP281-binding site is deleted from within the 315 bp region (*P*_SOX2-BS-∆P_ = 0.008, *P*_SOX2-BS-∆M_ = 0.01). **C** Graphical representation of SOX2 allelic expression at the IG-TRE, as measured by ChIP-qPCR and pyro SNP analysis, shows biallelic binding in wild-type hybrid mESCs, while the maternal or paternal portions are depleted upon deletion of their respective SOX2-binding sites (as expected, *P*_SOX2-BS-∆P_ = 0.0001, *P*_SOX2-BS-∆M_ = 0.0001). No change in SOX2 allelic binding is observed upon deletion of either the maternal or paternal ZFP281-binding sites. *n*_WTBC_ = 8, *n*_SOX2-BS-∆P_ = 3, *n*_SOX2-BS-∆M_ = 5, *n*_ZFP281-BS-∆P_ = 3, *n*_ZFP281-BS-∆M_ = 3 independent biological replicates. **D** Graphical representation of ZFP281 binding at the IG-TRE, determined by ChIP-qPCR, shows depletion of ZFP281 binding at site 2 when this ZFP281-binding site is deleted (as expected, *P*_ZFP281-BS-∆M_ = 0.02), and depletion of ZFP281 binding at sites 1, 2, and 6 when the maternal SOX2-binding site is deleted from within the 315 bp region (*P*_SOX2-BS-∆M_ = 0.002, 0.001, 0.04). No change in ZFP281 binding is observed upon deletion of the paternal SOX2 or paternal ZFP281-binding sites. **E** Graphical representation of ZFP281 allelic binding at the IG-TRE, as measured by ChIP-qPCR and pyro SNP analysis, shows maternal-specific binding in wild-type hybrid mESCs, which is depleted upon deletion of the maternal SOX2 or maternal ZFP281-binding sites (*P*_ZFP281-BS-∆M_ = 0.04, *P*_SOX2-BS-∆M_ = 0.003). *n*_WTBC_ = 6, *n*_SOX2-BS-∆P_ = 3, *n*_SOX2-BS-∆M_ = 5, *n*_ZFP281-BS-∆P_ = 3, *n*_ZFP281-BS-∆M_ = 3 independent biological replicates. Data are presented as mean values +/− SEM. NS not significant. Asterisks indicate statistical significance in comparison to wild-type using a two-tailed unpaired Student’s *t* test.

In order to verify SOX2 and ZFP281 binding at the IG-TRE, and to determine whether they bind to one or both chromosomes, we performed chromatin immunoprecipitation and quantitative polymerase chain reactions (ChIP-qPCR) in wild-type, SOX2-BS mutant, and ZFP281-BS mutant hybrid mESCs (Fig. 4A).

Surprisingly, we found that in mESCs, SOX2 binds to both the maternal and paternal copies of the IG-TRE at the 315 bp region, with the maternal and paternal SOX2-binding site deletions resulting in loss of binding on the corresponding copy (Fig. 4B, *P*_SOX2-BS-∆P_ = 0.008, *P*_SOX2-BS-∆M_ = 0.01; and Fig. 4C, *P*_SOX2-BS-∆P_ = 0.0001, *P*_SOX2-BS-∆M_ = 0.0001). SOX2 binding was unchanged when the ZFP281-binding site was deleted from within the 315 bp of the IG-TRE. SOX2 binding at *Ceacam1* and the H19-ICR were assayed as negative and positive controls, respectively (Fig. 4B).

Since we found that SOX2 bound both parental chromosomes, we hypothesized that the maternal activity of the IG-TRE is combined with ZFP281 to confer allele specificity and regulate expression of the *Gtl2* polycistron^35^. We identified several ZFP281 predicted binding sites across the region in addition to the binding site we deleted from within the IG-TRE (predicted binding site#2, Fig. 4A): At the 3′ end of the IG-CGI, upstream of the IG-TRE (predicted binding site #1), downstream of the IG-TRE where it has previously been shown to bind and recruit AFF3 (predicted binding site #3)^35^, downstream to the IG-DMR (predicted binding site #4) and at the Gtl2-DMR (predicted binding sites #5,6)^35^. We assayed ZFP281 binding at these predicted binding sites and at two control regions in the *Otx2* gene (negative and positive controls).

We observed no binding at sites 3, 4, or 5 (Fig. S5), but that ZFP281 did bind at sites 1 and 2, upstream and in the 315 bp region, and at site 6 in the Gtl2-DMR (Fig. 4D), and that this binding was maternal-specific (Fig. 4E). As expected, ZFP281 binding was depleted at site#2 upon deletion of its binding site from within the 315 bp of the IG-TRE (Fig. 4D, *P*_site2_=0.02; Fig. 4E, *P*_site2_=0.003). Importantly, we found that ZFP281 binding was also lost upon deletion of the maternal SOX2-binding site at the 315 bp region (Fig. 4D, *P*_site1_=0.002, *P*_site2_=0.001, *P*_site6_=0.04; Fig. 4E, *P*_site2_=0.04), indicating that ZFP281 binding is dependent on SOX2 binding, but not the other way around, and together they regulate expression of the genes on the maternal chromosome. No change in ZFP281 binding was observed upon deletion of the paternal ZFP281 or SOX2-binding sites at the 315 bp region (Fig. 4D, E).

### Generation of IG-TRE knockout mice

**Fig. 5:**
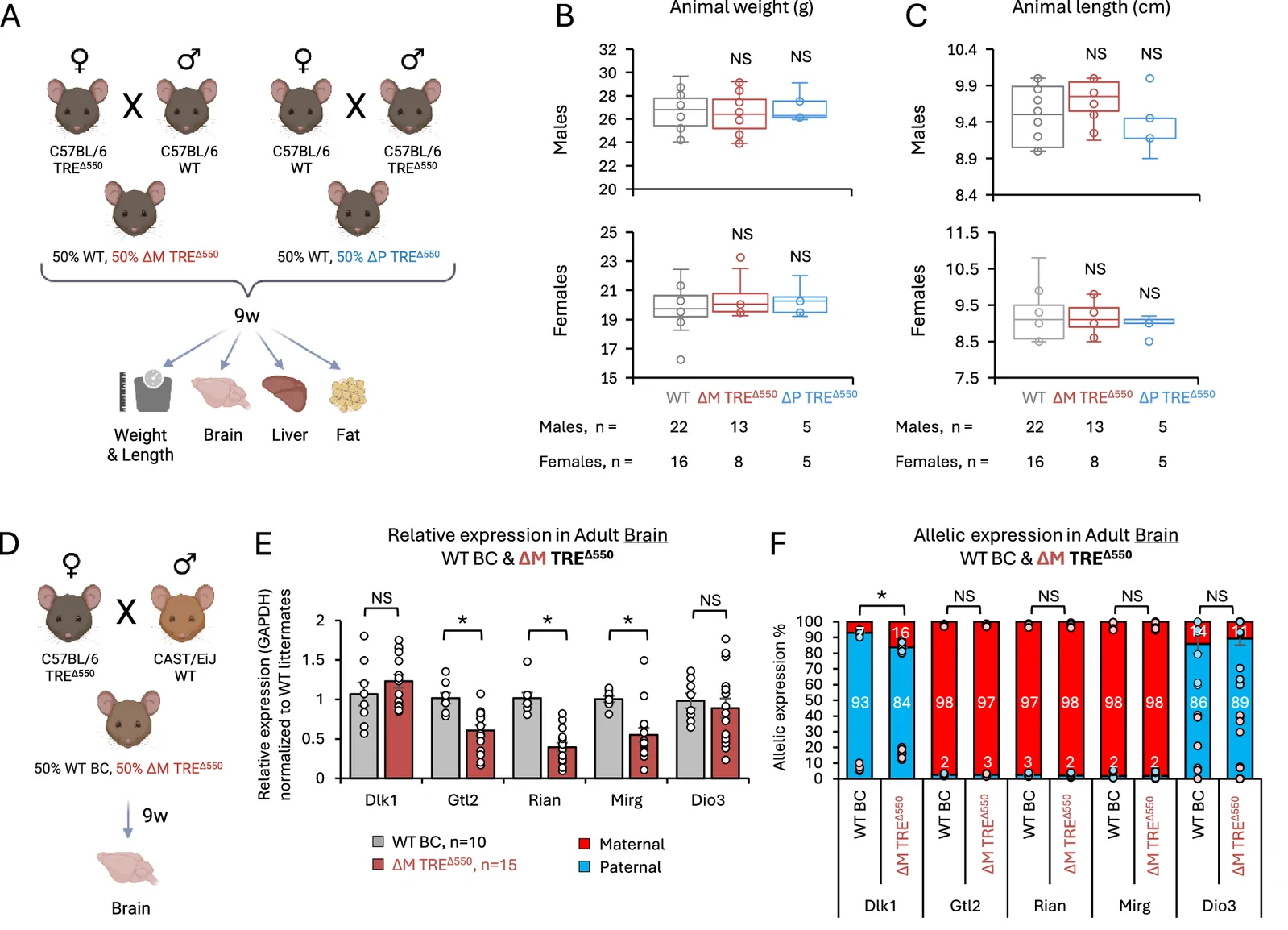
Deletion of the IG-TRE^∆550^ in vivo is viable and results in decreased maternal gene expression in adult brains. **A** Breeding strategy of the IG-TRE 550 bp deletion animals crossed with C57BL/6 partners to produce maternal or paternal inheritance of the IG-TRE^∆550^ deletion. In each litter, 50% of the pups are wild-type littermates (pure C57BL/6) and kept as controls. Tissues were collected for expression at 9 weeks. Created in BioRender. Edwards, C. (2026) https://BioRender.com/y4zqy25. **B** Box plot representation of total body weight (g) of 9-week-old adult animals. For WT, ∆P TRE^∆550^ and ∆M TRE^∆550^ males: Min = 24, 26, 23.9; Q1 = 25.4, 26.1, 25.2; Center line= 26.8, 26.3, 26.4; Q3 = 27.8, 27.5, 27.7; Max= 29.7, 29.1, 29.1; *n* = 22, 5, 13. For WT, ∆P TRE^∆550^ and ∆M TRE^∆550^ females: Min = 16.3, 19.2, 19.2; Q1 = 19.2, 19.5, 19.5; Center line = 19.7, 20.3, 20.1; Q3 = 20.7, 20.5, 20.8; Max= 22.4, 22, 23.3; *n* = 16, 5, 8. **C** Box plot representation of total body length (cm) of 9-week-old adult animals. For WT, ∆P TRE^∆550^ and ∆M TRE^∆550^ males: Min= 9, 8.9, 9.2; Q1 = 9.1, 9.2, 9.6; Center line = 9.5, 9.5, 9.8; Q3 = 9.9, 9.5, 9.9; Max= 10, 10, 10; *n* = 22, 5, 13. For WT, ∆P TRE^∆550^ and ∆M TRE^∆550^ females: Min= 8.5, 8.5, 8.5; Q1 = 8.6, 9, 8.9; Center line = 9.1, 9, 9.1; Q3 = 9.5, 9.1, 9.4; Max= 10.8, 9.2, 9.8; *n* = 16, 5, 8. **D** Breeding strategy of IG-TRE^Δ550^ females crossed with CAST/EiJ males to produce hybrid animals with maternal inheritance of the IG-TRE^Δ550^ deletion. In each litter, 50% of the pups are wild-type littermates (hybrid BxC) and kept as controls. Tissues were collected for expression at 9 weeks. Created in BioRender. Edwards, C. (2026) https://BioRender.com/b9fvzre. **E** Graphical representation of the relative expression of the Dlk1-Dio3 genes, as measured by qPCR, in adult brains of maternal IG-TRE^Δ550^ and BxC wild-type littermates (*P*_*Gtl2*_ = 0.001, *P*_*Rian*_ = 1.3E^−06^, *P*_*Mirg*_ = 0.001). **F** Graphical representation of the allelic expression of the Dlk1-Dio3 genes, as measured by pyro SNP analysis, in adult brains of maternal IG-TRE^Δ550^ and BxC wild-type littermates (*P*_*Dlk1*_ = 3.5E^−10^). Expression analysis shows that *Dlk1* maternal expression is increased, and *Gtl2*, *Rian*, and *Mirg* are decreased in maternal IG-TRE^Δ550^ adult brains. *n*_WTBC_ = 10, *n*_∆M-TRE_^∆550^ = 15 biologically independent adult brains were analyzed for gene expression. Data are presented as mean values +/− SEM. NS not significant. Asterisks indicate statistical significance in comparison to wild-type using a two-tailed unpaired Student’s *t* test.

To elucidate the functional roles of the SOX2 and ZFP281 binding at the IG-TRE in vivo during mouse development, we created knockout mice deleted for 550 bp of the IG-TRE, containing both functional elements that we identified in vitro: the minimal 315 bp enhancing region, and the 235 bp silencing region. Reciprocally, crossing IG-TRE^∆550^ F1 deletion animals with C57BL/6 or CAST/EiJ wild-type partners allowed us to determine parent-of-origin effects of this deletion at different developmental stages in a highly controlled and reproducible manner (Fig. 5A).

### Phenotype and imprinting effect of the IG-TRE 550 bp deletion in adult mice

Consistent with recent results^36^, we found that both maternal and paternal inheritance of the IG-TRE^∆550^ deletion produced viable and fertile offspring. At 9 weeks of age, the weights and lengths of both maternal and paternal deleted animals were indistinguishable from their wild-type littermates (Fig. 5B, C). No difference was observed in brain and fat weight either (Fig. S6A, C, D, F), however, maternal IG-TRE^∆550^ animals and paternal IG-TRE^∆550^ males had reduced liver weight (Fig. S6B, *P*_∆P-males_=0.05; *P*_∆M-males_=0.05; *P*_∆M-females_=0.04 and Fig. S6E, *P*_∆P-males_=0.01; *P*_∆M-males_=0.02; *P*_∆M- females_=0.03).

We performed allele-specific expression analysis on brains from 9-week-old hybrid animals harboring the maternal IG-TRE^∆550^ and their wild-type littermates (Fig. 5D). Expression of *Dlk1* from the maternally inherited chromosome, though low, was significantly increased when the IG-TRE^∆550^ deletion was on the maternally inherited chromosome compared to wild-type, where *Dlk1* is predominantly expressed from the paternal chromosome (Fig. 5E, F, *P* = 3.5E^−10^). Reciprocally, *Gtl2*, *Rian*, and *Mirg* were downregulated when the deletion was on the maternal chromosome compared to controls, similar to our observations in vitro (Fig. 5E, *P*_*Gtl2*_=0.001; *P*_*Rian*_=1.3E^−06^; *P*_*Mirg*_=0.002). No change in expression was observed in the brains of paternal IG-TRE^∆550^ animals, or in the liver of either maternal or paternal IG-TRE^∆550^ and their wild-type littermates (Fig. S7A–D). We next analyzed methylation levels associated with the three regulatory elements at the Dlk1-Dio3 region, the IG-CGI, IG-TRE and Gtl2-DMR, and found that they maintained normal levels of methylation in maternal and paternal IG-TRE^∆550^ adult brains compared to their wild-type littermates (Fig. S7E, F), indicating that the 550 bp on the maternally inherited IG-TRE is involved in regulating expression from the maternal chromosome in vivo, but that this function is tissue-specific, and its deletion neither changes the imprint at the ICR nor is detrimental for embryonic development.

### Tissue-specific dissection of IG-TRE^∆550^ during embryonic development

**Fig. 6:**
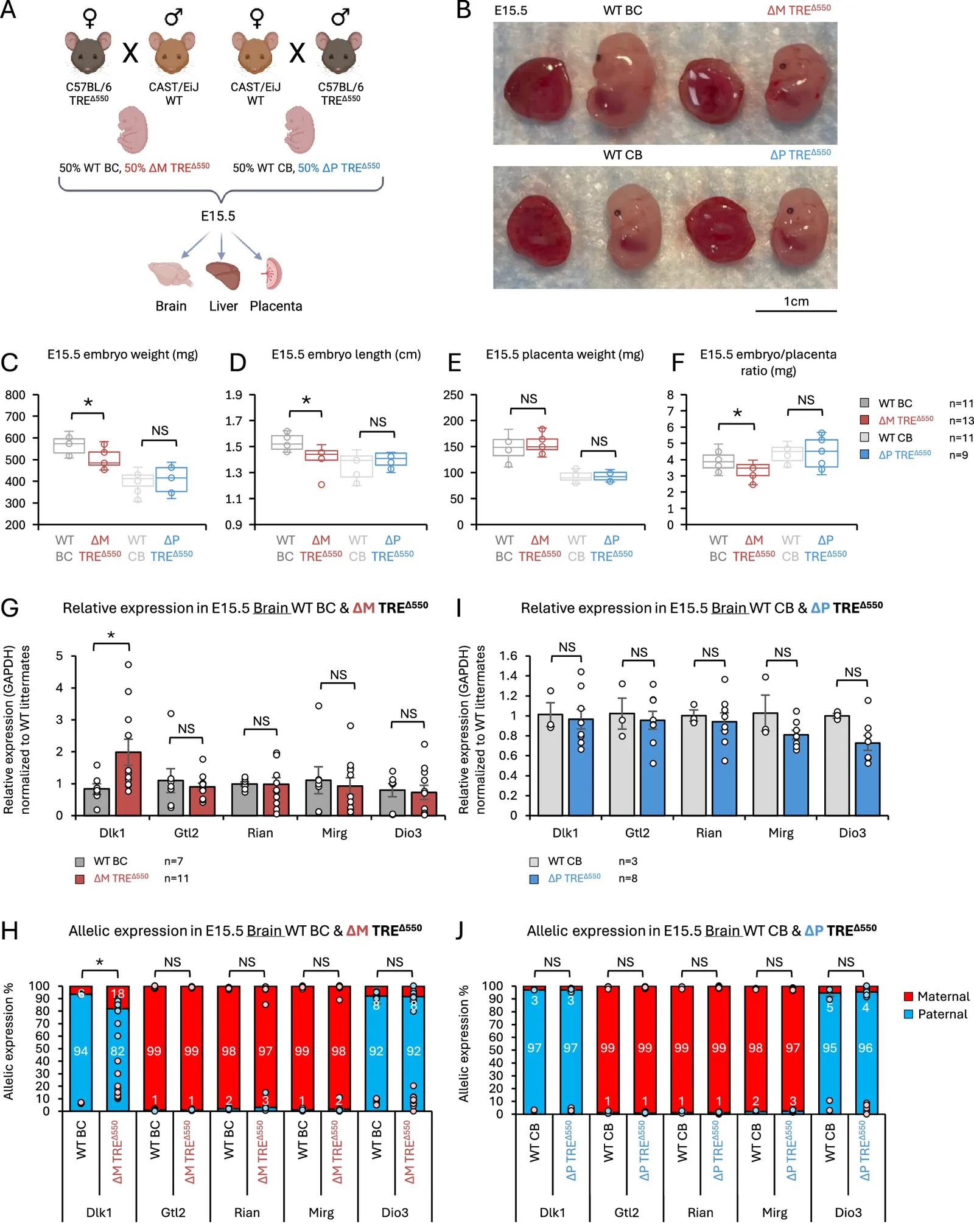
Deletion of the maternal, but not paternal, IG-TRE^∆550^ in vivo results in smaller embryos at E15.5, with increased maternal *Dlk1* expression in the brain, with no change in maternal gene expression. **A** Breeding strategy of IG-TRE^∆550^ animals crossed with CAST/EiJ partners to produce hybrid pups with maternal or paternal inheritance of the IG-TRE^∆550^ deletion. In each litter, 50% of the pups are wild-type littermates (hybrid BxC or CxB) and kept as controls. Tissues were collected for expression at E15.5. Created in BioRender. Edwards, C. (2026) https://BioRender.com/5n8dtw0. **B** Representative images of E15.5 embryos and placentas of all genotypes. Scale bar = 1 cm. **C** Box plot representation of E15.5 embryo weight (mg). For WTBC, ∆M TRE^∆550^, WTCB, and ∆P TRE^∆550^: Min = 506.8, 451.8, 307.2, 320.7; Q1 = 530.5, 475.2, 377.4, 352.2; Center line = 573.5, 484.3, 411.7, 415.8; Q3 = 595.6, 535.1, 428.6, 463; Max = 630, 583, 464.5, 488; *n* = 11, 13, 11, 9; *P* = 0.03. **D** Box plot representation of E15.5 embryo crown-rump length (cm). For WTBC, ∆M TRE^∆550^, WTCB, and ∆P TRE^∆550^: Min = 1.46, 1.21, 1.2, 1.3; Q1 = 1.48, 1.4, 1.27, 1.36; Center line= 1.52, 1.44, 1.39, 1.41; Q3 = 1.59, 1.47, 1.43, 1.45; Max= 1.62, 1.52, 1.48, 1.46; *n* = 11, 13, 11, 9; *P* = 0.002. **E** Box plot representation of E15.5 placenta weight (mg). For WTBC, ∆M TRE^∆550^, WTCB, and ∆P TRE^∆550^: Min = 110.9, 129.9, 78.7, 82.1; Q1 = 132.4, 144, 85.7, 86; Center line = 148.6, 149.7, 89.8, 92.1; Q3 = 163.6, 164.4, 99.2, 100.7; Max= 183.1, 185.6, 107.8, 106; *n* = 11, 13, 11, 9. **F** Box plot representation of E15.5 embryo/placenta weight ratio (mg). For WTBC, ∆M TRE^∆550^, WTCB, and ∆P TRE^∆550^: Min = 3, 2.4, 3.5, 3.1; Q1 = 3.5, 3, 3.9, 3.6; Center line = 3.9, 3.5, 4.5, 4.5; Q3 = 4.3, 3.7, 4.8, 5.2; Max = 5, 4, 5.1, 5.7; *n* = 11, 13, 11, 9; *P* = 0.01. **G**. Graphical representation of the relative expression of the Dlk1-Dio3 genes, as measured by qPCR, in E15.5 brains of maternal IG-TRE^∆550^ and BxC wild-type littermates (*P*_*Dlk1*_ = 0.04). **H** Graphical representation of the allelic expression of the Dlk1-Dio3 genes, as measured by pyro SNP analysis, in E15.5 brains of maternal IG-TRE^∆550^ and BxC wild-type littermates (*P*_*Dlk1*_ = 0.005). Expression analysis shows that *Dlk1* maternal expression is increased in maternal IG-TRE^∆550^ E15.5 brains. Other gene expression is unchanged. **I** Graphical representation of the relative expression of the Dlk1-Dio3 genes, as measured by qPCR, in E15.5 brains of paternal IG-TRE^∆550^ and CxB wild-type littermates. **J** Graphical representation of the allelic expression of the Dlk1-Dio3 genes, as measured by pyro SNP analysis, in E15.5 brains of paternal IG-TRE^∆550^ and CxB wild-type littermates. No change in gene expression is observed *n*_WTBC_ = 7, *n*_∆M-TRE_^∆550^ = 11, *n*_WTCB_ = 3, and *n*_∆P-TRE_^∆550^ = 8 biologically independent E15.5 brains were analyzed for gene expression. Data are presented as mean values +/− SEM. NS- not significant. Asterisks indicate statistical significance in comparison to wild-type using a two-tailed unpaired Student’s *t* test.

To further elucidate the effect of the IG-TRE^∆550^ deletion in a time-dependent manner, we crossed IG-TRE^∆550^ heterozygote animals with CAST/EiJ partners to dissect mutant embryos at E15.5 and analyzed gene expression and methylation in E15.5 embryonic brains upon maternal and paternal transmission of the deletion (Fig. 6A, B). We found that maternal IG-TRE^∆550^ E15.5 embryos were smaller than their wild-type littermates, while their placenta weight was unchanged (Fig. 6C–F, *P*_embryo-weight)_=0.003, *P*_embryo-length_=0.002, *P*_embryo/placenta-weight-ratio_= 0.02, and Fig. S8A–D). Similar to our observations from adult brains, we found that *Dlk1* expression was increased in maternal IG-TRE^∆550^ E15.5 brains compared to controls (Fig. 6G, *P* = 0.05) and that strikingly this expression came from both chromosomes (Fig. 6H, *P* = 0.005). Furthermore, surprisingly, and unlike adult brains and mESCs, there was no change in expression of the maternally expressed genes. *Dlk1* expression was also increased in maternal IG-TRE^∆550^ E15.5 liver (Fig. S8E, *P* = 0.04). No expression change of any gene at the Dlk1-Dio3 domain was observed in paternal IG-TRE^∆550^ E15.5 tissues compared to hybrid wild-type littermates (Figs. 6I, J and  S8G, H). Lastly, and consistent with the adult findings, we found no change in methylation at the IG-CGI, IG-TRE, or Gtl2-DMRs in E15.5 brains with either maternal or paternal deletion of the IG-TRE^∆550^ (Fig. S8I, J).

Taken together, these results indicate that the IG-TRE functions in a tissue and developmental stage-specific manner that may be associated with a requirement for exquisite *Dlk1* dosage control. For example, as opposed to its role as an enhancer of maternal *Gtl2* expression in mESCs and in adult brains where *Dlk1* is not strongly expressed, in vivo during embryonic development, where *Dlk1* expression is high in the brain, the IG-TRE has a more pronounced silencing effect on *Dlk1* expression, and its absence results in changes to *Dlk1* expression from both chromosomes (Figs. S8 and S9).

### SOX2 and ZFP281 bind to the maternal IG-TRE in adult brains in vivo

**Fig. 7:**
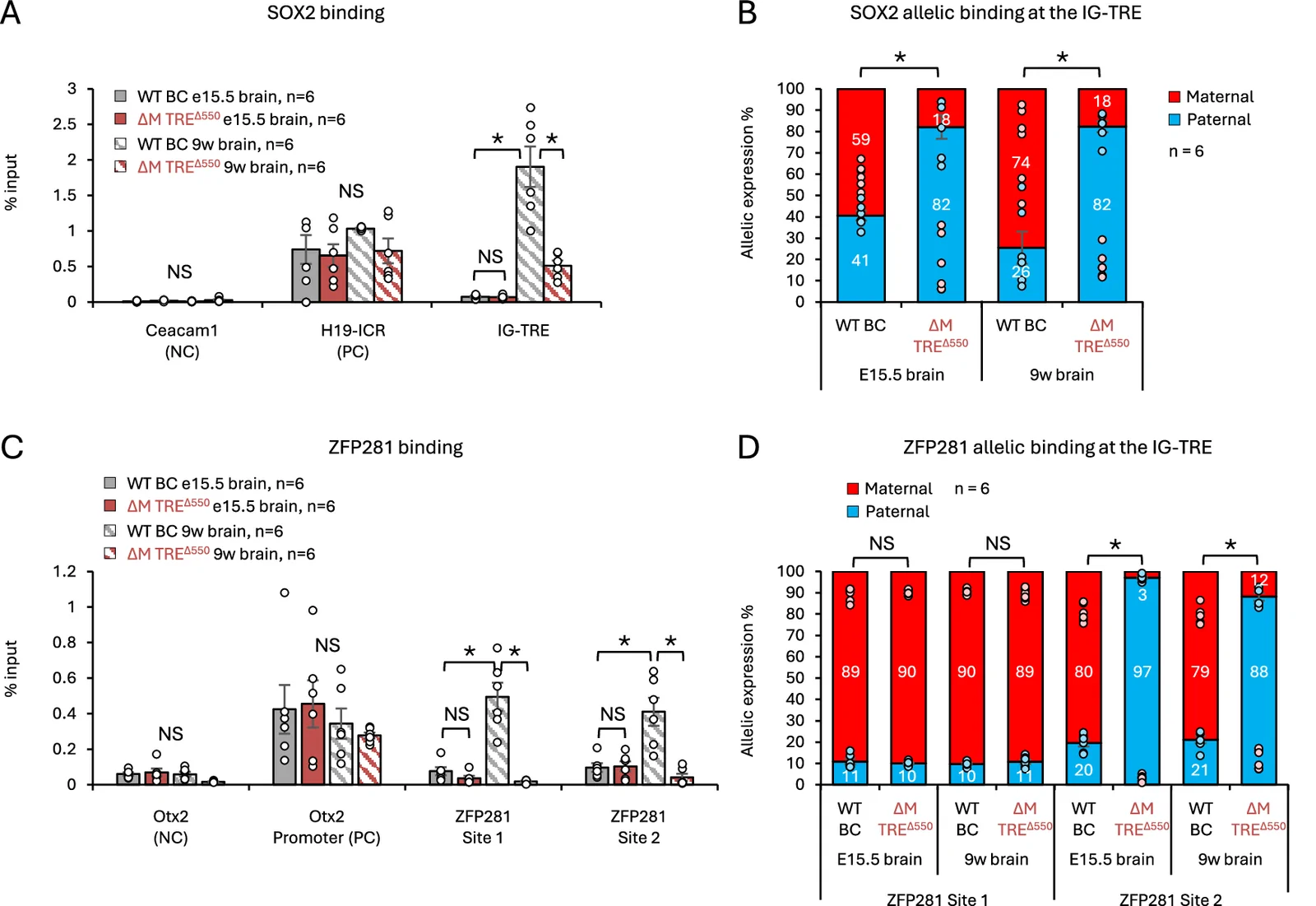
SOX2 and ZFP281 bind the maternal IG-TRE in adult brains in vivo. **A** Graphical representation of SOX2 binding at the IG-TRE in embryonic and adult brain, determined by ChIP-qPCR, shows more binding in adult vs embryonic brains (*P* = 0.0001), and that SOX2 binding is reduced when the maternal IG-TRE^Δ550^, including the SOX2-binding site, is deleted (*P* = 0.0008). **B** Graphical representation of SOX2 allelic expression at the IG-TRE, as measured by ChIP-qPCR and pyro SNP analysis, shows primarily maternal binding in wild-type hybrid brains, and depletion of the maternal portion upon deletion of the maternal IG-TRE^Δ550^, which includes the SOX2-binding site (*P*_E15.5_ = 0.00004, *P*_9w_ = 0.00003). **C** Graphical representation of ZFP281 binding at the IG-TRE in embryonic and adult brain, determined by ChIP-qPCR, shows increased binding in adult vs embryonic brains (*P*_site1_ = 0.0005, *P*_site2_ = 0.003), and that ZFP281 binding is depleted when the maternal IG-TRE^Δ550^, including the SOX2-binding site, is deleted (*P*_site1_ = 0.0001, *P*_site2_ = 0.001). **D** Graphical representation of ZFP281 allelic expression at the IG-TRE, as measured by ChIP-qPCR and pyro SNP analysis, shows primarily maternal binding in wild-type hybrid brains, which is depleted upon deletion of the maternal IG-TRE^Δ550^, including the SOX2-binding site (*P*_E15.5_ = 1.2E^−12^, *P*_9w_ = 1.3E^−10^). *n* = 6 biologically independent brains were analyzed for each stage and genotype. Data are presented as mean values +/− SEM. NS not significant. Asterisks indicate statistical significance in comparison to wild-type using a two-tailed unpaired Student’s *t* test.

To explain the observed differences between the functionality of our IG-TRE^∆550^ deletion in embryonic and adult brains, we assayed the binding of SOX2 and ZFP281 in vivo. We found that both SOX2 and ZFP281 bind to the IG-TRE in 9-week-old brains, but that binding is weak in embryonic brains (Fig. 7A, C, *P*_SOX2_ = 0.0001, *P*_ZFP281-site1_=0.0005, *P*_(ZFP281-site2)_=0.003). Furthermore, as opposed to the biallelic binding of SOX2 that we observed in ESCs, in vivo both SOX2 and ZFP281 binding was primarily maternal, and binding of both was lost in IG-TRE^∆550^ maternal deletion brains (Fig. 7A, *P*_SOX2_ = 0.001; Fig. 7B, *P*_E15.5_ = 3.8E^−05^, *P*_9w_=3.3E^−05^; Fig. 7C, *P*_ZFP281-site1_=0.0001, *P*_ZFP281-site2_=0.001; Fig. 7D, *P*_E15.5_ = 1.2E^−12^, *P*_9w_=1.3E^−10^).

Together, these findings support a model in which SOX2 binding at the IG-TRE contributes to ZFP281 recruitment and maternal gene regulation in the adult brain, whereas this regulatory mechanism appears to be absent, less prominent, or not yet established in the embryonic brain. While the maternal silencing of *Dlk1* is already functional in utero, it is probably mediated by other elements within the 235 bp of the IG-TRE.

### Balancing Dlk1 gene dosage by combining two IG-DMR mouse deletion models

**Fig. 8:**
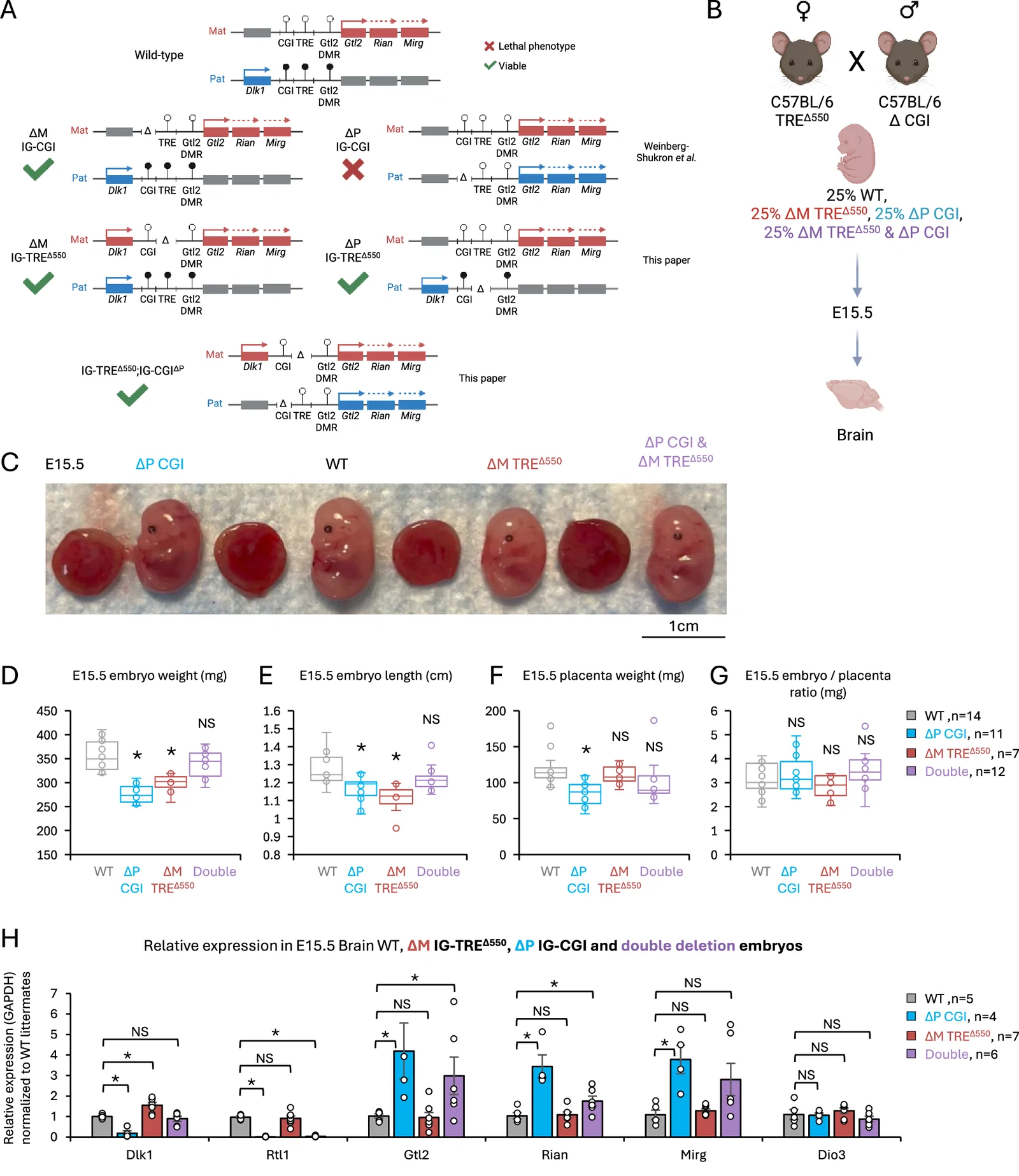
Deletion of the maternal IG-TRE^∆550^ rescues the lethality of the paternal IG-CGI deletion in vivo and results in balanced *Dlk1* gene dosage at the Dlk1-Dio3 domain. **A** Schematic representation of the predicted outcome of crossing ∆P-IG-CGI and ∆M-IG-TRE^Δ550^ mouse strains. The increased *Dlk1* expression caused by the maternal IG-TRE^Δ550^ deletion balances the loss of *Dlk1* expression caused by the IG-CGI paternal deletion and results in viable double deletion pups. Created in BioRender. Edwards, C. (2026) https://BioRender.com/al7up5v. **B** Breeding strategy of IG-TRE^Δ550^ heterozygote females with IG-CGI^Δ^ heterozygote males to produce double deletion pups. In each litter, 25% of the pups are wild-type littermates (pure C57BL/6) and kept as controls. 25% of the pups are IG-CGI^∆P^, which is lethal postnatally (as described in Weinberg-Shukron et al.^30^). 25% of the pups are IG-TRE^Δ550^ and 25% have both IG-CGI^∆P^ and IG-TRE^Δ550^. Tissues were collected for expression at E15.5. Created in BioRender. Edwards, C. (2026) https://BioRender.com/zuv6248. **C** Representative images of E15.5 embryos and placentas of all genotypes. Scale bar = 1 cm. **D** Box plot representation of E15.5 embryo weight (mg). For WT, ∆P CGI, ∆M TRE^∆550^ and double deletion: Min =  317.2, 252.1, 259.1, 290.2; Q1 = 327.7, 259.6, 290.6, 313.6; Center line =  349.5, 273.4, 302, 344.4; Q3 = 385.1, 292.2, 312.4, 361.4; Max= 410.6, 308.9, 319.4, 380.4; *n* = 14, 11, 7, 12; *P*_∆P-CGI_ = 2.8E^−07^, *P*_∆M-TRE_ = 0.0004. **E** Box plot representation of E15.5 embryo crown-rump length (cm). For WT, ∆P CGI, ∆M TRE^∆550^, and double deletion: Min= 1.1, 1, 0.9, 1.1; Q1 = 1.2, 1.1, 1.1, 1.2; Center line= 1.2, 1.2, 1.1, 1.2; Q3 = 1.3, 1.2, 1.2, 1.2; Max= 1.5, 1.3, 1.2, 1.4; *n* = 14, 11, 7, 12; *P*_∆P-CGI_ = 0.003, *P*_∆M-TRE_ = 0.001. **F** Box plot representation of E15.5 placenta weight (mg). For WT, ∆P CGI, ∆M TRE^∆550^, and double deletion: Min = 93.3, 56.6, 90.5, 71; Q1 = 106.6, 71.1, 102.5, 85.5; Center line = 113.7, 87.1, 107.6, 89.5; Q3 = 120.7, 97.5, 121.6, 109.4; Max = 178.7, 109.6, 130.6, 186.4; *n* = 14, 11, 7, 12; *P*_∆P-CGI_ = 0.001. **G** Box plot representation of E15.5 embryo/placenta weight ratio (mg). For WT, ∆P CGI, ∆M TRE^∆550^, and double deletion: Min = 2, 2.3, 2.1, 2; Q1 = 2.8, 2.7, 2.4, 3.1; Center line = 3, 3.1, 2.8, 3.4; Q3 = 3.8, 3.9, 3.1, 3.9; Max = 4.1, 5, 3.4, 5.4; *n* = 14, 11, 7, 12. While single deletion embryos are lighter and shorter in length, double deletion embryos are comparable to wild-type. **H** Graphical representation of the relative expression of the Dlk1-Dio3 genes, as measured by qPCR. *Dlk1* gene expression in double deletion E15.5 brains is not significantly different from wild-type, while *Rtl1* and the maternal genes remain decreased and upregulated, respectively (*P*_*Rtl1*_ = 1.75E^−08^, *P*_*Gtl2*_ = 0.08, *P*_*Rian*_ = 0.04, *P*_*Mirg*_ = 0.09). IG-CGI^∆P^ embryos show a paternal-to-maternal epigenotype switch, as previously described, with a downregulation of *Dlk1* and *Rtl1* and upregulation of the maternal transcripts *Gtl2*, *Rian*, and *Mirg* (*P*_*Dlk1*_ = 0.0002, *P*_*Rtl1*_ = 1.02E^−06^, *P*_*Gtl2*_ = 0.04, *P*_*Rian*_ = 0.003, *P*_*Mirg*_ = 0.005). Maternal IG-TRE^Δ550^ embryos show an upregulation of *Dlk1* and no change in other genes (*P*_*Dlk1*_ = 0.01). *n*_WT_ = 5, *n*_∆P-CGI_ = 4, *n*_∆M-TRE_^∆550^ = 7, and *n*_∆P-CGI&∆M-TRE_^∆550^ = 6 biologically independent E15.5 brains were analyzed for gene expression. Data are presented as mean values +/− SEM. NS not significant. Asterisks indicate statistical significance in comparison to wild-type using a two-tailed unpaired Student’s *t* test.

In light of our findings that the IG-TRE 550 bp deletion from the maternal chromosome results in increased *Dlk1* expression, but that the animals have no apparent phenotype, we asked whether this increased expression was significant enough to rescue a postnatal phenotype that arises from loss of *Dlk1* expression on the opposite chromosome. Previously we described that deleting the IG-CGI from the paternal chromosome results in an epigenotype switch from paternal to maternal like properties^30^, with downregulation of *Dlk1* and upregulation of the maternal transcripts *Gtl2*, *Rian*, and *Mirg* (Fig. 8A). Furthermore, we showed that balancing the gene dosage, through flipping expression of paternally expressed genes from the maternal chromosome and maternally expressed genes from the paternal chromosome, was sufficient for normal development and viability. However, it remains unknown whether this is due to a balanced dosage of a specific gene or a combination of multiple/all genes at the Dlk1-Dio3 domain. The IG-TRE^∆550^ model described here offers a rare opportunity to address the possibility that the paternal or maternal genes at the Dlk1-Dio3 domain are not equally essential for viability. Specifically, crossing IG-TRE^∆550^ heterozygote females (increased *Dlk1*) with IG-CGI^∆^ heterozygote males (loss of *Dlk1*) is expected to produce double deletion embryos with a balance of *Dlk1* expression, while the maternal genes will remain dysregulated (Fig. 8A). We hypothesized that inheritance of both deletions, would rescue the postnatal lethality of the paternal IG-CGI deletion if balancing *Dlk1* expression was sufficient for development, but not if balance of the maternal transcripts was essential.

### Viability of IG-TRE^∆550^ and IG-CGI^∆P^ double deletion pups

Interestingly, we found that while isolated IG-CGI^∆P^ pups died postnatally as expected, double deletion pups were viable and overall indistinguishable from their wild-type and maternal IG-TRE^∆550^ littermates (Fig. S10A–I and Table S7). Notably, IG-TRE^∆550^;IG-CGI^ΔP^ double deletion females had slightly reduced brain weights (Fig. S10C, *P* = 0.02) and about 50% more fat (Fig. S10E, *P* = 0.05) than wild-type females at 9 weeks of age, but this was not statistically significant when normalized to body weight (Fig. S10G, I).

### Imprinting analysis of IG-TRE^∆550^;IG-CGI^∆P^ double deletion embryos

To understand the regulatory configuration that allows these animals to develop normally, we dissected embryos at E15.5 and analyzed gene expression in embryonic brains (Fig. 8B, C). We found that, similar to our previous observations for isolated paternal IG-CGI^∆P^ and maternal IG-TRE^∆550^ embryos, mutant E15.5 embryos were smaller than their wild-type littermates, while IG-TRE^∆550^ placenta weight was unchanged (Fig. 8D, *P*_∆P-CGI_ = 2.8E^−07^, *P*_∆M-TRE_ = 0.0004; Fig. 8E, *P*_∆P-CGI_ = 0.003, *P*_∆M-TRE_ = 0.001; Fig. 8F, *P*_∆P-CGI_ = 0.001, and Fig. S11). IG-TRE^Δ550^;IG-CGI^ΔP^ double deletion embryos however, were not significantly different to their wild-type littermates. Gene expression analysis confirmed the previously reported gene dosage effects of the isolated paternal IG-CGI^ΔP^ and maternal IG-TRE^∆550^ deletions, with the former exhibiting lower levels of *Dlk1* and *Rtl1* relative to wild-type littermates together with elevated levels of all maternal transcripts at this locus (Fig. 8H, *P*_*Dlk1*_=0.0002; *P*_*Rtl1*_=1.02E^−06^; *P*_*Gtl2*_=0.04; *P*_*Rian*_=0.003; *P*_*Mirg*_=0.005), while the latter shows increased *Dlk1* expression and no change to the maternal genes (Fig. 8H, *P*_*Dlk1*_=0.01). Importantly, we found that *Dlk1* expression is restored to normal levels in IG-TRE^Δ550^; IG-CGI^ΔP^ double deletion embryos, while *Rtl1* remained low and maternal gene expression remains elevated (Fig. 8H, *P*_*Rtl1*_=1.8E^−08^; *P*_*Gtl2*_=0.08; *P*_*Rian*_=0.04; *P*_*Mirg*_=0.09).

Combining the results of all the ICR deletion models shows that loss of both the paternal genes *Dlk1* and *Rtl1*, as in the IG-CGI^∆P^ model, or the maternal genes *Gtl2*, *Rian*, and *Mirg*, as in the IG-DMR^∆M^ model, does not allow for normal development. Increased *Dlk1* expression, however, as in the maternal IG-TRE^∆550^ model, has no obvious mutant phenotype, suggesting that overexpression is not as detrimental as loss of expression. Moreover, reintroducing *Dlk1* expression by combining the maternal IG-TRE^∆550^ and the IG-CGI^∆P^ deletions, elevates *Dlk1* sufficiently to rescue the lethality of the IG-CGI^∆P^ alone.

Our results, therefore highlight a fundamental requirement for expression of the Dlk1-Dio3 genes from at least one chromosome, regardless of parental origin, for proper embryonic growth and survival, and that the lethality observed in the IG-CGI^∆P^ and IG-DMR^∆M^ deletion models is due to loss of expression of * Dlk1* and not overexpression of the opposite maternally expressed noncoding RNA genes.

## Discussion

Appropriate allele-specific expression of the genes at the Dlk1-Dio3 region is necessary for normal mammalian development. Understanding the regulation of genomic imprinting at the Dlk1-Dio3 domain requires in vivo models that faithfully recapitulate developmental complexity. Assessing different models that remove various elements in the region has allowed us to dissect the chain of events that is necessary to establish and maintain imprinted gene expression.

While a full deletion of the IG-DMR resulted in a maternal-to-paternal epigenotype and allelic-expression switch, an isolated deletion of the IG-CGI alone resulted in the reciprocal paternal-to-maternal switch. The remaining part of the IG-DMR, the IG-TRE, has been suggested to control maternal expression, but this was not demonstrated in vivo until recently^36^.

Here, we defined the minimal region of enhancer activity required for maternal epigenotype maintenance to 315 bp at the 5′ of the IG-TRE and found that this activity is dependent on the binding of SOX2 and ZFP281. Maintenance of hypomethylation at the maternal H19-ICR is also known to involve SOX/OCT factors^42^. We show that the function of the maternal IG-TRE is similarly mediated by SOX2 binding, and when the SOX2-binding site was deleted, the maternal IG-TRE could not bind ZFP281, leading to decreased expression of the maternal gene *Gtl2*. We showed that these elements also bind to the IG-TRE in vivo, with increased binding in the adult brain compared to the embryonic brain.

However, our IG-TRE^Δ550^ deletion, which includes the 315 bp region, resulted in no overt phenotype in mice, highlighting a divergence between in vitro and in vivo imprinting control. Our in vitro assays also identified a silencing element within the IG-TRE, but since *Dlk1* is not expressed in ESCs we could not test the complete effects of this region in culture, further emphasizing the importance of studying these elements in vivo. While embryonic stem cell-based assays have provided insights into transcription factor binding and epigenetic regulation, our data demonstrate that in vitro systems do not fully capture the functional consequences of regulatory element deletions in vivo. Similar discrepancies were previously reported for AFF3/ZFP281 activity, where this binding regulated maternal expression in ESCs but not in vivo^27,35^, suggesting that the effect of ZFP281 binding at the IG-DMR is temporal and cell-type specific. Similarly, we also observed differences in allelic binding of SOX2, where it bound to both chromosomes in ESCs, but was primarily maternal-specific in brain tissue.

**Fig. 9:**
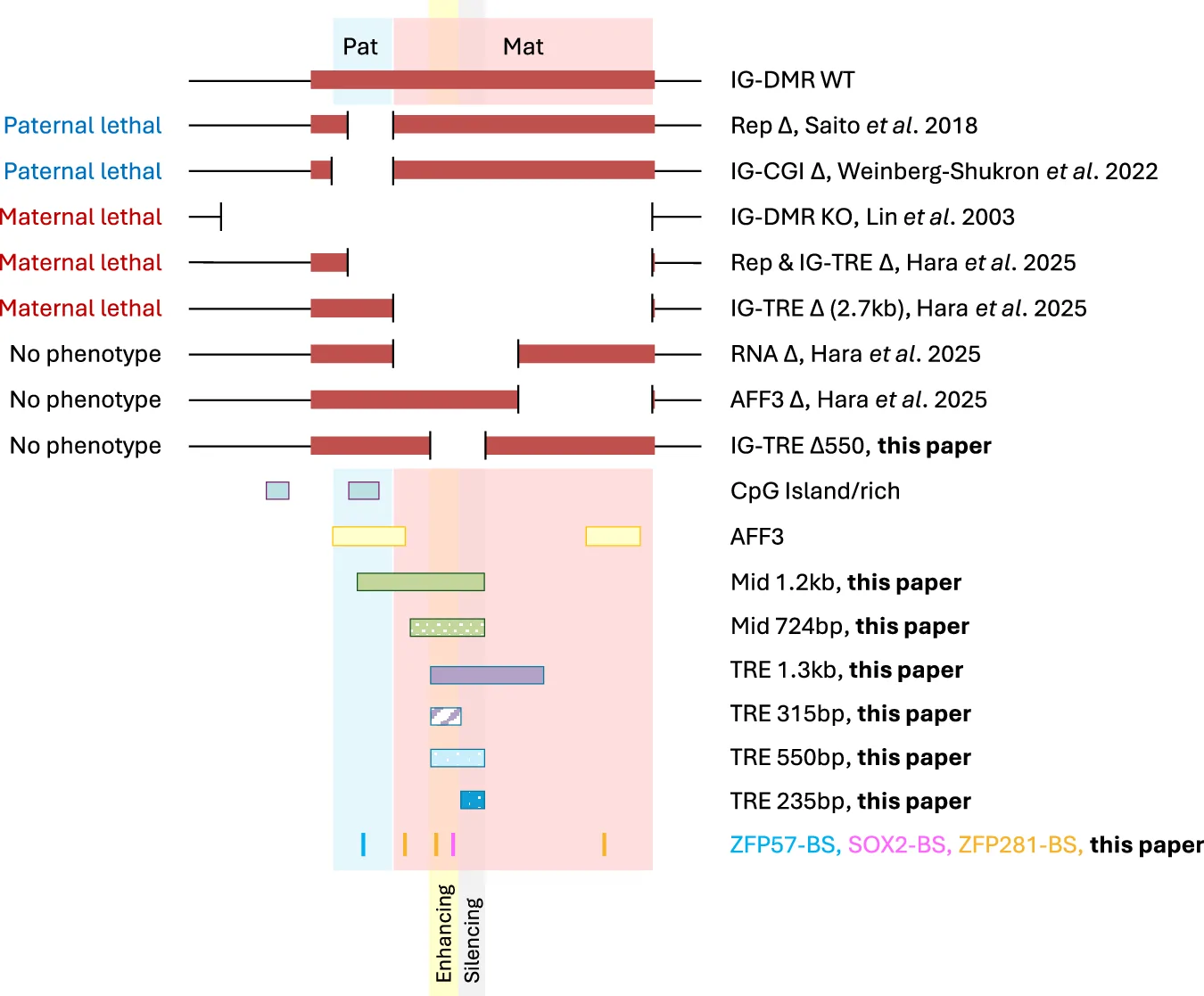
An integrated summary depicting the allele-specific counterparts involved in *cis*-regulation at the Dlk1-Dio3 region. Regulation of the paternal epigenotype involves the CpG island and ZFP57 binding site (BS). Maternal regulation is more complex and involves multiple transcriptional regulatory elements (TRE) downstream to the CpG island, including AFF3, SOX2, and ZFP281 binding. Deletions of these elements on their own (RNA ∆ model, AFF3 ∆ model, or the IG-TRE^Δ550^ model in this paper) have no phenotype and do not dysregulate imprinting on the maternal chromosome. The minimal region of 550 bp at the IG-TRE includes dual enhancing and silencing activity, positively driving maternal gene expression and reducing paternal *Dlk1* expression from the maternal chromosome.

The 550 bp IG-TRE deletion described here did not recapitulate the maternal lethality of the full IG-DMR deletion^20^ or the 2.7 kb IG-TRE deletion^36^ in vivo, enabling refinement of the functional footprint of the IG-TRE by targeting a smaller element than in previous studies (Fig. 9). Our results show that deletion of the IG-TRE^Δ550^ in adult brains results in reduced maternal expression, suggesting that this region of the IG-TRE does have enhancer function on the maternal chromosome (similar to our observations in vitro), but that in vivo this is not required during embryonic development. We also found that SOX2 and ZFP281 bind at the IG-TRE in vivo, and that their binding in adults and not in utero explains the observed differences in gene expression caused by the IG-TRE^Δ550^ between these stages. Taken together, these results suggest that the IG-TRE controls dosage at the Dlk1-Dio3 region in a time-specific manner. In the embryo, when SOX2 and ZFP281 do not bind at the IG-TRE and *Dlk1* levels are higher, the silencing function modulates *Dlk1* dosage. In contrast, in the adult brain and in mESCs, when total *Dlk1* expression is low, the IG-TRE functions as an activator of *Gtl2*, mediated by SOX2 and ZFP281 binding (Fig. 9). Further experiments deleting the SOX2 and/or ZFP281-binding sites will be able to directly test this model in vivo.

Furthermore, while the 550 bp IG-TRE deletion did not disrupt maternal gene expression in utero, it caused a significant upregulation of *Dlk1* from both chromosomes, suggesting that the silencing element in the deleted region contributes to dosage restraint in *cis* and in *trans*, albeit redundant with other elements regulating maternal gene expression, thus allowing normal development.

This finding points to a trans-allelic regulatory effect at the Dlk1-Dio3*Dlk1-Dio3* locus, whereby deletion of a regulatory element on the maternal chromosome results in increased expression from the paternal allele. Rather than acting solely in *cis*, the IG-TRE appears to participate in inter-allelic communication, indicating that imprinting control at this locus involves coordinated regulation across both parental chromosomes. Such trans-homolog interaction is reminiscent of the communication between the maternally expressed microRNAs that are expressed antisense to the paternally expressed *Rtl1* gene and that regulate it in an RNAi-dependent manner due to their 100% complementarity^43^.

*Gtl2* RNA itself has been implicated in inter-allelic communication previously, however further interrogation of this is required in our models in a cell and stage-specific manner. Such cross-chromosomal influence adds an additional layer of complexity to imprinting regulation and may help explain why perturbations at ICRs often produce reciprocal effects that are difficult to interpret in terms of simple *cis*-acting mechanisms alone.

Our results support the notion that imprinting is regulated in a tissue-dependent manner, as the effects of the IG-TRE^∆550^ deletion differ between brain and liver. We further recognize that cellular heterogeneity within tissues may result in further nuanced differences. In particular, distinct cell populations within the brain may be subject to different regulatory mechanisms governing imprinting at this locus. Additional experiments may further elucidate this.

We also found that maternal IG-TRE^∆550^ animals have reduced liver weights, but no change in brain or fat weight. However, these observations are correlative and do not support a direct functional conclusion at this stage. Further work would be required to determine whether this reflects a direct tissue-specific effect of the IG-TRE^∆550^ deletion or an indirect consequence of broader physiological changes.

Importantly, our double deletion model, which combined the maternal IG-TRE 550 bp deletion with our previously characterized paternal IG-CGI deletion, provided functional evidence for regulatory compensation between maternal and paternal control elements. While the paternal IG-CGI deletion alone results in loss of imprinting, reducing *Dlk1* expression, increasing expression of the usually paternally repressed long noncoding RNA genes, and leading to embryonic lethality, this phenotype is fully rescued by simultaneous deletion of the maternal IG-TRE^∆550^. These findings support a model in which loss of expression is more detrimental than gain, suggesting that precise regulation of *Dlk1* is required within a narrow threshold range, and that modest overexpression is better tolerated than deficiency.

It is important to note that *Dlk1* knockout mice are not lethal but display metabolic phenotypes as well as overall growth retardation^44^. Hence, the lethality of the IG-CGI paternal deleted pups could not be attributed to loss of *Dlk1* expression alone. The maternalization of the paternal chromosome by the IG-CGI deletion results in loss of expression of other paternal genes as well, such as *Rtl1* and *Dio3*, and their contribution to the lethality of these embryos should also be considered.

Similarly, increased *Dlk1* expression alone is unlikely to fully explain the growth restriction observed following the IG-TRE^∆550^ deletion, either, since *Dlk1* overexpression in BAC transgenic mice causes the opposite embryonic overgrowth^45^. Given that the IG-TRE^Δ550^ deletion perturbs the expression of multiple genes within the Dlk1-Dio3 domain, the growth restriction observed here likely reflects the combined effects of altered regulation across the imprinted locus rather than the isolated consequences of *Dlk1* dosage. These findings underscore the importance of maintaining a precise balance of gene dosage within the Dlk1-Dio3 domain for normal development.

Finally, although our results from embryonic brain show that, unlike *Dlk1*, *Rtl1* expression is not restored by the IG-TRE^Δ550^ deletion and remains reduced in the rescued IG-TRE^Δ550^;IG-CGI^ΔP^ double mutants, it is important to note that *Rtl1* expression levels are very low in embryonic tissues and are largely restricted to the placenta. Further experiments focusing on expression in the placenta may elucidate the contribution of *Rtl1* to the IG-CGI^ΔP^ phenotype.

Together, this work refines the regulatory map of the Dlk1-Dio3 domain and identifies transcription factor interactions at the IG-TRE and both enhancing and silencing elements. It provides in vivo evidence for epigenetic redundancy of cis-regulatory elements at this region and underscores the necessity of in vivo models for deciphering the complex chain of epigenetic events that are required to establish and maintain imprinted gene expression throughout development.

## Methods

### Cloning and generation of luciferase vectors

#### Polymerase chain reaction (PCR)

PCR fragments were amplified using Novagen KOD Hot Start DNA polymerase. In total, 1 µl of gDNA from C57BL/6J and CAST/EiJ mESCs hybrids was used as template in a 20 µl reaction containing 1× PCR Buffer (KOD Hot Start, Novagen #71155), 200 µM dNTPs (Novagen #71154), 1.8 mM MgSO_4_ (Novagen #71156), 0.6 mM DMSO, 5.8 mM cresol red, 0.5U of Hot Start KOD polymerase (Novagen #71316), and 0.6 µM of each primer. Primers introduced NheI or KpnI restriction sites at each end in order to clone both in sense and antisense orientation. The PCR program was 95 °C for 2′, followed by 35 cycles at 95 °C for 20″, 60 °C for 15″ and 70 °C for 20″. The PCR products were visualized by agarose gel electrophoresis in TBE buffer and stained with SafeView (NBS Biologicals #NBS-SV). Primers used to generate the luciferase constructs are listed in Table S1.

#### A-tailing of PCR products

KOD PCR products were A-tailed prior to cloning. Each 25 µl reaction contained 12.5 µl PCR product, 1× HotStarTaq master mix (Qiagen #1005479), 0.2 mM dATP, and 5U HotStarTaq (Qiagen #1032502). The Taq polymerase was activated at 95 °C for 15′. A-tailing reaction was then performed at 70 °C for 30′.

#### TA cloning

The A-tailed PCR product was cloned into the pGEM-T Easy Vector System (Promega #A1360) using the manufacturer’s protocol, with a 1:3 vector:insert molar ratio. A 10 µl reaction contained the appropriate amount of PCR product, 50 ng of vector, 1× reaction buffer, and 2U T4 ligase. For ligation, the reaction was incubated at 4 °C overnight. In total, 2 µl of the ligation reaction was added to a sterile 1.5 ml microcentrifuge tube containing 50 µl of competent *E. coli* cells (Stellar™ Competent Cells, Takara Bio #636763) on ice. The mix was incubated on ice for 30′ and then heat shocked for 45″ at 42 °C. After another 2′ incubation on ice, 500 µl of SOC medium (Takara Bio #ST0215) was added to the tubes, followed by 1 h incubation at 37 °C. As pGEM-T Easy Vectors allow blue/white selection, 100 µl of ligation reaction was plated on 100 µg/ml ampicillin (Sigma-Aldrich #A9518), 20 µg/µl X-gal (MELFORD #B71800), 100 µM IPTG (MELFORD #I56000) plates. These plates were incubated at 37 °C overnight.

#### Cloning

PCR products were digested with appropriate restriction enzymes by adding 1× restriction buffer (rCutSmart™ Buffer, New England Biolabs #B6004) and 20-50U of enzyme (NheI-New England Biolabs #R3131, KpnI-New England Biolabs #3142 or SacI- New England Biolabs #R3156), then incubated at 37 °C overnight followed by purification using QIAquick PCR Purification Kit (Qiagen #28106) or, in case of pGEM-T Easy Vectors, directly from an agarose gel using the MinElute Gel Extraction Kit (Qiagen #28606) according to the manufacturer’s protocol.

The fragments were cloned into three different pGL3 vectors (Promega), which contain the Firefly luciferase reporter gene. The three vectors are pGL3-Basic (containing no promoter or enhancer, Promega #E1751), pGL3-Promoter (containing a SV40 promoter element, Promega #E1761), and pGL3-Enhancer (containing a SV40 enhancer element, Promega #E1771). In total, 4 µg of these vectors were digested by NheI and KpnI restriction enzymes overnight at 37 °C. Digests were checked visually by gel electrophoresis to ensure complete digestion. After complete digestion, the enzyme was heat-inactivated at 65 °C for 20′. In all, 50 ng of the vector was ligated with an appropriate amount of insert to reach a 1:3 vector:insert molar ratio, using T4 ligase (New England Biolabs #M0202) and incubated at 16 °C overnight. Transformation was performed as described above.

#### Colony PCR

Colony PCR was performed in 0.2 ml microcentrifuge tubes or 96-well plates. Each 25 µl reaction contained 1× PCRBIO HS Taq Mix (PCR Biosystems #PB10.23-02), 10 µM of each insert-specific primer, and 5 µl of the water/bacteria mix as a template. The PCR program was 95 °C for 10′, followed by 25 cycles of 95 °C for 15″, 60 °C for 15″, 72 °C for 15″ and a 5′ extension cycle at 72 °C. The PCR products were visualized by agarose gel electrophoresis in TBE buffer and stained with SafeView.

#### Plasmid DNA minipreps

In all, 3–5 ml of LB medium containing 100 µg/ml ampicillin was inoculated with 200 µl LB medium containing half a positive bacterial colony and was grown at 37 °C with shaking at 0.425 × *g* overnight. DNA was prepared using the ZR Plasmid Mini/Midiprep-Classic Kit (Zymo Research #D4016/#D4200) according to the manufacturer’s protocol. The plasmid DNA was verified by sequencing using vector-specific primers described in Table S1.

#### Transfection

Before transfection of luciferase plasmids into mESCs, MEF feeder cells were depleted on 0.2% gelatin for 40′ at room temperature. In all, 1–2 × 10^4^ cells were seeded in 0.2% gelatin-coated wells of a 48-well plate and incubated for 6 h at 37 °C. For transfection, 500 ng of Firefly and 1 ng of *Renilla* plasmids were incubated together with 25 µl OptiMEM (Gibco #31985062) and 1 µl *Trans*IT-X2® (Mirus #MIR6000) for 20′ at room temperature. After that, the reaction was added dropwise to the mESCs. Fresh media was added on the next day. After 24 h, cells were taken for luciferase assays. To generate comparable results, each plate contained a positive control (pGL3-Control, Promega #E1741), a negative control (pRL-SV40 *Renilla* only, Promega #E2231), and an empty vector.

#### Dual-Luciferase® reporter assay system

The Dual-Luciferase® Reporter (DLR^TM^) Assay System (Promega #E1910) allowed for the measurement of the luminescence for Firefly and then *Renilla* sequentially with a single transfection reaction^46^. The DLR assay was performed 24 h after transfection. Cells were prepared by washing twice with PBS and then lysed with 65 µl/well 1× Passive Lysis buffer for 15′ on a rocking platform. To assess firefly luminescence, 10 μl of lysed cells were added to 50 μl Luciferase Assay Reagent (LARII) and put in a single channel TD20/20 Luminometer (Turner Designs) to measure the signal. The luminometer was programmed with a 2″ pre-read delay, followed by a 10″ measurement period. After Firefly, *Renilla* luminescence was read by adding 50 μl of Stop&Glo® substrate, which first quenches the Firefly reaction and then activates *Renilla*. Each construct was measured twice in triplicate wells. The firefly luciferase values were normalized to the *Renilla* values, and then each test construct was normalized to the positive control and then plotted as fold-change compared to the empty vector.

### mESCs cell culture

C57BL/6J × CAST/EiJ hybrid mESCs were cultured at 37 °C with 5% CO_2_, on plates coated with 0.2% gelatine on irradiated mouse embryonic fibroblasts (iMEFs, DR4), in standard ESCs medium: 500 ml DMEM (Gibco #31966-021) supplemented with 20% US certified FBS (Gibco #10270-106), 10 µg recombinant leukemia inhibitory factor (LIF, Merck #ESG1107), 0.1 mM beta-mercaptoethanol (Gibco #31350-010), penicillin/streptomycin 1 mM (Sigma-Aldrich #P0781), L-glutamine (Gibco #25030-024) and 1% nonessential amino acids (Gibco #11140-050).

### Generation of IG-TRE deletions in mESCs

#### Cloning

To establish mESCs deleted for the IG-TRE and for the SOX2, ZFP281, and PRDM14 binding sites at the IG-TRE, targeting vectors and CRISPR/Cas9 plasmids were cloned and co-transfected into mESCs. gRNAs were selected for minimal off-targets using CCTop- CRISPR/Cas9 target online predictor (https://crispr.cos.uni-heidelberg.de/, Stemmer 2015). sgRNA oligos were duplexed to double strands and phosphorylated by T4 PNK to allow subsequent cloning into Cas9 coding plasmids. Each 10 µl reaction contained 10 µM of each oligo (forward and reverse), 1 mM ATP (New England Biolabs #P0756), 1× reaction buffer, and 0.5U T4 PNK (New England Biolabs #M0201). The reaction was incubated at 37 °C for 30′, 95 °C for 5′, and then cooled down to 25 °C at 0.1 °C/s. The phosphorylated DNA products were cloned into the pSpCas9(BB)-2A-Puro plasmid (PX459) V2.0 (Addgene #62988), which expresses the gRNAs under the control of a U6 promoter, *Cas9* under the control of a CBh promoter, and includes ampicillin and puromycin resistance for the following selections. Cloning was done by BbsI restriction enzyme digest and T4 ligase-mediated ligation. Each 20 µl reaction contained 100 ng Cas9 vector, 2 µl of 1:200 diluted duplex oligo, 1XTango buffer (Thermo Fisher Scientific #BY5), 50 mM DTT, 5 mM ATP, 1U BbsI (New England Biolabs #R3539) and 0.5U T4 DNA ligase. The reaction was incubated in six cycles of 37 °C for 5′ followed by 21 °C for 5′. Plasmids were transformed into competent bacteria and prepped as described above. Sequences were checked by Sanger sequencing.

#### Transfection

In total, 5 × 10^5^ mESCs in 1 ml of media were seeded in 0.2% gelatin-coated wells of a six-well plate and incubated for 6 h at 37 °C. For transfection, 1.25 µg of each plasmid was incubated together with 250 µl OptiMEM, 7.5 µl *Trans*IT-X2®, and 100 µM ssODN for the binding site deletions, for 20″ at room temperature. After that, the reaction was added dropwise to the mESCs. After 24 h at 37 °C, MEFs were added to the stem cells again. To select positive clones, 48 h post transfection ESC media was supplemented with 1 µg/ml Puromycin (Thermo Fisher Scientific #A1113803). After 4 days, the selection media was replaced by normal ESC media again for recovery.

#### Genotyping—cell lines

Single colonies were picked into a 96-well plate and analyzed for deletions by PCR analysis and Sanger sequencing. For genotyping, 15 µl of cells were lysed in 35 µl lysis buffer containing 3.5 µg Proteinase K (Thermo Fisher Scientific #BP1700-100). The reaction was incubated for 2′ at 37 °C, 2 h at 55 °C, and finally for 10′ at 95 °C. To identify clones that carried the deletion, lysed cells were taken as a template for PCR. Each 20 µl PCR reaction contained 1× REDTaq® ReadyMix™ PCR Reaction Mix (Sigma-Aldrich #R2648), 10 µM of each primer, and 2 µl of the lysis mix as a template. The PCR program was 95 °C for 2′, followed by 35 cycles of 95 °C for 30″, 60 °C for 30″, 72 °C for 40″ and a 2′ extension cycle at 72 °C. The PCR products were visualized by agarose gel electrophoresis in TBE buffer and stained with SafeView.

sgRNAs, ssODN, homology arms, and genotyping primer sequences for generating the IG-TRE deletion and the SOX2, ZFP281, and PRDM14 binding site deletions within the IG-TRE are listed in Table S2.

### RNA and DNA extraction

Total RNA was isolated using Tri Reagent (Sigma-Aldrich #T9424) according to the manufacturer’s instructions, quantified by spectrophotometry, and quality controlled by gel electrophoresis to assess the integrity of the ribosomal RNA and absence of degradation products. Genomic DNA was isolated by phenol/chloroform. Alternatively, DNA was isolated by lysing cells in lysis buffer (0.1 M Tris buffer, 0.2 M NaCl, 0.005 M EDTA, 0.2% SDS) with 10 mg/ml Proteinease K at 55 °C, precipitated with Iso-Propanol, washed with 70% Ethanol, and resuspended in TE buffer.

### Pyrosequencing methylation analysis

The procedure and primers for this DNA methylation analysis were described previously^47–49^. In brief, 500 ng DNA was treated using the EZ DNA Methylation-Gold Kit (Zymo Research #D5005) in accordance with the manufacturer’s instructions. Bisulfite-treated DNA was eluted in 20 μl of elution buffer. Amplicons were generated in a 20 μl reaction volume containing 100 nM forward and reverse primers, 1.25 U of HotstarTaq DNA Polymerase (Qiagen #203203), 0.2 mM dNTPs, and 1 μl of bisulfite-treated DNA. PCR cycle conditions consisted of an initial activation step of 95 °C for 10′, followed by 40 cycles of 94 °C for 30″, specific annealing temperature for 30″, and extension at 72 °C for 30″, followed by a final extension at 72 °C for 5′. Pyrosequencing was carried out on a PSQ HS96 System using PyroMark Gold Q96 SQA Reagents (Qiagen #972812). The degree of methylation at CpG sites (without distinguishing between maternal and paternal alleles) was determined by the pyro-Q CpG software. Genomic coordinates of the assays, target SQ, number of targeted CpGs, and primer sequences are listed in Table S3.

### Reverse transcription and quantitative real-time PCR (qPCR)

RNA was treated with RNase-free DNaseI (Thermo Fisher Scientific #EN0521) prior to cDNA synthesis using RevertAid H Minus First Strand cDNA Synthesis Kit with oligo dT and random hexamer primers (Thermo Fisher Scientific #K1632). qPCR was performed with BrilliantII SYBR® Green QPCR Master Mix (Agilent Technologies #600882) in a 384-well plate on a QuantStudio™ 5 Real-Time PCR System (Applied Biosystems #A34322). Relative quantification of gene expression was normalized to the geometrical mean of GAPDH and β-Actin expression levels (primers are listed in Table S4) and calculated using the ΔΔCT method, plotted as 2^−ΔΔCT^. Pyrosequencing was carried out on a PSQ HS96 System using PyroMark Gold Q96 SQA Reagents (Qiagen #972812). SNP percentage was determined by the pyromark^TM^ MD 1.0 software.

### Chromatin immunoprecipitation (ChIP)–qPCR

ChIP was performed as previously described with some modifications^50,51^. In total, 5 × 10^6^ mESCs or 100 mg brain tissue were crosslinked in 1% formaldehyde for 10′ and subsequently quenched with Tris pH 8.0 (250 mM final) for 10′. The quenched cells were washed twice with ice-cold PBS supplemented with a protease inhibitor cocktail (EDTA-free cOmplete™, Sigma-Aldrich #11873580001), flash-frozen in liquid nitrogen, and stored at −80 °C. Crosslinked cells were thawed on ice and then lysed sequentially on ice and for 10′ at each step in each of the following buffers: LB1 (50 mM HEPES-KOH pH 7.4, 140 mM NaCl, 1 mM EDTA, 0.5 mM EGTA, 10% Glycerol, 0.5% NP-40, 0.25% Triton-X-100 and EDTA-free cOmplete™), LB2 (10 mM Tris-HCl pH 8.0, 200 mM NaCl, 1 mM EDTA, 0.5 mM EGTA and EDTA-free cOmplete™), and SDS-shearing lysis buffer (10 mM Tris-HCl pH 8, 1 mM EDTA, 0.15% SDS and EDTA-free cOmplete™). Nuclei from brain lysates were counted with Trypan Blue staining, and 5 × 10^6^ cells were taken forward for each ChIP. The lysates were sonicated (Bioruptor Plus, Diagenode) at 4 °C to generate DNA fragments of 100–500 bp (2 repetitions of 10 sonication cycles of 30 s on and 30 s off), and the sonicated lysates were subsequently clarified by centrifugation (12,100 ×* g* for 10′ at 4 °C). 10% of the mixture was taken as the input control. The sonicated lysate was topped up to 1 mL with LB3-500NaCl (20 mM Tris-HCl pH 7.5, 500 mM NaCl, 1 mM EDTA, 0.5 mM EGTA, 1% Triton-X-100, 0.1% Sodium deoxycholate, 0.1% SDS, and EDTA-free cOmplete™). This mixture was incubated overnight at 4 °C with Protein G Dynabeads (Invitrogen #10004D) that had been pre-blocked with 0.5% BSA and mixed with 5 µg Goat polyclonal SOX2 antibody (R&D Systems #AF2018) or Protein A Dynabeads (Invitrogen #10002D) mixed with 5 µg Rabbit polyclonal Zfp281/ZNF281 antibody (Abcam #ab101318). To remove non-specifically bound proteins, the ChIP beads were washed five times with RIPA Buffer (50 mM HEPES-KOH pH 7.4, 500 mM LiCl, 1 mM EDTA, 1% NP-40, and 0.7% Sodium deoxycholate) and once with TE Buffer (50 mM Tris-HCl pH 8.0, 10 mM EDTA) at 4 °C. The DNA-protein complex was eluted from the beads in Elution Buffer (50 mM Tris-HCl, pH 8.0, 10 mM EDTA, and 1% SDS) for 20′ at 65 °C, and reverse-crosslinked overnight at 65 °C. The eluted samples were then treated with 10 mg/ml RNase A (Sigma-Aldrich #R4875) and 20 mg/ml Proteinase K, and purified using the QIAquick PCR Purification Kit.

qPCR was performed with BrilliantII SYBR® Green qPCR Master Mix (Agilent Technologies #600882) in a 384-well plate on a QuantStudio™5 Real-Time PCR System (Applied Biosystems #A34322). The ChIP-qPCR fold enrichments were calculated according to the percent input method, where signals obtained from the ChIP are divided by signals obtained from the input sample. qPCR primers are listed in Table S5.

### CRISPR-zygote injections and generation of IG-TRE^∆550^ knockout mice

Deletion of 550 bp encompassing the distal region of a germline-derived intergenic differentially methylated region (IG-DMR, MGI:3051678) including the transcriptional regulation element (IG-TRE) was made by CRISPR/Cas9 gene editing by the Mary Lyon Centre at MRC Harwell as part of the Genome Editing Mice for Medicine (GEMM) program. Constructs were delivered by electroporation into 1-cell stage mouse embryos from a C57BL/6 J genetic background. Briefly, 90:10 deactivated Cas9:Cas9 protein, sgRNAs, and ssODNs were diluted and mixed in Electroporation buffer (EB; *Gibco* Opti-MEM I Reduced Serum Media (Thermo Fisher Scientific)) to the working concentrations of 650 ng/μl, 130 ng/μl total, and 400 ng/μl, respectively. Embryos were electroporated using the following conditions: 30 V, 3 ms pulse length, 100 ms pulse interval, 12 pulses. Electroporated embryos were re-implanted in CD1 pseudo-pregnant females. Host females were allowed to litter and rear F0 progeny.

For genotyping, genomic DNA was extracted from ear clip biopsies and amplified in a PCR reaction using the ThermoFisher SuperFi II PCR kit under the following conditions: 60 °C Annealing Temperature, 1.25′ Elongation time, wild-type product size: 1412 bp, Mutant product size: 862 bp. All amplicons were sent for Sanger sequencing to check for integration of the donor oligo sequence at the target site. No off-target activity was detected in the animals.

Copy counting of the donor sequence was carried out by ddPCR at the F1 stage to confirm that donor oligos were inserted once on target into the genome. A Taqman assay was used to copy count the donor sequence compared against a VIC-labeled reference assay for Dot1l. This ddPCR assay is specific to the wild-type allele, and only wild-type alleles are expected to be recognized by this assay. Therefore, wild-type controls are expected to call at two copies, and a single integration for a correct mutation is expected to call at 1 copy for F1 (heterozygote) animals. No additional donor integrations were detected in the animals taken forward to establish the colony.

sgRNAs, ssODN, off-target primers, TaqMan assay primers, TaqMan assay probes, and genotyping primer sequences for generating the IG-TRE^∆550^ deletion in mice are listed in Table S6.

### Mice lines

CAST/EiJ (RRID:IMSR_JAX:000928) and IG-TRE^∆550^ knockout mice were obtained from MRC Harwell Institute, and maintained under a 12 h light–dark cycle at 22 °C (+/− 2 °C) and 55% humidity (+/−10%). Mice were monitored for health and activity and were given ad libitum access to water and standard mouse chow. For pure and hybrid breeding experiments, mice were mated at 8–12 weeks of age. Male and female heterozygote F1 mice carrying the IG-TRE^∆550^ deletion were crossed with wild-type CAST/EiJ, wild-type C57BL/6J, or IG-CGI deletion partners, and F2 animals harboring the deletion allele were analyzed at different stages. All animal experiments were performed in accordance with the Animals (Scientific Procedures) Act 1986 Amendment Regulations 2012, following ethical review by the University of Cambridge Animal Welfare and Ethical Review Body. Animal experiments were approved by the UK Home Office project license #PP8193772. All efforts were made to minimize animal discomfort.

### Genotyping—mice

For genotyping, DNA was extracted from ear clips using PCR Biosystems PCRBIO Rapid extract PCR kit (PB10.24-40), following the manufacturer’s instructions. Genotyping of mouse strains and alleles was done by PCR; primers are listed in Tables S2 and S6.

### Tissue and embryo analysis

Animals harboring the IG-TRE^∆550^ deletion allele were analyzed at different ages. At 9 weeks, measurements of adult animal weight (g), brain weight (mg), liver weight (g), fat weight (mg), and animal length (cm) were recorded. Whole brains and livers were collected and homogenized for expression analysis. At embryonic day E15.5, measurements of embryo weight (mg), embryo crown-rump length (cm), and placenta weight (mg) were recorded, and brain, liver, and placenta were collected for expression analysis.

### Image analysis

Measurements of embryo length at E15.5 were carried out with ImageJ software and calculated as Feret’s Diameter.

### Statistical analysis

At least three biological replicates were performed for all experiments. Statistical differences were determined using a two-tailed unpaired Student’s *t* test. Data are shown as means with error bars representing the standard error (SEM). *P* values of less than 0.05 were considered significant. Degrees of freedom (t(df)), effect size statistics (${r}_{{Yl}}$), margin of error, and 95% confidence intervals are calculated for each *t* test and provided in the source data file.

### Reporting summary

Further information on research design is available in the Nature Portfolio Reporting Summary linked to this article.

## Supplementary information

## Source data

## Data Availability

All relevant data and details of resources can be found within the article and its supplementary information. Source data are provided with this paper.
