# Supplementary material for: Regulatory mechanisms of maternal imprinting at the murine Dlk1-Dio3 domain
Source: Nat Commun. 2026 Sep 2;17:9353. doi: 10.1038/s41467-026-77129-4 (PMC13538571; doi:10.1038/s41467-026-77129-4)
Supplement: Supplementary file 1 [file 41467_2026_77129_MOESM1_ESM.pdf]

Supplementary Information for:

Regulatory Mechanisms of Maternal Imprinting at the Murine Dlk1-Dio3 Domain

Ariella Weinberg-Shukron[1], Fran L. Dearden[1], Ana Moreno-Barriga[1], Lok Ting Nick New[1,2], Marco Müller[1,3], Rebecca C. Rancourt[1,4], Carol A. Edwards[1] and Anne C. Ferguson-Smith[1,*]

[1] Department of Genetics, University of Cambridge, Cambridge, CB2 3EH, United Kingdom
[2] Present address: Medical Research Council (MRC) Laboratory of Medical Sciences (LMS), London, W12 0HS, United Kingdom
[3] Present address: BioNTech SE, Mainz, 55118, Rhineland-Palatinate, Germany
[4] Present address: Institute of Clinical Research, Medical School Berlin (MSB), Berlin, Germany

* Corresponding author: afsmith@gen.cam.ac.uk

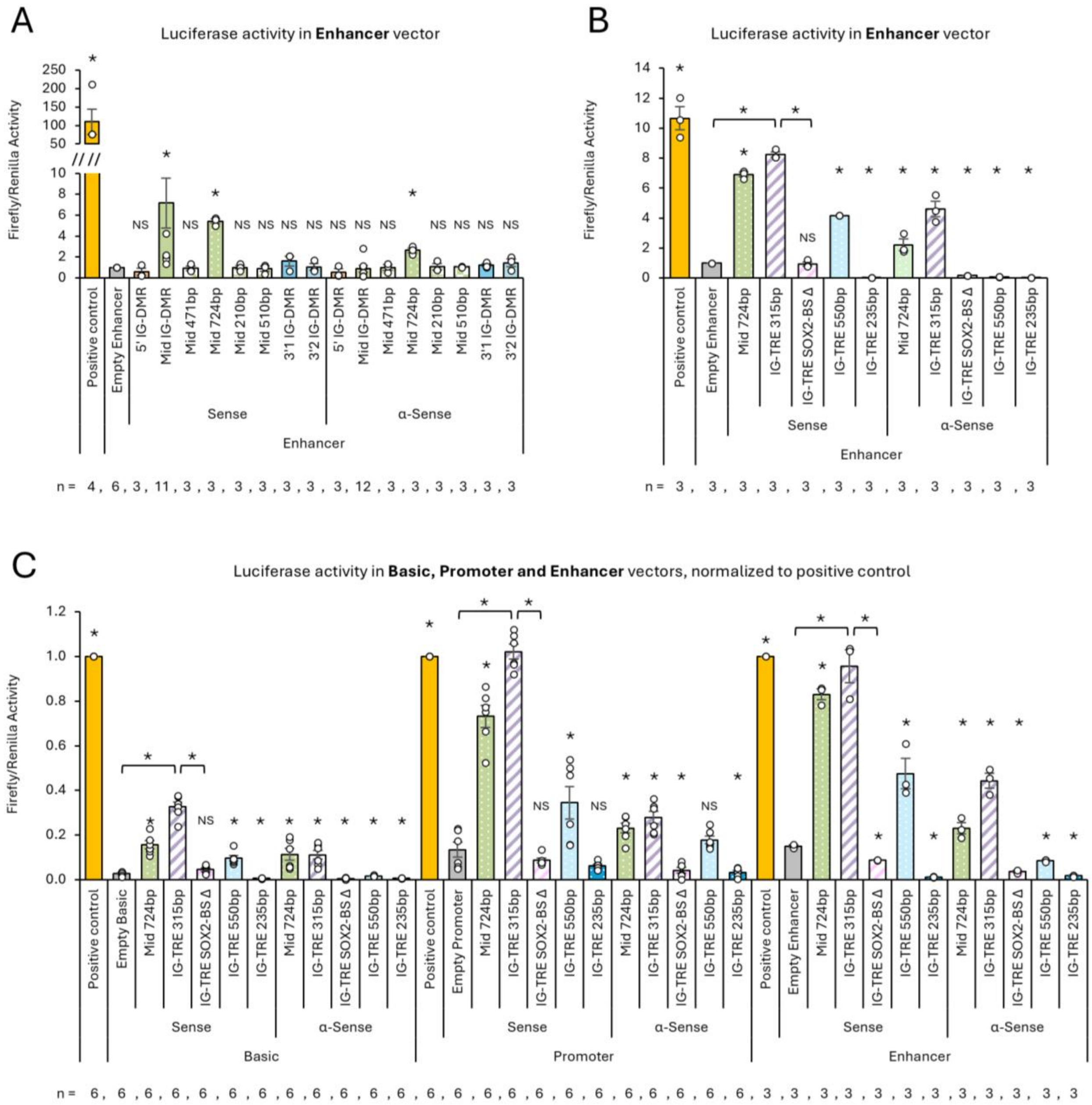

Figure S1- Enhancer and silencer activity detected at the IG-TRE. A. Graphical representation of the luciferase activity in the pGL3 Enhancer vector, normalized to the empty enhancer vector. For positive control, empty enhancer, 5' IG-DMR, Mid IG-DMR, Mid 471bp, Mid 742bp, Mid 210bp, Mid 510bp, 3'1 IG-DMR and 3'2 IG-DMR in sense and α-sense, n= 4, 6, 3, 11, 3, 3, 3, 3, 3, 3, 3, 12, 3, 3, 3, 3, 3 and 3 respectively, and significant p-values are: 0.004, 0.04, 8.8E^{-09}, 9.04E^{-06}. **B.** Graphical representation of the luciferase activity in the pGL3 Enhancer vector, normalized to the empty enhancer vector. For positive control, empty enhancer, Mid 742bp, IG-TRE 315bp, IG-TRE SOX2-BS Δ, IG-TRE 550bp and IG-TRE 235bp in sense and α-sense, n= 3, 3, 3, 3, 3, 3, 3, 3, 3, 3, 3 and 3 respectively, and significant p-values are: 0.0002, 2.3E^{-06}, 2.6E^{-06}, 5.9E^{-06}, 3.02E^{-13}, 9.9E^{-11}, 0.03, 0.002, 1.03E^{-08}, 5.7E^{-08}, 3.04E^{-09}. **C**. Graphical representation of the luciferase activity in the pGL3 Enhancer vector normalized to the positive control vector. For positive control, empty vector, Mid 742bp, IG-TRE 315bp, IG-TRE SOX2-BS Δ, IG-TRE 550bp and IG-TRE 235bp in basic and promoter in sense and α-sense, n= 6 for all

vectors, and for the enhancer n=3 for all vectors. significant p-values are: $2.6E^{-20}$, $2.9E^{-05}$, $6.8E^{-08}$, $1.96E^{-07}$, 0.0002, 0.0003, 0.009, 0.002, 0.0003, 0.0004, 0.02, 0.0003, $1.9E^{-10}$, $1.7E^{-06}$, $4E^{-09}$, $1.1E^{-10}$, 0.02, 0.03, 0.008, 0.02, $1.6E^{-05}$, 0.01, $9.3E^{-09}$, $1.01E^{-05}$, 0.0004, 0.0003, 0.0003, 0.009, $1.4E^{-05}$, 0.03, 0.001, $8.6E^{-05}$, 0.0003, 0.0004, $2.2E^{-05}$. The Mid 724bp fragment shows activity in sense orientation only, suggesting promoter activity. The Mid 315bp fragment shows activity similar to the 724bp fragment, suggesting enhancer activity. This activity is lost upon deletion of the SOX2-binding site from within the 315bp fragment. In parallel, the 235bp fragment, downstream of the 315bp region, shows silencing activity, indicating dual activity in a minimal region of 550bp in the IG-TRE with both enhancing and silencing regulation. Each construct was measured twice in triplicates wells. The firefly luciferase values were normalized to the *Renilla* values and then each test construct was normalized to the corresponding empty vector. Data are presented as mean values +/- SEM. NS- not significant. Asterisks indicate statistical significance in comparison to the empty vector using a two-tailed unpaired Student’s t-test. Source data are provided as a Source Data file.

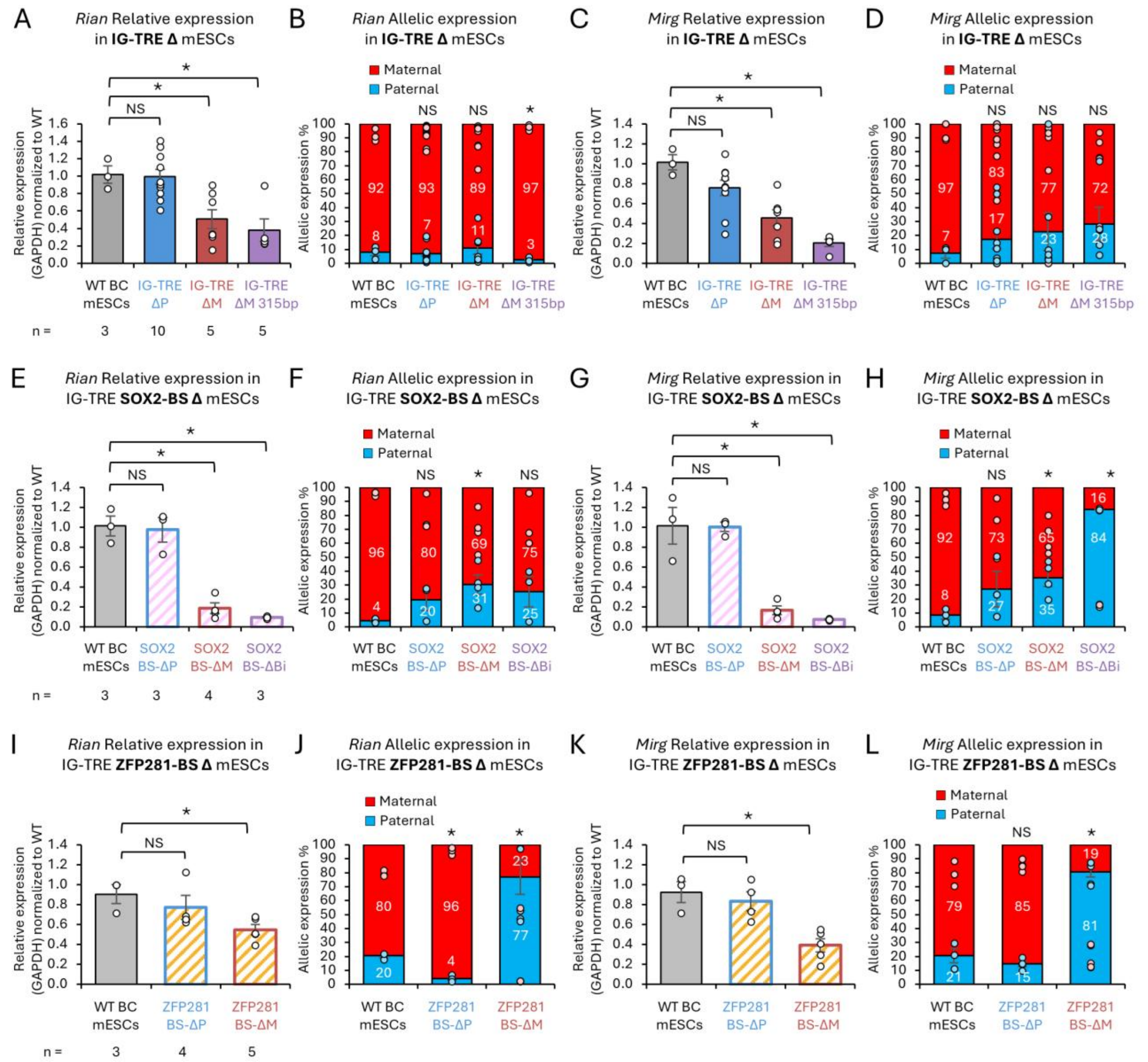

Figure S2- Deleting the entire maternal IG-TRE or the binding sites for SOX2 or ZFP281 within the IG-TRE *in vitro* results in dysregulation of the maternal transcripts *Rian* and *Mirg*. A+B. Graphical representation of *Rian* relative (A) and allelic (B) expression in mESC clones deleted for the entire IG-TRE (1.3kb) shows loss of *Rian* expression upon full maternal deletion or deleting even just 315bp within the IG-TRE ($p_{\Delta M}$=0.02, $p_{\Delta M\text{-}315bp}$=0.01 and 0.05). **C+D.** Graphical representation of *Mirg* relative (C) and allelic (D) expression in mESC clones deleted for the entire IG-TRE (1.3kb) shows loss of *Mirg* expression upon full maternal deletion or deleting even just 315bp within the IG-TRE ($p_{\Delta M}$=0.003, $p_{\Delta M\text{-}315bp}$=3.02E^{-05}). N_{WTBC}=3, $N_{IG\text{-}TRE\ \Delta P}$=10, $N_{IG\text{-}TRE\ \Delta M}$=5, $N_{IG\text{-}TRE\ \Delta M\ 315bp}$=5 independent biological replicates. **E+F.** Graphical representation of *Rian* relative (E) and allelic (F) expression in mESC clones deleted for the SOX2-binding site within the 315bp of the IG-TRE shows loss of *Rian* expression upon maternal or biallelic deletions ($p_{\Delta M}$=0.001 and 0.02, $p_{\Delta Bi}$=0.001). **G+H.** Graphical representation of *Mirg* relative (G) and allelic (H) expression in mESC clones deleted for the SOX2-binding site within the 315bp of the IG-TRE shows loss of *Mirg* expression upon maternal or biallelic deletions ($p_{\Delta M}$=0.003 and 0.01, $p_{\Delta Bi}$=0.007 and 1.05E^{-05}). N_{WTBC}=3, $N_{SOX2\ BS\text{-}\Delta P}$=3, $N_{SOX2\ BS\text{-}\Delta M}$=4, $N_{SOX2\ BS\text{-}\Delta Bi}$=3 independent

biological replicates. **I+J.** Graphical representation of *Rian* relative (I) and allelic (J) expression in mESC clones deleted for the ZFP281-binding site within the 315bp of the IG-TRE shows loss of *Rian* expression upon maternal deletion ($p_{\Delta M}$=0.01 and 0.02, $p_{\Delta P}$=0.0002). **K+L.** Graphical representation of *Mirg* relative (K) and allelic (L) expression in mESC clones deleted for the ZFP281-binding site within the 315bp of the IG-TRE shows loss of *Mirg* expression upon maternal deletion ($p_{\Delta M}$=0.004 and 0.0001). N_{WTBC}=3, $N_{ZFP281\ BS\text{-}\Delta P}$=4, $N_{ZFP281\ BS\text{-}\Delta M}$=5 independent biological replicates. Data are presented as mean values +/- SEM. NS- not significant. Asterisks indicate statistical significance in comparison to wildtype using a two-tailed unpaired Student's t-test. Source data are provided as a Source Data file.

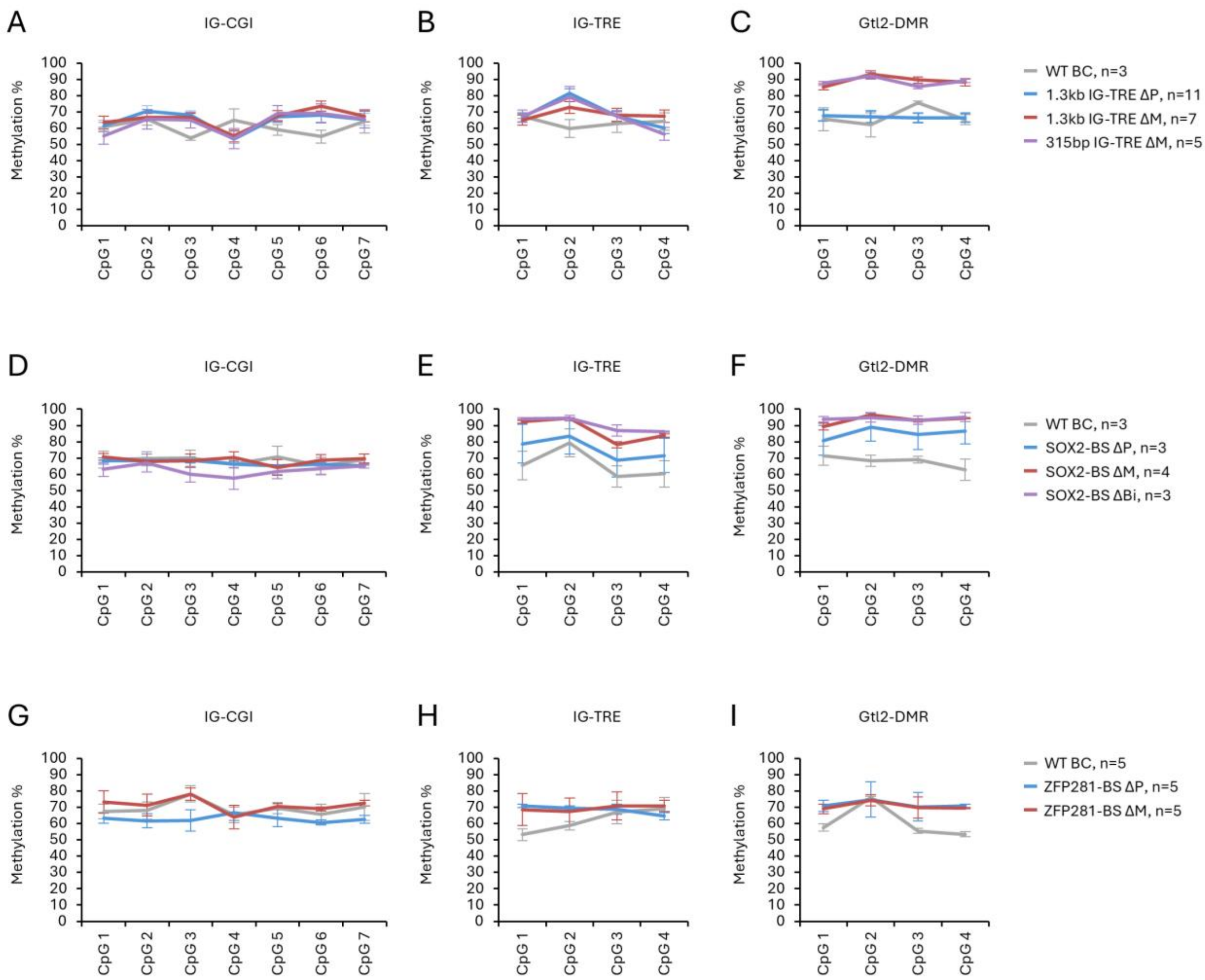

Figure S3- Deleting the entire IG-TRE, or the binding sites for either SOX2 or ZFP281 from within the IG-TRE *in vitro* results in increased methylation on the maternal chromosome. A-C. Graphical representation of the CpG methylation at three regulatory elements at the Dlk1-Dio3 domain, analyzed by bisulfite pyro sequencing, shows increased methylation at the Gtl2-DMR upon deletion of the entire 1.3kb IG-TRE and even just 315bp from the maternal chromosome. N_{WTBC}=3, $N_{IG\text{-}TRE\ \Delta P}$=11, $N_{IG\text{-}TRE\ \Delta M}$=7, $N_{IG\text{-}TRE\ \Delta M\ 315bp}$=5 independent biological replicates. **D-F.** Graphical representation of the CpG methylation at three regulatory elements at the Dlk1-Dio3 domain, analyzed by bisulfite pyro sequencing, shows increased methylation at the IG-TRE and the Gtl2-DMR upon deletion of the SOX2-binding site from within the IG-TRE on the maternal but not paternal chromosome. N_{WTBC}=3, $N_{SOX2\ BS\text{-}\Delta P}$=3, $N_{SOX2\ BS\text{-}\Delta M}$=4, $N_{SOX2\ BS\text{-}\Delta Bi}$=3. **G-I.** Graphical representation of the CpG methylation at three regulatory elements at the Dlk1-Dio3 domain, analyzed by bisulfite pyro sequencing, shows increased methylation at the Gtl2-DMR upon deletion of the ZFP281-binding site from within the IG-TRE on the maternal but not paternal chromosome. N_{WTBC}=5, $N_{ZFP281\ BS\text{-}\Delta P}$=5, $N_{ZFP281\ BS\text{-}\Delta M}$=5 independent biological replicates. Data are presented as mean values +/- SEM. Source data are provided as a Source Data file.

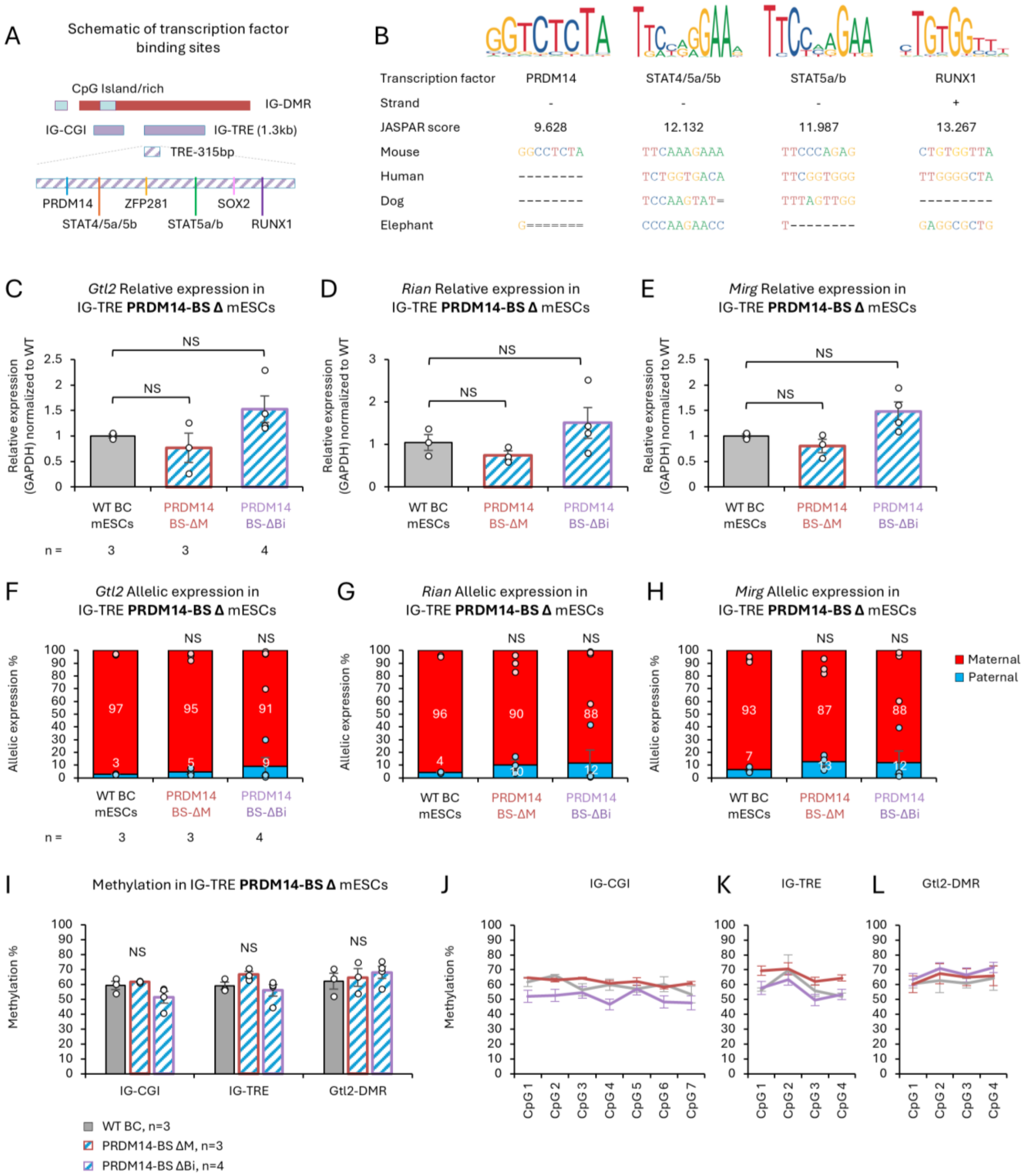

Figure S4- Deleting the PRDM14 binding site from within the 315bp of the IG-TRE *in vitro* does not change expression or methylation at the Dlk1-Dio3 domain. A. Schematic of predicted transcription factor binding sites within 315bp of the IG-TRE**. B.** Motifs and conservation of selected transcription factor binding sites, that although their JASPAR score was high, are not well conserved in placental mammals. **C-E.** Graphical representation of *Gtl2* (C), *Rian* (D) and *Mirg* (E) relative expression in mESC clones deleted for the PRDM14-binding site within the 315bp of the IG-TRE shows no change in expression upon maternal or biallelic deletions. **F-H.** Graphical representation of *Gtl2* (F), *Rian* (G) and *Mirg* (H) allelic expression in mESC clones deleted for the PRDM14-binding site within the 315bp of the IG-TRE shows no change in imprinting of these genes upon maternal or biallelic deletions. **I-L.** Graphical representation of the

methylation at three regulatory elements at the Dlk1-Dio3 domain, analyzed by bisulfite pyro sequencing, shows no change in methylation upon deletion of the PRDM14-binding site within the 315bp of the IG-TRE. N_{WTBC}=3, $N_{PRDM14\ BS\text{-}\Delta M}$=3, $N_{PRDM14\ BS\text{-}\Delta Bi}$=4 independent biological replicates. Data are presented as mean values +/- SEM. NS- not significant in comparison to wildtype using a two-tailed unpaired Student's t-test. Source data are provided as a Source Data file.

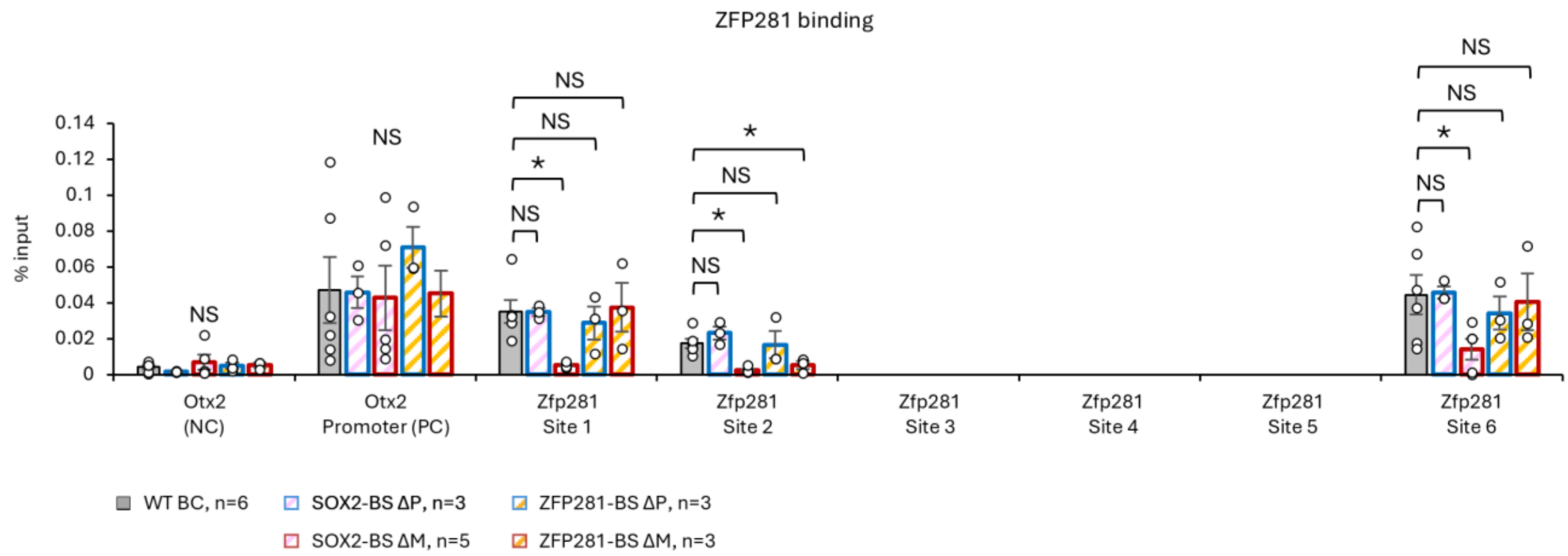

Figure S5- ZFP281 binding is dependent on SOX2 binding on the maternal chromosome. Graphical representation of ZFP281 binding at the IG-TRE, determined by ChIP-qPCR, shows depletion of ZFP281 binding at binding sites 1, 2 and 6, when the maternal SOX2-binding site is deleted from the 315bp region ($p_{SOX2\text{-}\Delta M}$=0.002, 0.001, 0.04). ZFP281 binding is also depleted at binding site 2 when this binding site is deleted from the maternal chromosome (as expected, $p_{ZFP281\text{-}\Delta M}$=0.02). No change in ZFP281 binding was observed upon deletion of the SOX2 or ZFP281 binding sites from the paternal chromosome. No binding was observed at predicted binding sites 3, 4 and 5 and no change in binding was observed at control sites at the *Otx2* gene. N_{WTBC}=6, $N_{SOX2\ BS\text{-}\Delta P}$=3, $N_{SOX2\ BS\text{-}\Delta M}$=5, $N_{ZFP281\ BS\text{-}\Delta P}$=3, $N_{ZFP281\ BS\text{-}\Delta M}$=3 independent biological replicates. Data are presented as mean values +/- SEM. NS- not significant. Asterisks indicate statistical significance in comparison to wildtype using a two-tailed unpaired Student's t-test. Source data are provided as a Source Data file.

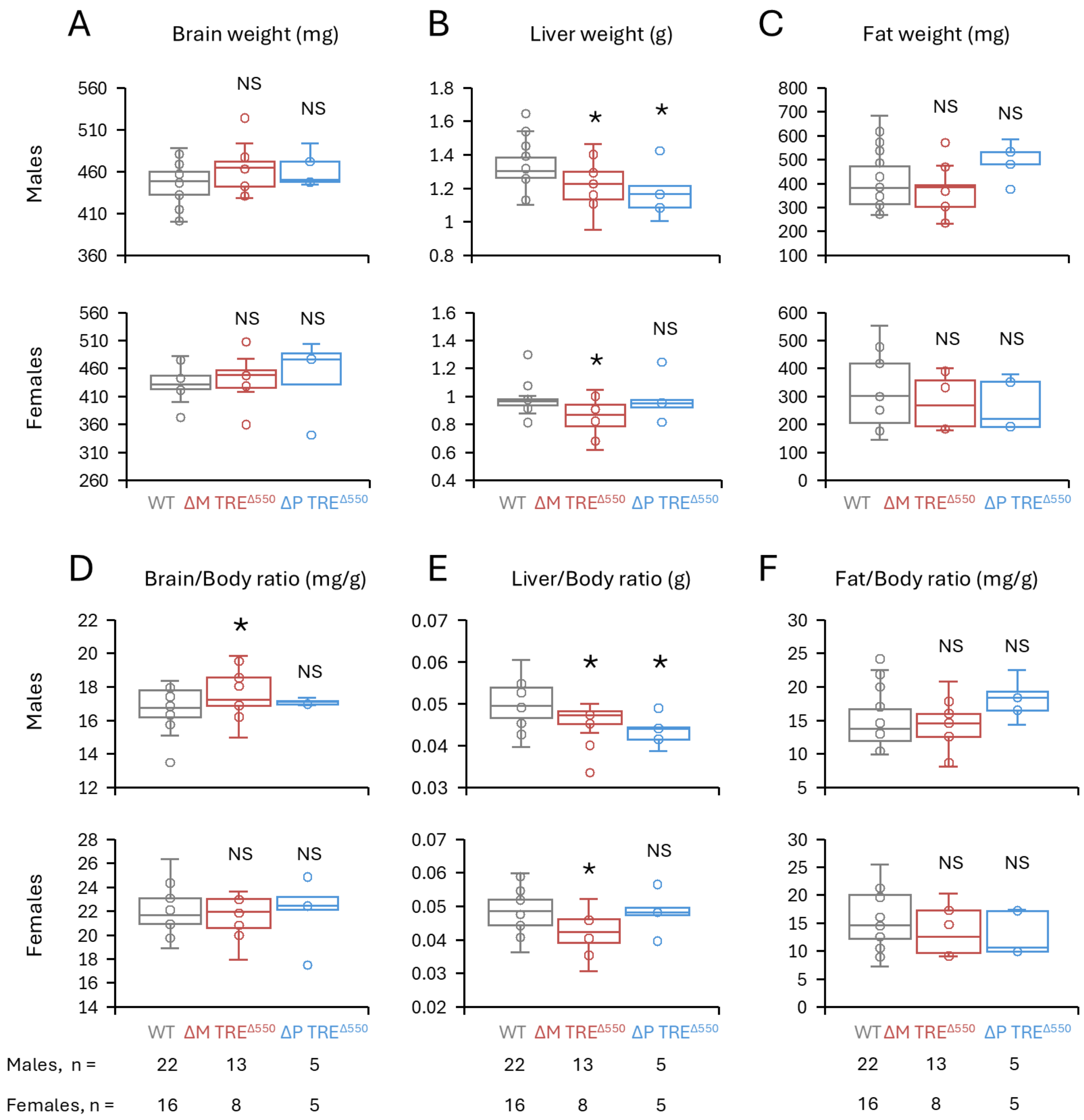

Figure S6- Deletion of the IG-TRE$^{\Delta550}$ *in vivo* is viable with a slight reduction in adult liver size. A. Box plot representation of brain weight (mg) of 9 week-old adult animals. For WT, ΔP TRE$^{\Delta550}$ and ΔM TRE$^{\Delta550}$ males: Min= 400.2, 444.3, 428.3; Q1= 432.8, 447.4, 442.4; Center line= 448.2, 450.4, 464.7; Q3= 459.9, 472.1, 471.9; Max= 488, 493.8, 523.8; n= 22, 5, 13. For WT, ΔP TRE$^{\Delta550}$ and ΔM TRE$^{\Delta550}$ females: Min= 372.1, 340.8, 359.7; Q1= 422.4, 430.9, 426; Center line= 431.4, 476.6, 448.2; Q3= 447.2, 486.6, 456.3; Max= 482, 503.7, 507.6; n= 16, 5, 8. **B.** Box plot representation of liver weight (g) of 9 week-old adult animals. For WT, ΔP TRE$^{\Delta550}$ and ΔM TRE$^{\Delta550}$ males: Min= 1.1, 1, 1; Q1= 1.3, 1.1, 1.1; Center line= 1.3, 1.2, 1.2; Q3= 1.4, 1.2, 1.3; Max= 1.6, 1.4, 1.5; n= 22, 5, 13; p=0.05, 0.05. For WT, ΔP TRE$^{\Delta550}$ and ΔM TRE$^{\Delta550}$ females: Min= 0.8, 0.8, 0.6; Q1= 0.9, 0.9, 0.8; Center line= 1, 1, 0.9; Q3= 1, 1, 0.9; Max= 1.3, 1.2, 1; n= 16, 5, 8; p=0.04. **C.** Box plot representation of fat weight (mg) of 9 week-old adult animals. For WT, ΔP TRE$^{\Delta550}$ and ΔM TRE$^{\Delta550}$ males: Min= 269.1, 376.5, 231.4; Q1= 313.7, 480.2, 303.7; Center line= 381.8, 480.2, 385.5; Q3= 471, 532.1, 394.2; Max= 684.5, 583.9, 570.9; n= 22, 5, 13. For WT, ΔP TRE$^{\Delta550}$

and ΔM TRE$^{\Delta550}$ females: Min= 143.9, 189.7, 177.7; Q1= 205.8, 191.8, 194; Center line= 301.7, 218.9, 267.2; Q3= 418.3, 351.9, 357.7; Max= 554, 378.3, 401.7; n= 16, 5, 8. **D.** Box plot representation of brain/body weight ratio (mg/g) of 9 week-old adult animals. For WT, ΔP TRE$^{\Delta550}$ and ΔM TRE$^{\Delta550}$ males: Min= 13.5, 16.9, 15; Q1= 16.2, 17, 16.9; Center line= 16.7, 17.1, 17.2; Q3= 17.8, 17.1, 18.6; Max= 18.4, 17.3, 19.8; n= 22, 5, 13; p=0.09. For WT, ΔP TRE$^{\Delta550}$ and ΔM TRE$^{\Delta550}$ females: Min= 18.9, 17.5, 17.9; Q1= 20.9, 22.1, 20.6; Center line= 21.7, 22.4, 21.9; Q3= 23.1, 23.2, 23; Max= 26.3, 24.9, 23.6; n= 16, 5, 8. **E.** Box plot representation of liver/body weight ratio (g) of 9 week-old adult animals. For WT, ΔP TRE$^{\Delta550}$ and ΔM TRE$^{\Delta550}$ males: Min= 0.04, 0.039, 0.034; Q1= 0.047, 0.042, 0.045; Center line= 0.049, 0.044, 0.047; Q3= 0.054, 0.044, 0.048; Max= 0.061, 0.049, 0.05; n= 22, 5, 13; p=0.01, 0.02. For WT, ΔP TRE$^{\Delta550}$ and ΔM TRE$^{\Delta550}$ females: Min= 0.036, 0.04, 0.031; Q1= 0.044, 0.047, 0.039; Center line= 0.049, 0.048, 0.042; Q3= 0.052, 0.05, 0.046; Max= 0.06, 0.057, 0.052; n= 16, 5, 8; p=0.03. **F.** Box plot representation of fat/body weight ratio (mg/g) of 9 week-old adult animals. For WT, ΔP TRE$^{\Delta550}$ and ΔM TRE$^{\Delta550}$ males: Min= 9.9, 14.3, 8.1; Q1= 12, 16.5, 12.6; Center line= 13.8, 18.4, 14.6; Q3= 16.7, 19.3, 16; Max= 24.4, 22.5, 20.8; n= 22, 5, 13. For WT, ΔP TRE$^{\Delta550}$ and ΔM TRE$^{\Delta550}$ females: Min= 7.3, 9.9, 9; Q1= 12.1, 9.9, 9.7; Center line= 14.6, 10.7, 12.6; Q3= 20, 17.2, 17.3; Max= 25.5, 17.4, 20.2; n= 16, 5, 8. NS- not significant. Asterisks indicate statistical significance in comparison to wildtype using a two-tailed unpaired Student's t-test. Source data are provided as a Source Data file.

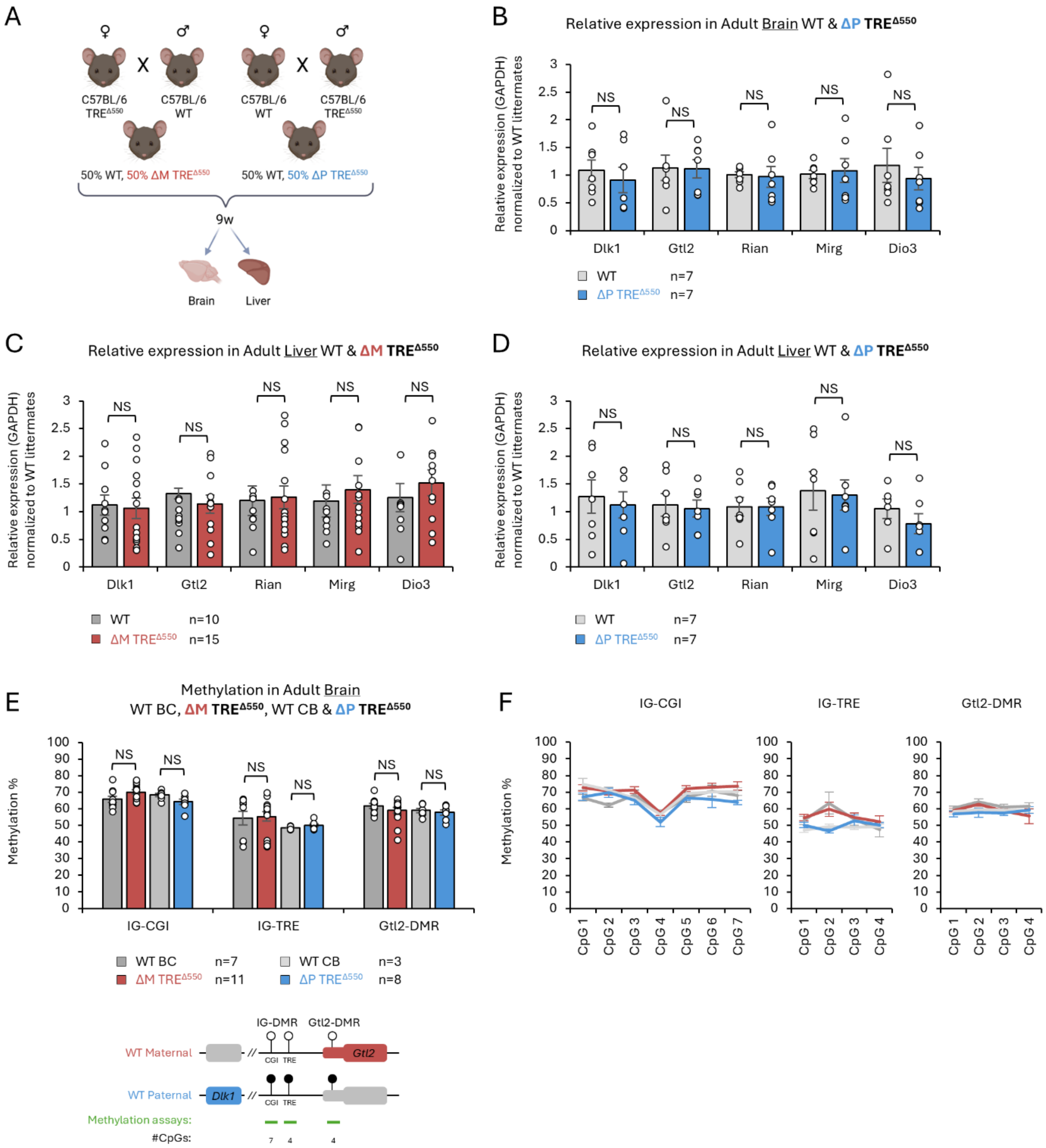

Figure S7- Deletion of the IG-TRE$^{\Delta550}$ does not change expression in adult liver, nor does expression or methylation change by paternal deletion in brains. A. Breeding strategy of the IG-TRE 550bp deletion animals crossed with C57BL/6 partners to produce maternal or paternal inheritance of the IG-TRE$^{\Delta550}$ deletion. In each litter, 50% of the pups are wildtype littermates (pure C57BL/6) and kept as controls. Tissues were collected for expression at 9 weeks. Created in BioRender. Edwards, C. (2026) https://BioRender.com/p2n1s4s. **B.** Graphical representation of the relative expression of the Dlk1-Dio3 genes, as measured by qPCR, in adult brains of paternal IG-TRE$^{\Delta550}$ and wildtype littermates. No change in expression is observed. **C.** Graphical representation of the relative expression of the Dlk1-Dio3 genes, as measured by qPCR, in adult livers of maternal IG-TRE$^{\Delta550}$ and wildtype littermates. No change in expression is observed. **D.** Graphical representation of the relative expression of the Dlk1-Dio3 genes, as measured by qPCR, in adult livers of paternal IG-TRE$^{\Delta550}$ and wildtype littermates. No change in expression is

observed. **E+F.** Schematic and graphical representation of the methylation at three regulatory elements at the Dlk1-Dio3 domain, as measured by bisulfite pyro sequencing, in adult brains of all genotype groups. No change in methylation is observed upon deletion of the IG-TRE$^{\Delta 550}$ from either chromosome. N_{WT}=7, $N_{\Delta P\text{-}TRE}{}^{\Delta 550}$=7 biologically independent adult brains, and N_{WT}=10, $N_{\Delta M\text{-}TRE}{}^{\Delta 550}$=15, N_{WT}=7, $N_{\Delta P\text{-}TRE}{}^{\Delta 550}$=7 biologically independent adult livers were analyzed for gene expression; N_{WTBC}=7, $N_{\Delta M\text{-}TRE}{}^{\Delta 550}$=11, N_{WTCB}=3, $N_{\Delta P\text{-}TRE}{}^{\Delta 550}$=8, biologically independent adult brains were analyzed for methylation. Data are presented as mean values +/- SEM. NS- not significant in comparison to wildtype using a two-tailed unpaired Student's t-test. Source data are provided as a Source Data file.

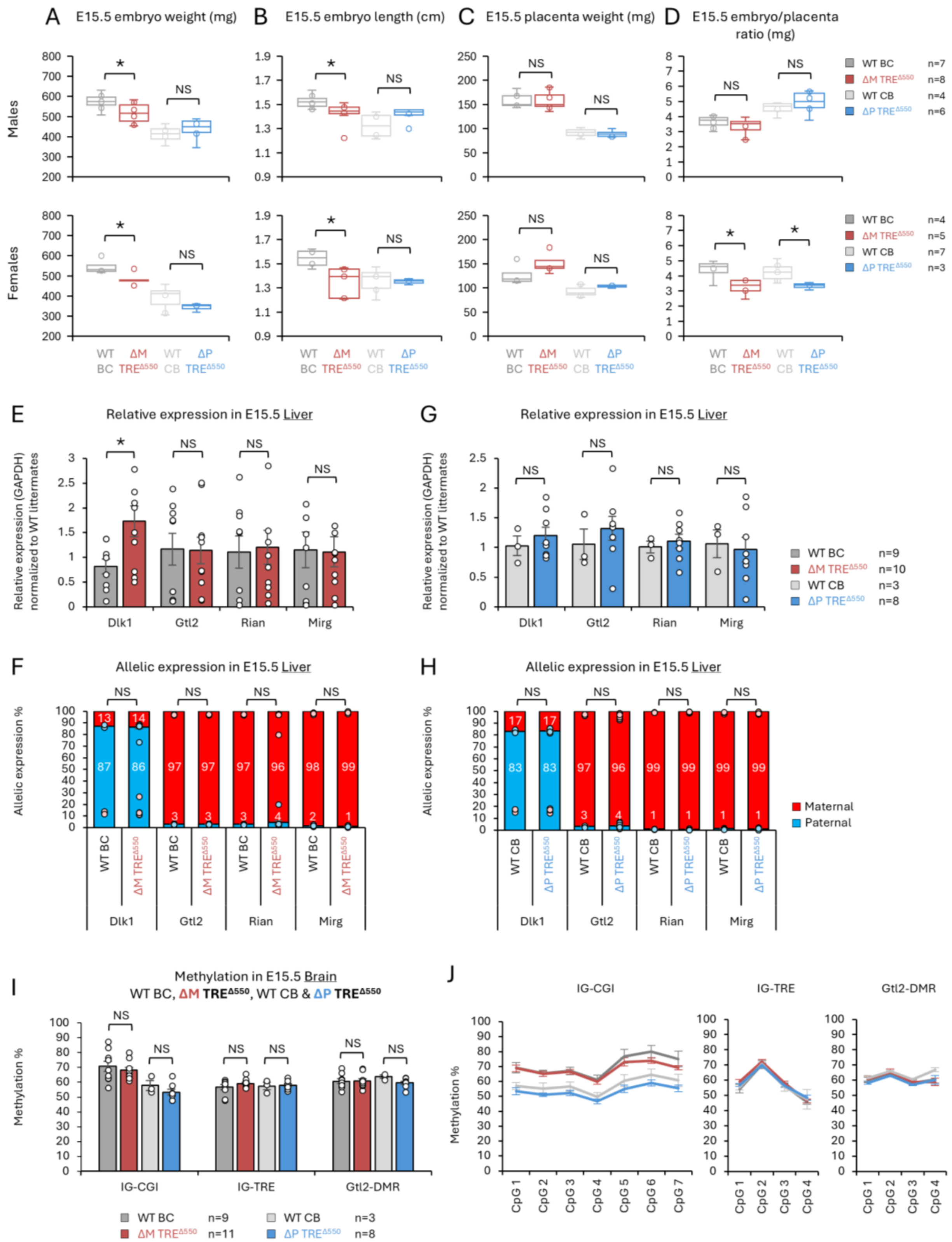

Figure S8- Deletion of the maternal, but not paternal, IG-TRE$^{\Delta550}$ *in vivo* results in increased maternal *Dlk1* expression in embryonic livers, without changing the methylation at the Dlk1-Dio3 domain. A. Box plot representation of E15.5 embryo weight (mg) by sex. For WTBC, ΔM TRE$^{\Delta550}$, WTCB and ΔP TRE$^{\Delta550}$ males: Min= 506.8, 453.2, 353.8, 346.6; Q1= 558.1, 477.7, 389.1, 420.2; Center line= 575.5, 517.2, 415.7, 448.3; Q3= 596.3, 557, 439, 478.2; Max= 630, 583, 464.5, 488; n= 7, 8, 4, 6; p=0.03. For WTBC, ΔM TRE$^{\Delta550}$, WTCB and ΔP TRE$^{\Delta550}$ females:

Min= 517.2, 451.8, 307.2, 320.7; Q1= 522.9, 475.2, 358.9, 336.5; Center line= 530.5, 476, 411.7, 352.2; Q3= 552.1, 479.8, 426.4, 356.1; Max= 599.7, 535.1, 457, 359.9; n= 4, 5, 7, 3; p=0.03. **B.** Box plot representation of E15.5 embryo crown-rump length (cm) by sex. For WTBC, ∆M TRE$^{\Delta550}$, WTCB and ∆P TRE$^{\Delta550}$ males: Min= 1.46, 1.22, 1.22, 1.3; Q1= 1.49, 1.42, 1.24, 1.41; Center line= 1.52, 1.45, 1.32, 1.44; Q3= 1.55, 1.48, 1.41, 1.46; Max= 1.62, 1.52, 1.44, 1.46; n= 7, 8, 4, 6; p=0.03. For WTBC, ∆M TRE$^{\Delta550}$, WTCB and ∆P TRE$^{\Delta550}$ females: Min= 1.21, 1.2, 1.33; Q1= 1.49, 1.21, 1.3, 1.34; Center line= 1.55, 1.4, 1.39, 1.36; Q3= 1.6, 1.46, 1.43, 1.37; Max= 1.62, 1.47, 1.48, 1.38; n= 4, 5, 7, 3; p=0.03. **C.** Box plot representation of E15.5 placenta weight (mg) by sex. For WTBC, ∆M TRE$^{\Delta550}$, WTCB and ∆P TRE$^{\Delta550}$ males: Min= 143.7, 135.8, 78.7, 82.1; Q1= 148.3, 146.3, 85.5, 83.8; Center line= 149.6, 150.2, 91.8, 89; Q3= 168.1, 169.6, 97.6, 92.1; Max= 183.1, 185.6, 102.5, 100.7; n= 7, 8, 4, 6. For WTBC, ∆M TRE$^{\Delta550}$, WTCB and ∆P TRE$^{\Delta550}$ females: Min= 110.9, 129.9, 80.3, 99.1; Q1= 114.1, 140.9, 86.3, 101.9; Center line= 118.1, 144.5, 89.8, 104.6; Q3= 130.6, 157.3, 99.3, 105.3; Max= 159.5, 183.6, 107.8, 106; n= 4, 5, 7, 3. **D.** Box plot representation of E15.5 e embryo/placenta weight ratio (mg) by sex. For WTBC, ∆M TRE$^{\Delta550}$, WTCB and ∆P TRE$^{\Delta550}$ males: Min= 3, 2.4, 3.9, 3.8; Q1= 3.4, 3.1, 4.3, 4.6; Center line= 3.8, 3.5, 4.7, 5; Q3= 3.9, 3.7, 4.9, 5.5; Max= 4.1, 4, 4.9, 5.7; n= 7, 8, 4, 6. For WTBC, ∆M TRE$^{\Delta550}$, WTCB and ∆P TRE$^{\Delta550}$ females: Min= 3.4, 2.5, 3.5, 3.1; Q1= 4.2, 3, 3.8, 3.2; Center line= 4.6, 3.4, 4.2, 3.4; Q3= 4.8, 3.7, 4.6, 3.5; Max= 5, 3.7, 5.1, 3.6; n= 4, 5, 7, 3; $p_{\Delta M}$=0.03, $p_{\Delta P}$=0.03. **E.** Graphical representation of the relative expression of the Dlk1-Dio3 genes, as measured by qPCR, in E15.5 livers of maternal IG-TRE$^{\Delta550}$ and BxC wildtype littermates (p_{Dlk1}=0.02). **F.** Graphical representation of the allelic expression of the Dlk1-Dio3 genes, as measured by pyro SNP analysis, in E15.5 livers of maternal IG-TRE$^{\Delta550}$ and BxC wildtype littermates. Expression analysis shows that *Dlk1* expression is increased in maternal IG-TRE$^{\Delta550}$ E15.5 livers, similar to that observed in E15.5 brains. Other gene expression is unchanged. **G.** Graphical representation of the relative expression of the Dlk1-Dio3 genes, as measured by qPCR, in E15.5 livers of paternal IG-TRE$^{\Delta550}$ and CxB wildtype littermates. **H.** Graphical representation of the allelic expression of the Dlk1-Dio3 genes, as measured by pyro SNP analysis, in E15.5 livers of paternal IG-TRE$^{\Delta550}$ and CxB wildtype littermates. No change in gene expression is observed. **I+J.** Graphical representation of the methylation at three regulatory elements at the Dlk1-Dio3 domain, as measured by bisulfite pyro sequencing, in E15.5 brains of all genotype groups. No change in methylation is observed upon deletion of the IG-TRE$^{\Delta550}$ from either chromosome. N_{WTBC}=9, $N_{\Delta M\text{-}TRE}{}^{\Delta550}$=10, N_{WTCB}=3 and $N_{\Delta P\text{-}TRE}{}^{\Delta550}$=8 biologically independent E15.5 livers were analyzed for gene expression; and N_{WTBC}=9, $N_{\Delta M\text{-}TRE}{}^{\Delta550}$=11, N_{WTCB}=3 and $N_{\Delta P\text{-}TRE}{}^{\Delta550}$=8 biologically independent E15.5 brains were analyzed for methylation. Data are presented as mean values +/- SEM. NS- not significant. Asterisks indicate statistical significance in comparison to wildtype using a two-tailed unpaired Student's t-test. Source data are provided as a Source Data file.

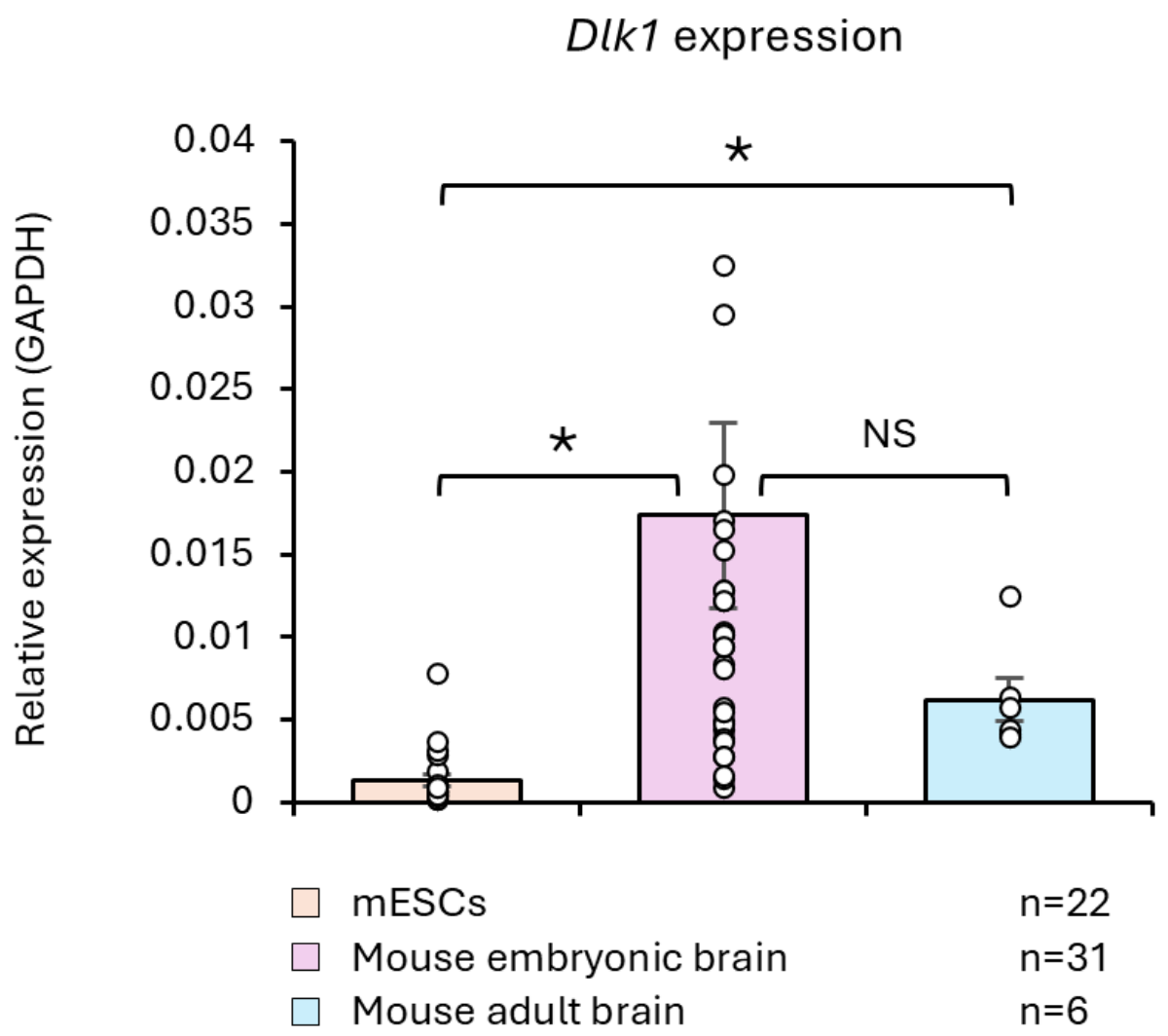

Figure S9- *Dlk1* expression is highest in mouse embryonic brains and decreases in the adult brain. *Dlk1* expression is 2.8 times higher in mouse embryonic brains than in adult brains, and 12.9 time higher than in mESCs ($p_{mESCs:embryonic\ brain}=0.02$, $p_{mESCs:adult\ brain}=3.2E^{-05}$)**.** $N_{mESCs}=22$, $N_{Mouse\ embryonic\ brain}=31$ and $N_{Mouse\ adult\ brain}=6$ biologically independent samples were analyzed for *Dlk1* gene expression. Data are presented as mean values +/- SEM. NS- not significant. Asterisks indicate statistical significance in comparison to wildtype using a two-tailed unpaired Student's t-test. Source data are provided as a Source Data file.

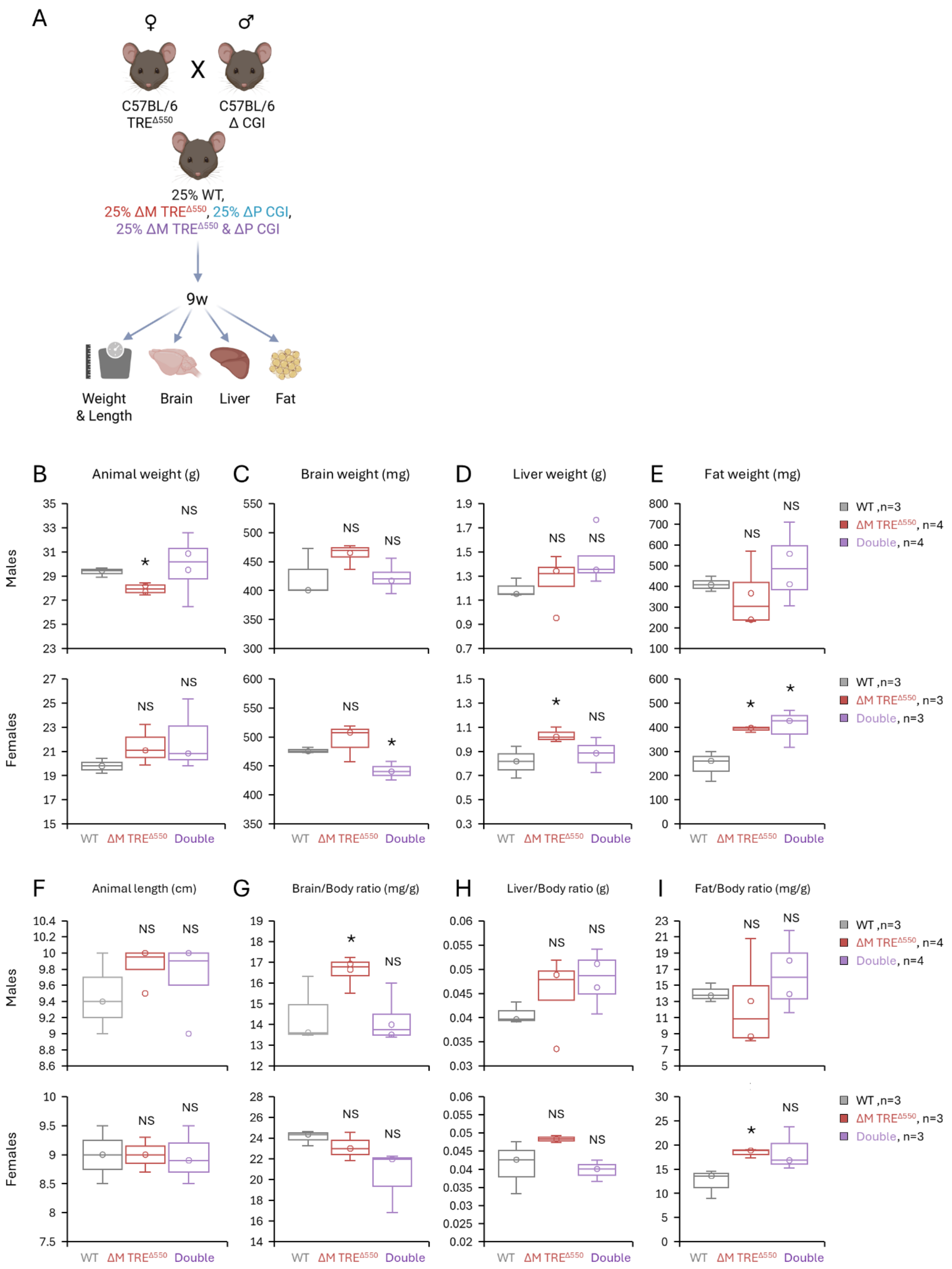

Figure S10- ΔP-IG-CGI and ΔM-IG-TRE$^{\Delta 550}$ double deletion pups are normal in size and rescue the lethality of the paternal IG-CGI deletion *in vivo*. A. Schematic representation of the predicted outcome of crossing ΔP-IG-CGI and ΔM-IG-TRE$^{\Delta 550}$ mouse strains. In each litter, 25% of the pups are wildtype littermates (pure C57BL/6) and kept as controls. Tissues were collected for expression at 9 weeks. Created in BioRender. Edwards, C. (2026) https://BioRender.com/4lav8lq. **B.** Box plot representation of total body weight (g) of 9 week-old

adult animals by sex. For WT, ΔM TRE$^{\Delta550}$ and double deletion males: Min= 28.9, 27.5, 26.5; Q1= 29.2, 27.6, 28.7; Center line= 29.5, 27.9, 30.2; Q3= 29.6, 28.3, 31.3; Max= 29.7, 28.4, 32.6; n= 3, 4, 4; p=0.01. For WT, ΔM TRE$^{\Delta550}$ and double deletion females: Min= 19.2, 19.9, 19.8; Q1= 19.5, 20.5, 20.3; Center line= 19.8, 21.1, 20.8; Q3= 20.1, 22.2, 23.1; Max= 20.4, 23.3, 25.4; n= 3, 3, 3. **C.** Box plot representation of brain weight (mg) of 9 week-old adult animals. For WT, ΔM TRE$^{\Delta550}$ and double deletion males: Min= 400.2, 437, 395.1; Q1= 400.5, 457.8, 411.5; Center line= 400.8, 469, 420.2; Q3= 436.5, 474.3, 431.6; Max= 472.1, 477.1, 456.1; n= 3, 4, 4. For WT, ΔM TRE$^{\Delta550}$ and double deletion females: Min= 472.8, 457.5, 426.1; Q1= 474.1, 482.6, 433.4; Center line= 475.3, 507.6, 440.7; Q3= 478.7, 513, 449.2; Max= 482, 518.3, 457.6; n= 3, 3, 3; p=0.02. **D.** Box plot representation of liver weight (g) of 9 week-old adult animals. For WT, ΔM TRE$^{\Delta550}$ and double deletion males: Min= 1.1, 1, 1.3; Q1= 1.1, 1.2, 1.3; Center line= 1.2, 1.3, 1.4; Q3= 1.2, 1.4, 1.5; Max= 1.3, 1.5, 1.8; n= 3, 4, 4. For WT, ΔM TRE$^{\Delta550}$ and double deletion females: Min= 0.7, 1, 0.7; Q1= 0.7, 1, 0.8; Center line= 0.8, 1, 0.9; Q3= 0.9, 1.1, 1; Max= 0.9, 1.1, 1; n= 3, 3, 3; p=0.06. **E.** Box plot representation of fat weight (mg) of 9 week-old adult animals. For WT, ΔM TRE$^{\Delta550}$ and double deletion males: Min= 376.2, 231.4, 307.7; Q1= 392, 237.8, 384.9; Center line= 407.7, 303.7, 484.1; Q3= 428.7, 418.3, 595.7; Max= 449.7, 570.9, 709.8; n= 3, 4, 4. For WT, ΔM TRE$^{\Delta550}$ and double deletion females: Min= 176.5, 379.7, 317.7; Q1= 218.8, 388.7, 372.1; Center line= 261, 397.7, 426.5; Q3= 280, 399.7, 448.5; Max= 299, 401.7, 470.5; n= 3, 3, 3; $p_{\Delta M\text{-}TRE}$=0.02, p_{Double}=0.05. **F.** Box plot representation of body length (cm) of 9 week-old adult animals. For WT, ΔM TRE$^{\Delta550}$ and double deletion males: Min= 9, 9.5, 9; Q1= 9.2, 9.8, 9.6; Center line= 9.4, 10, 9.9; Q3= 9.7, 10, 10; Max= 10, 10, 10; n= 3, 4, 4. For WT, ΔM TRE$^{\Delta550}$ and double deletion females: Min= 8.5, 8.7, 8.5; Q1= 8.8, 8.9, 8.7; Center line= 9, 9, 8.9; Q3= 9.3, 9.2, 9.2; Max= 9.5, 9.3, 9.5; n= 3, 3, 3. **G.** Box plot representation of brain/body weight ratio (mg/g) of 9 week-old adult animals. For WT, ΔM TRE$^{\Delta550}$ and double deletion males: Min= 13.5, 15.5, 13.4; Q1= 13.5, 16.4, 13.5; Center line= 13.6, 16.8, 13.8; Q3= 15, 17, 14.5; Max= 16.3, 17.2, 16; n= 3, 4, 4; p=0.07. For WT, ΔM TRE$^{\Delta550}$ and double deletion females: Min= 23.3, 21.8, 16.8; Q1= 23.8, 22.4, 19.4; Center line= 24.3, 23, 22; Q3= 24.5, 23.8, 22.1; Max= 24.6, 24.6, 22.2; n= 3, 3, 3. **H.** Box plot representation of liver/body weight ratio (g) of 9 week-old adult animals. For WT, ΔM TRE$^{\Delta550}$ and double deletion males: Min= 0.039, 0.034, 0.041; Q1= 0.039, 0.044, 0.045; Center line= 0.04, 0.048, 0.049; Q3= 0.041, 0.05, 0.052; Max= 0.043, 0.052, 0.054; n= 3, 4, 4. For WT, ΔM TRE$^{\Delta550}$ and double deletion females: Min= 0.033, 0.047, 0.037; Q1= 0.038, 0.048, 0.038; Center line= 0.043, 0.048, 0.04; Q3= 0.045, 0.049, 0.041; Max= 0.048, 0.049, 0.042; n= 3, 3, 3. **I.** Box plot representation of fat/body weight ratio (mg/g) of 9 week-old adult animals. For WT, ΔM TRE$^{\Delta550}$ and double deletion males: Min= 13, 8.1, 11.6; Q1= 13.4, 8.5, 13.3; Center line= 13.7, 10.8, 16; Q3= 14.5, 15, 19; Max= 15.3, 20.8, 21.8; n= 3, 4, 4. For WT, ΔM TRE$^{\Delta550}$ and double deletion females: Min= 8.9, 17.3, 15.2; Q1= 11.3, 18.1, 16; Center line= 13.6, 18.8, 16.8; Q3= 14.1, 19, 20.3; Max= 14.6, 19.1, 23.8; n= 3, 3, 3; p=0.03. NS- not significant. Asterisks indicate statistical significance in comparison to wildtype using a two-tailed unpaired Student's t-test. Source data are provided as a Source Data file.

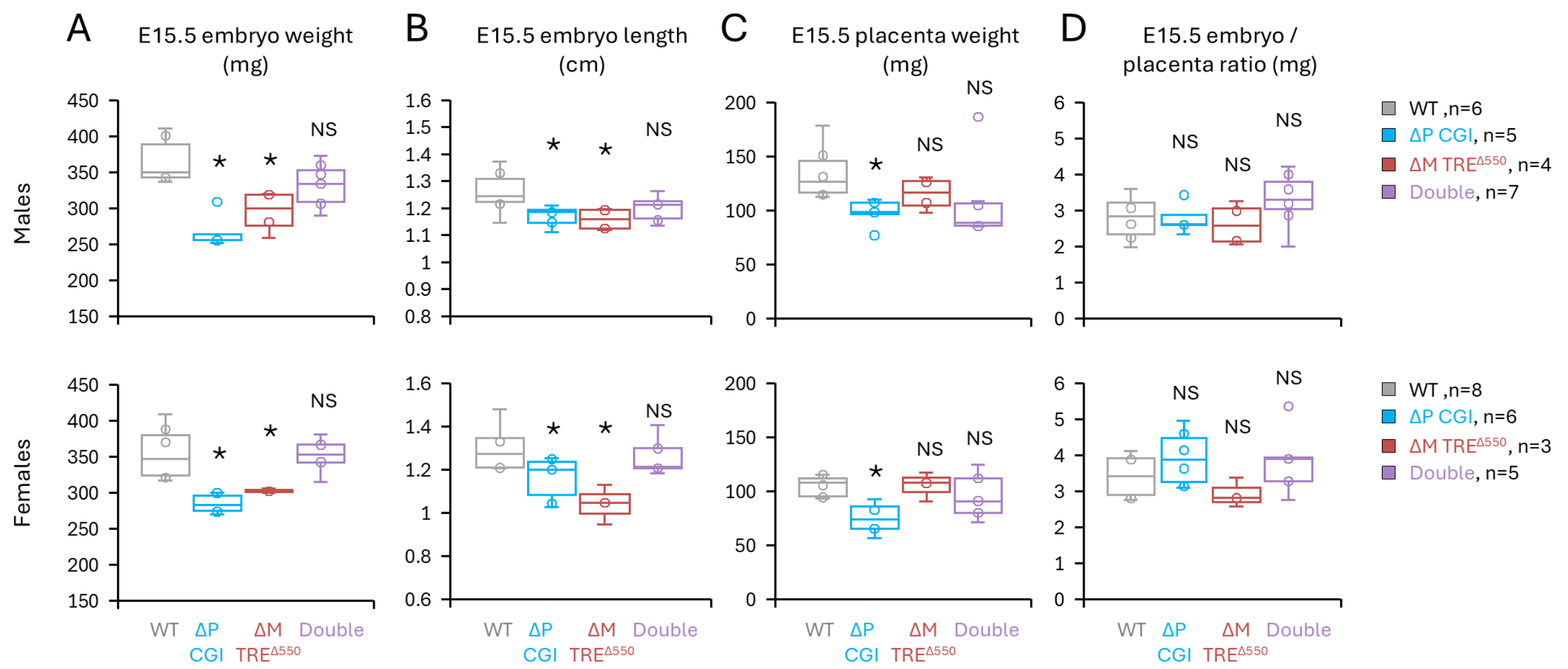

Figure S11- ΔP-IG-CGI and ΔM-IG-TRE$^{\Delta550}$ double deletion embryos are comparable in weight, length and placenta weight to WT littermates. A. Box plot representation of E15.5 embryo weight (mg) by sex. For WT, ΔP CGI, ΔM TRE$^{\Delta550}$ and double deletion males: Min= 336.5, 252.1, 259.1, 290.2; Q1= 342.9, 255.4, 275.5, 308.9; Center line= 349.5, 255.6, 299.8, 333.7; Q3= 389.1, 263.6, 318.9, 353.2; Max= 410.6, 308.9, 319.4, 372.9; n= 6, 5, 4, 7; $p_{\Delta P\text{-}CGI}$=0.0003, $p_{\Delta M\text{-}TRE}$=0.01. For WT, ΔP CGI, ΔM TRE$^{\Delta550}$ and double deletion females: Min= 317.2, 269.4, 300.2, 314.4; Q1= 323.8, 275.2, 301.1, 342; Center line= 347.2, 282.7, 302, 353.1; Q3= 379.6, 295.7, 304, 366.4; Max= 409.1, 299.3, 306, 380.4; n= 8, 6, 3, 5; $p_{\Delta P\text{-}CGI}$=0.001, $p_{\Delta M\text{-}TRE}$=0.04. **B.** Box plot representation of E15.5 embryo crown-rump length (cm) by sex. For WT, ΔP CGI, ΔM TRE$^{\Delta550}$ and double deletion males: Min= 1.15, 1.11, 1.12, 1.14; Q1= 1.22, 1.15, 1.12, 1.16; Center line= 1.24, 1.19, 1.16, 1.21; Q3= 1.31, 1.19, 1.19, 1.23; Max= 1.37, 1.21, 1.2, 1.26; n= 6, 5, 4, 7; $p_{\Delta P\text{-}CGI}$=0.05, $p_{\Delta M\text{-}TRE}$=0.05. For WT, ΔP CGI, ΔM TRE$^{\Delta550}$ and double deletion females: Min= 1.21, 1.03, 0.95, 1.18; Q1= 1.21, 1.08, 1, 1.21; Center line= 1.27, 1.2, 1.05, 1.21; Q3= 1.35, 1.24, 1.09, 1.3; Max= 1.48, 1.25, 1.13, 1.41; n= 8, 6, 3, 5; $p_{\Delta P\text{-}CGI}$=0.03, $p_{\Delta M\text{-}TRE}$=0.004. **C.** Box plot representation of E15.5 placenta weight (mg) by sex. For WT, ΔP CGI, ΔM TRE$^{\Delta550}$ and double deletion males: Min= 112.8, 77, 97.9, 85.4; Q1= 116.5, 96.6, 104.7, 86.1; Center line= 126.8, 98.3, 116.7, 88.3; Q3= 145.8, 107.3, 127.4, 106.7; Max= 178.7, 109.6, 130.6, 186.4; n= 6, 5, 4, 7; $p_{\Delta P\text{-}CGI}$=0.02. For WT, ΔP CGI, ΔM TRE$^{\Delta550}$ and double deletion females: Min= 93.3, 56.6, 90.5, 71; Q1= 95, 65.2, 99.1, 80; Center line= 107.6, 73.9, 107.6, 90.7; Q3= 111.9, 86, 112.3, 112; Max= 115.1, 92.2, 116.9, 124.2; n= 8, 6, 3, 5; $p_{\Delta P\text{-}CGI}$=0.0005. **D.** Box plot representation of E15.5 embryo/placenta weight ratio (mg) by sex. For WT, ΔP CGI, ΔM TRE$^{\Delta550}$ and double deletion males: Min= 2, 2.3 2.1, 2; Q1= 2.3, 2.6, 2.1, 3; Center line= 2.8, 2.6, 2.6, 3.3; Q3= 3.2, 2.9, 3.1, 3.8; Max= 3.6, 3.4, 3.3, 4.2; n= 6, 5, 4, 7. For WT, ΔP CGI, ΔM TRE$^{\Delta550}$ and double deletion females: Min= 2.8, 3.1, 2.6, 2.8; Q1= 2.9, 3.3, 2.7, 3.3; Center line= 3.4, 3.9, 2.8, 3.9; Q3= 3.9, 4.5, 3.1, 3.9; Max= 4.1, 5, 3.4, 5.4; n= 8, 6, 3, 5. NS- not significant. Asterisks indicate statistical significance in comparison to wildtype using a two-tailed unpaired Student's t-test. Source data are provided as a Source Data file.

Table S1- primers used to generate and sequence luciferase constructs

Fragment	Primer	Sequence 5' → 3'
IG-DMR 5'	SF8 (SacI) forward	CACGGCACAGAGCAGTAGCG
	SR9 (SacI) reverse	ACTGCCATTTAGAATTTGAG
IG-DMR Mid	IG-DMR Mid 1F NheI forward	AACTACCGCTACGGTTCATA
	IG-DMR Mid 1R NheI reverse	CATCCCACGAGGAACTGGT
	Mid forward	AACTACCGCTACGGTTCATA
	Mid NheI reverse	GGCCGCTAGCCATCCCACGAGGAACTGGT
	Mid KpnI reverse	GGCCGGTACCCATCCCACGAGGAACTGGT
IG-DMR 3'1	IGDMR 3'1F NheI forward	CCTTAGGGCTTCCAAGC
	IGDMR 3'1R NheI reverse	CTGGCGAGCAACAGTGAA
IG-DMR 3'2	IGDMR 3'2F NheI forward	GCTTCACTGTTGCTCGCC
	IGDMR 3'2R NheI reverse	CATGCTCAGAGCCAGCAG
315bp	NheI forward (also used for 550bp)	GGCCGCTAGCCGGTCTCGGAGTTCTTGTC
	NheI reverse	GGCCGCTAGCAGACGAGTTGGCAGGACG
	KpnI forward (also used for 550bp)	GGCCGGTACCCGGTCTCGGAGTTCTTGTC
	KpnI reverse	GGCCGGTACCAGACGAGTTGGCAGGACG
550bp	NheI reverse (also used for 235bp)	GGCCGCTAGCTGGTCAACAAAAGCATCTCTGT
	KpnI reverse (also used for 235bp)	GGCCGGTACCTGGTCAACAAAAGCATCTCTGT
235bp	NheI forward	GGCCGCTAGCGAGCCCTGTGGTTAACTAAGC
	KpnI forward	GGCCGGTACCGAGCCCTGTGGTTAACTAAGC
pGL3 empty vector	RV primer forward	CTAGCAAAATAGGCTGTCCC
	GL primer reverse	TATGTTTTTGGCGTCTTCCA
pGL3 promoter vector	Forward	AGGTACCGAGCTCTTACGC
	Reverse	GCCGGGCCTTTCTTTATGTT
pGL3 enhancer vector	Forward	GGGAGGTGTGGGAGGTTTT
	Reverse	AAGGAGCTGACTGGGTTGAA

Table S2- gRNAs and genotyping primers for CRISPR in mESCs

Target	Primer	Sequence 5' → 3'
5' IG-TRE	gRNA 1 forward	CACCGCTGTAGCCTTGCTAGACTG
	gRNA 1 reverse	AAACCAGTCTAGCAAGGCTACAGC
3' IG-TRE	gRNA 2 forward	CACCgCCATGGCTTACAAGCTACTG
	gRNA 2 reverse	AAACCAGTAGCTTGTAAGCCATGGC
SOX2-BS	gRNA forward	CACCgAGAAGGGATGAGACTCCTCT
	gRNA reverse	AAACAGAGGAGTCTCATCCCTTCTC
	single-stranded oligo donor (ssODN)	ACGGCTCTTTCCCAGAATCCACTGGGCCTGTTTTGGGGGCAGCT TAGTTAACCACAGGGCTCTGGTTAGAAGGGATGAtACgCCTCTAG GATTCCCAGAGTTTCTCGGTCTTCT
ZFP281-BS	gRNA forward	CACCgTCTCTAATCTTAGAGGCCTT
	gRNA reverse	AAACAAGGCCTCTAAGATTAGAGAC
	single-stranded oligo donor (ssODN)	GGCAACCTGGGGAGGAGGCATTGATATTATTCTTACTCTTGACCC TGTGAGAGATGCTCAGAAAGGCACACTGTTCAAAGAAAAAATTTC TGAGACgTAAGGCCTCTAAGATTAGAGATTGCTATATAGTC
PRDM14-BS	gRNA forward	CACCGCTGTAGCCTTGCTAGACTG
	gRNA reverse	AAACCAGTCTAGCAAGGCTACAGC
	single-stranded oligo donor (ssODN)	CCCTGTGAGAGATGCTCAGAAAGGCAGTGGGGGAAGACACTGTT CAAAGAAAAAATTTCTGAGACCTATTAGAGATTGCTATATAGTCgA CAGTCTAGCAAGGCTACAGCAGGGAGACAAGAA
IG-TRE Δ	Genotyping forward	TGACTTCCTTCAGCCACAGT
	Genotyping reverse	GGCAAACCCCAACTTAGCAA
	Genotyping internal reverse	CAGGATGCCAAGGGTTGTG
SOX2-BS Δ, ZFP281-BS Δ, PRDM14-BS Δ	Genotyping forward	CGGTCTCGGAGTTCTTGTC
	Genotyping reverse	AGACGAGTTGGCAGGACG

Table S3- primers for bisulfite sequencing

Target	Genomic coordinates of assay (mm10)	Target SQ (converted)	Primer	Sequence 5' → 3'
IG-CGI<br>With biotin for pyro methylation analysis	chr12:109528206-109528523 (7 CpGs)	TGTYGTTYGTTATGAATTATYGTTAYGGTTTATAGTGGATAGTTAGTGTYGTAGATYGTTATGGATTGGTGTTAAGGTTYGTTATGGA	Forward	GTGGTTTGTTATGGGTAAGTTT
			Reverse	CCCTTCCCTCACTCCAAAAATTAA
			Sequencing	TGGTTTATTGTATATAATGT
IG-TRE<br>With biotin for pyro methylation analysis	chr12:109530318-109530494 (4 CpGs)	RAAATTAAATTACRACAAACCRCCRACAACTAACAAACCTCAAATAATTA	Forward	GTTGGGGTTTGTTAGTTATTTATATGTTAT
			Reverse	AAAACATACTCTCCACTATAACTAATT
			Sequencing	CTATAACTAATTACAACACCAC
Gtl2-DMR<br>With biotin for pyro methylation analysis	chr12:109541110-109541226 (4 CpGs)	AGAYGATATTATTATTTGGTTTYGGTTTYGTTTTTTTTGGATATGTYGAAAGGTTAGTGTTGGGGATTTTTT	Forward	AGTTATTTTTTGTTTGAAAGGATGTGTA
			Reverse	CTAACTTTAAAAAAAAATCCCCAACACT
			Sequencing	GAAAGGATGTGTAAAAATGA

Table S4- primers for qPCR for gene expression

Target	Primer	Sequence 5' → 3'
GAPDH- housekeeping gene (ex2F+3R)	Forward	AGGTCGGTGTGAACGGATTTG
	Reverse	TGTAGACCATGTAGTTGAGGTCA
B2M- housekeeping gene (ex1F+2R)	Forward	GGCTGTATTCCCCTCCATCG
	Reverse	CCAGTTGGTAACAATGCCATGT
Dlk1- ex5<br>With biotin for qPCR and pyro SNP analysis	Forward	CGCAAGAAGAAGAACCTCCTGT
	Reverse	ACGCTGCTTAGATCTCCTCATCA
	Sequence	CAGCCTCCTTGTTGAA
Gtl2- ex10<br>With biotin for qPCR and pyro SNP analysis	Forward	AGCCACCTATTTACAAATGGACTC
	Reverse	CATCCCCATGAGAAACCTGTT
	Sequence	CATAGAGACACAAACATAGT
Rtl1- embryonic brain<br>(from: Ito *et al.* PMID: 26138477)	Forward	CGGGAAGCCTTTGATCTTG
	Reverse	TGTCGTCGGTTGGAAAGGAGTGTGC
Mirg- ex16<br>With biotin for qPCR and pyro SNP analysis	Forward	CTCAGGAGCGGATGTTCAAG
	Reverse	GATGTCCCCAGTGGAATGTC
	Sequence	GGAACCCTGCCTATG
Rian- ex4<br>With biotin for qPCR and pyro SNP analysis	Forward	GAGACCTTGGCAGTGACCG
	Reverse	CCTGGCCCAAAAAGCCTC
	Sequence	CTTGGCAGTGACCGC
Dio3- ex4<br>With biotin for qPCR and pyro SNP analysis	Forward	GAGGGATGCGAGAACTTTTTG
	Reverse	GCGCTTTCTGCCTAGGACT
	Sequence	TTTTGGAGAAGGGATT

Table S5- primers for ChIP-qPCR

Target	Primer	Sequence 5' → 3'
SOX2 Binding site at IG-TRE	Forward	TCCAATCTTCAAACACCTCCTG
	Reverse	TCCACTGGGCCTGTTTTGG
SOX2 Binding site at IG-TRE With biotin for qPCR and pyro SNP analysis	Forward	CCAATCTTCAAACACCTCCTG
	Reverse	CCACTGGGCCTGTTTTGG
	Sequence	CAAAACAATAAGAAGGGA
SOX2 Binding at H19-ICR- positive control (from: Zimmerman *et al*. PMID: 24324735)	Forward	AGCTGAGCCCCATTGAGAG
	Reverse	CCTGACCTCCCTACCCAAAG
SOX2 Binding at Ceacam1- negative control (from: Boumahdi *et al*. PMID: 24909994)	Forward	CTGAAGAGTGGATGGTAAGG
	Reverse	CACACCGCAAGGTCAGAATG
ZFP281 Binding site #1 at TRE With biotin for qPCR and pyro SNP analysis	Forward	AGTGAGGGAAGGGCTGCATTA
	Reverse	GCTAACCTGAGCTCCATGCTATTG
	Sequence	ATGCCTTGAGCACAG
ZFP281 Binding site #2 at TRE With biotin for qPCR and pyro SNP analysis	Forward	TGACTTCCTTCAGCCACAGTTC
	Reverse	GATGCTCAGAAAGGCAGTGG
	Sequence	GTGCATGCAGGATCGC
ZFP281 Binding site #3 downstream of TRE With biotin for qPCR and pyro SNP analysis	Forward	TGATTCACCACAATTCGTTCTG
	Reverse	CGGTCATCAATTTGCCATTTCT
	Sequence	CGTTCTGGTTTGTGATTA
ZFP281 Binding site #4 upstream of Gtl2 With biotin for qPCR and pyro SNP analysis	Forward	TTCGTGTTAGCGAGACAACATT
	Reverse	CCACCCCCAGGAGAAAGTATATC
	Sequence	TGTTAGCGAGACAACATT
ZFP281 Binding site #5 at Gtl2-DMR With biotin for qPCR and pyro SNP analysis	Forward	ATGGGGGTGCATAGCGAC
	Reverse	AGACTCCAATAGCCCAACCACC
	Sequence	GCCCAACCACCTGAG
ZFP281 Binding site #6 at Gtl2-DMR	Forward	TCATCTGTACCCCTCCCATTG
	Reverse	AGGGAAATGGGTAGGGGC
ZFP281 Binding at Otx2 promoter- positive control (from: Huang *et al*. PMID: 29168693)	Forward	CCCGAGGAAAGCAATTACAA
	Reverse	AAATGGCCCCAATCAAGTTT
ZFP281 Binding at Otx2- negative control (from: Huang *et al*. PMID: 29168693)	Forward	AATGCCTGGCTAAAACTGGA
	Reverse	CTTCAATGCTGACTGCTTGG

Table S6- gRNAs and genotyping primers for CRISPR in mice

Target	Primer	Sequence 5' → 3'
5' IG-TRE	sgRNA 1	CAGTCTAGCAAGGCTACAGC, PAM: AGG
	sgRNA 2	ATATAGTCCACAGTCTAGCA, PAM: AGG
3' IG-TRE	sgRNA 3	ATATAGTCCACAGTCTAGCA, PAM: TGG
	sgRNA 4	TTGGTTTACCAGTTCCTCGT, PAM: GGG
single-stranded oligo donor (ssODN)	CCTTCAGCCACAGTTCCCAGGCAGCCCTTGGCTGGAGGCATTGCGATCCT GCATGCACTTACAACACCGGTCTCGGAGTTCTTGTCTCCCTGCTGTAGCCG TGTCTGCTGCAGTGTGCCAGTTTTTTCGTGGTACACAGAGATGCTTTTGTTG ACCACAACCCTTGGCATCCTGGCTCACCGTTGTCTAGAAACACTTTT	
IG-TRE Δ550	Genotyping forward	GCCGCTATGCTATGCTGTTTC
	Genotyping reverse	GTGCCTCAGTGCCGTGTTAG
	Sequencing forward	CCCAGGACCTCCAATAGCAT
Off target 1: chr12:54356165-54356187 (Intergenic) ATACAGGCTACAGTCTAGCA TGG	Off target 1 forward	TACAGAGGGACTCTGGTATCTACA
	Off target 1 reverse	GGGATTTGTGGGTCTCGCA
	Off target 1 sequence forward	TCTACATGCAAAAGATGAACTGC
Off target 2: chr12:109960424-109960446 (Intergenic) AAAGAGTTCCCAGTCTAGCA GGG	Off target 2 forward	ATGACCTCAGTCTGCAAGGC
	Off target 2 reverse	TCCAGGGCAGGCATTACAAG
	Off target 2 sequence forward	ACCTGGAGTGGTGAGCCTAT
IG-TRE copy count taqman assay	Forward	CCCAGGTTGCCCTTAGTAAAT
	Reverse	CCAGAGTTTCTCGGTCTTCTTT
	Probe (FAM)	TGCGGGAGACACCCTGTTTCTTTA
Dot1l copy count reference taqman assay	Forward	GCCCCAGCACGACCATT
	Reverse	TAGTTGGCATCCTTATGCTTCATC
	Probe (VIC)	CCCAACAGGCCTGGATTCTCAATGC

Table S7- Number of surviving double deletion mice

# Litters	# Pups: live (dead)			
	WT	ΔP CGI	ΔM TRE^{550}	ΔM TRE^{550} & ΔP CGI
7	11	(4)	10 (1)	12 (4)
