# Supplementary material for: Regulatory mechanisms of maternal imprinting at the murine Dlk1-Dio3 domain
Source: Nat Commun. 2026 Sep 2;17:9353. doi: 10.1038/s41467-026-77129-4 (PMC13538571; doi:10.1038/s41467-026-77129-4)
Supplement: Supplementary file 2 [file 41467_2026_77129_MOESM2_ESM.pdf]

nature portfolio

Corresponding author(s): Prof. Anne Ferguson-Smith

Last updated by author(s): Aug 6, 2026

Reporting Summary

Nature Portfolio wishes to improve the reproducibility of the work that we publish. This form provides structure for consistency and transparency in reporting. For further information on Nature Portfolio policies, see our Editorial Policies and the Editorial Policy Checklist.

Statistics

For all statistical analyses, confirm that the following items are present in the figure legend, table legend, main text, or Methods section.

n/a	Confirmed	
☐	☒	The exact sample size (*n*) for each experimental group/condition, given as a discrete number and unit of measurement
☐	☒	A statement on whether measurements were taken from distinct samples or whether the same sample was measured repeatedly
☐	☒	The statistical test(s) used AND whether they are one- or two-sided<br>*Only common tests should be described solely by name; describe more complex techniques in the Methods section.*
☐	☒	A description of all covariates tested
☒	☐	A description of any assumptions or corrections, such as tests of normality and adjustment for multiple comparisons
☐	☒	A full description of the statistical parameters including central tendency (e.g. means) or other basic estimates (e.g. regression coefficient) AND variation (e.g. standard deviation) or associated estimates of uncertainty (e.g. confidence intervals)
☐	☒	For null hypothesis testing, the test statistic (e.g. *F*, *t*, *r*) with confidence intervals, effect sizes, degrees of freedom and *P* value noted<br>*Give P values as exact values whenever suitable.*
☒	☐	For Bayesian analysis, information on the choice of priors and Markov chain Monte Carlo settings
☒	☐	For hierarchical and complex designs, identification of the appropriate level for tests and full reporting of outcomes
☒	☐	Estimates of effect sizes (e.g. Cohen's *d*, Pearson's *r*), indicating how they were calculated

Our web collection on statistics for biologists contains articles on many of the points above.

Software and code

Policy information about availability of computer code

Data collection	No software was used for data collection
Data analysis	Calculations of embryo length were measured as Feret's diameter with ImageJ.

For manuscripts utilizing custom algorithms or software that are central to the research but not yet described in published literature, software must be made available to editors and reviewers. We strongly encourage code deposition in a community repository (e.g. GitHub). See the Nature Portfolio guidelines for submitting code & software for further information.

Data

Policy information about availability of data

All manuscripts must include a data availability statement. This statement should provide the following information, where applicable:

- Accession codes, unique identifiers, or web links for publicly available datasets
- A description of any restrictions on data availability
- For clinical datasets or third party data, please ensure that the statement adheres to our policy

All relevant data and details of resources can be found within the article and its supplementary information. Source Data are provided with this paper.

Research involving human participants, their data, or biological material

Policy information about studies with human participants or human data. See also policy information about sex, gender (identity/presentation), and sexual orientation and race, ethnicity and racism.

Reporting on sex and gender	not applicable
Reporting on race, ethnicity, or other socially relevant groupings	not applicable
Population characteristics	not applicable
Recruitment	not applicable
Ethics oversight	not applicable

Note that full information on the approval of the study protocol must also be provided in the manuscript.

Field-specific reporting

Please select the one below that is the best fit for your research. If you are not sure, read the appropriate sections before making your selection.

[x] Life sciences [ ] Behavioural & social sciences [ ] Ecological, evolutionary & environmental sciences

For a reference copy of the document with all sections, see nature.com/documents/nr-reporting-summary-flat.pdf

Life sciences study design

All studies must disclose on these points even when the disclosure is negative.

Sample size	No sample size calculation was performed. At least three biological replicates were used for each measurement from at least three different litters. Each litter has a mix of deletion and WT littermates. To minimize litter specific effects we analyzed embryos from the same genotype from several litters, and at least more than three since three biological independent replicates is the minimum needed for statistical significance. For most analysis three was a minimum as the more samples tested the stronger the significance is. We therefore sampled enough embryos to obtain statistically significant results, without wasting to many animals (according to the Reduction ethical use of animal models).
Data exclusions	No data were excluded from the analysis
Replication	Results were replicated by analysis of embryos and pups from multiple litters to minimize litter specific effects and maximize reproducibility of data. Results were assessed in reciprocal hybrid background to exclude strain specific effects. All attempts at replication were successful.
Randomization	Embryos/pups were genotyped by PCR and allocated into experimental groups according to their genotype
Blinding	All measurements of embryos/pups were divided into groups by genotypes. Blinding was not possible because we needed to know which embryos were from the same genotype to analyze together. When analyzing images or weights of embryos/tissues to determine phenotypic differences blind analysis was performed by a third party investigator who did not know the genotype of the samples.

Reporting for specific materials, systems and methods

We require information from authors about some types of materials, experimental systems and methods used in many studies. Here, indicate whether each material, system or method listed is relevant to your study. If you are not sure if a list item applies to your research, read the appropriate section before selecting a response.

Materials & experimental systems

n/a	Involved in the study	
[ ]	[x]	Antibodies
[ ]	[x]	Eukaryotic cell lines
[x]	[ ]	Palaeontology and archaeology
[ ]	[x]	Animals and other organisms
[x]	[ ]	Clinical data
[x]	[ ]	Dual use research of concern
[x]	[ ]	Plants

Methods

n/a	Involved in the study	
[x]	[ ]	ChIP-seq
[x]	[ ]	Flow cytometry
[x]	[ ]	MRI-based neuroimaging

Antibodies

Antibodies used	Goat polyclonal SOX2 antibody (R&D Systems#AF2018) and Rabbit polyclonal Zfp281/ZNF281 antibody (abcam#ab101318)
Validation	Antibodies were used as recommended on the manufacturer's website and according to publications with the same antibodies: 5µg Goat polyclonal SOX2 antibody (R&D Systems#AF2018) or 5µg Rabbit polyclonal Zfp281/ZNF281 antibody (abcam#ab101318) were used per 5x10^6mESCs or 100mg brain tissue.<br>SOX2 and positive and negative targets were used according to the methods described in Boumahdi et al. PMID: 24909994.<br>ZNF281 positive and negative controls were used according to the methods described in : Huang et al. PMID: 29168693.

Eukaryotic cell lines

Policy information about cell lines and Sex and Gender in Research

Cell line source(s)	Mouse embryonic stem cells (C57BL/6J x CAST/EiJ hybrid mESCs, male, in house derived).
Authentication	Karyotyping, PCR and Sanger Sequencing
Mycoplasma contamination	All cell lines were tested negative for mycroplasma contamination
Commonly misidentified lines<br>(See ICLAC register)	No commonly misidentified cell lines were used in the study

Animals and other research organisms

Policy information about studies involving animals; ARRIVE guidelines recommended for reporting animal research, and Sex and Gender in Research

Laboratory animals	CRISPR constructs were delivered into 1-cell stage embryos from a C57BL/6J genetic background. Electroporated embryos were re-implanted in CD1 pseudo-pregnant females. CAST/EiJ (RRID:IMSR_JAX:000928) and mutant mice were maintained under a 12h light-dark cycle at 22°C degrees (+/- 2°C) and 55% humidity (+/-10%). Mice were monitored for health and activity and were given ad libitum access to water and standard mouse chow. For pure and hybrid breeding experiments, mice were mated at 8-12 weeks of age. Male and female heterozygote F1 mice carrying the mutation were crossed with wildtype CAST/EiJ or wildtype C57BL/6J, and F2 animals harboring the mutated allele were analyzed at different stages.
Wild animals	No wild animals were used in the study
Reporting on sex	Pups were sexed at weaning. no sex bias was observed between genotypes. Male and female adult pups and tissues were weighed against wildtype littermates from the same sex. Sex was not considered for embryo analysis.
Field-collected samples	No field collected samples were used in the study
Ethics oversight	All animal experiments were performed in accordance with the Animals (Scientific Procedures) Act 1986 Amendment Regulations 2012 following ethical review by the University of Cambridge Animal Welfare and Ethical Review Body. Animal experiments were approved by the UK Home Office project license #PP8193772. All efforts were made to minimize animal discomfort.

Note that full information on the approval of the study protocol must also be provided in the manuscript.

Plants

Seed stocks	*Report on the source of all seed stocks or other plant material used. If applicable, state the seed stock centre and catalogue number. If plant specimens were collected from the field, describe the collection location, date and sampling procedures.*
Novel plant genotypes	*Describe the methods by which all novel plant genotypes were produced. This includes those generated by transgenic approaches, gene editing, chemical/radiation-based mutagenesis and hybridization. For transgenic lines, describe the transformation method, the number of independent lines analyzed and the generation upon which experiments were performed. For gene-edited lines, describe the editor used, the endogenous sequence targeted for editing, the targeting guide RNA sequence (if applicable) and how the editor was applied.*
Authentication	*Describe any authentication procedures for each seed stock used or novel genotype generated. Describe any experiments used to assess the effect of a mutation and, where applicable, how potential secondary effects (e.g. second site T-DNA insertions, mosiacism, off-target gene editing) were examined.*
