# Supplementary material for: Regulatory mechanisms of maternal imprinting at the murine Dlk1-Dio3 domain
Source: Nat Commun. 2026 Sep 2;17:9353. doi: 10.1038/s41467-026-77129-4 (PMC13538571; doi:10.1038/s41467-026-77129-4)
Supplement: Supplementary file 3 [file 41467_2026_77129_MOESM3_ESM.pdf]

nature portfolio

Peer Review File

Regulatory Mechanisms of Maternal Imprinting at the Murine Dlk1-Dio3 Domain

Corresponding Author: Professor Anne Ferguson-Smith

This file contains all reviewer reports in order by version, followed by all author rebuttals in order by version.

Version 0:

Reviewer comments:

Reviewer #1

(Remarks to the Author)
Overall summary:
This study aims to elucidate the mechanisms regulating maternal allele expression within the Dlk1-Dio3 imprinted domain, focusing particularly on the intergenic transcriptional regulatory element (IG-TRE) located within the IG-DMR. The IG-DMR is known to comprise two subdomains, IG-CGI and IG-TRE, and several knockout mouse models have revealed that DNA methylation at the IG-CGI is essential for paternal allele regulation, whereas the IG-TRE plays a key role in maternal allele regulation. Previous work examining the IG-TRE reported that deletion of a ~2.7 kb region resulted in embryonic lethality when the mutant allele was maternally inherited, accompanied by downregulation of maternally expressed genes and upregulation of paternally expressed genes. This suggested that the deleted 2.7 kb region contains elements critical for maternal allele–specific regulation.
In the present study, the authors identify part of this regulatory mechanism. Using enhancer screening in cultured cells, they define a 550 bp segment within the IG-TRE (named TRE-550bp) that exhibits both enhancer and silencer activities and contains putative SOX2 and ZFP281 binding sites. They also show that SOX2 binds to both alleles, whereas ZFP281 binds preferentially to the hypomethylated maternal allele in a SOX2-dependent manner.
The authors further generated mice carrying a deletion of the TRE-550bp region and performed gene expression and phenotypic analyses. Maternal inheritance of the TRE-550bp deletion (ΔM IG TRE 550) led to increased expression of Dlk1 from the maternal allele and resulted in growth retardation. Moreover, embryos carrying both the paternal IG CGI deletion (ΔP IG CGI) and maternal TRE 550bp deletion (ΔM IG TREΔ550) showed rescue of the embryonic lethality observed in the ΔP IG CGI single mutants.
Overall, this study provides important insights into the regulatory function of the IG TRE by identifying enhancer-like activity and its binding factors, and by clarifying how deletion of a specific subregion affects in vivo expression and phenotype. However, the most significant concern is that the study does not identify the most critical functional element within the IG TRE. Although the authors successfully narrow down a 550 bp region with regulatory activity, the data presented do not allow the reader to conclude which specific sequence(s) within IG TRE are essential for imprinting regulation or for the severe embryonic lethality observed in the previously reported larger IG TRE deletion (Hara et al., PLOS One, 2025). As a result, the mechanistic insight into how the IG TRE controls maternal allele expression remains incomplete, and the overall impact of the study is consequently limited.

Major Points:
1. Lines 490, 537 — Cause of lethality in IG-CGIΔP mice
The authors discuss the rescue of lethality observed in IG-CGIΔP mice by the IG-TREΔ550;IG-CGIΔP double deletion mainly in the context of restored Dlk1 expression. However, Dlk1 knockout mice exhibit fetal growth retardation but are not lethal. In contrast, knockout of Rtl1, another paternally expressed gene within the same imprinted domain, results in fetal lethality. Although Rtl1 expression levels were not measured in IG-CGIΔP embryos in a previous study (Weinberg-Shukron et al., Nat Commun 13, 2022), Saito et al. demonstrated that Rtl1 expression is almost completely abolished in ΔIG-CGI mice. Therefore, it is reasonable to assume that Rtl1 is also substantially downregulated in the IG-CGIΔP allele used in the present study. These observations suggest that reduced Rtl1 expression, rather than Dlk1, is the critical driver of lethality in IG-CGIΔP embryos.
For this reason, the manuscript should include data showing how Rtl1 expression changes in both IG-TREΔ550;IG-CGIΔP double mutants and IG-CGIΔP single mutants.

2. Line 337 — Requirement for ZFP281 functional evidence
The authors state that “together they regulate expression of the genes on the maternal chromosome,” referring to SOX2 and ZFP281. However, the proposed involvement of ZFP281 in gene regulation requires functional evidence. In particular, analysis of Zfp281 knockout or knockdown is necessary to support this conclusion.

3. Line 490 — Number of surviving double-deletion mice
The authors claim that IG-TREΔ550;IG-CGIΔP double-deletion mice survive; however, the manuscript does not provide the actual number of surviving and non-surviving animals. Since penetrance is a critical issue in assessing viability phenotypes, the numbers of live and dead offspring for each genotype should be explicitly reported.

4. Lines 404– — Inconsistency between Dlk1 overexpression and reduced body size
The authors show that ΔM IG-TREΔ550 fetuses exhibit significantly reduced crown–rump length at E15.5. At the same time, Dlk1 expression in the brain is increased approximately twofold. A previous study (da Rocha et al., PLoS Genet 2009) reported that BAC transgenic mice with double doses of Dlk1 consistently displayed overgrowth from E16 to birth. These findings appear contradictory if Dlk1 alone is considered. The authors should address this discrepancy and provide a mechanistic explanation.

Minor Points:
1. Line 221
“Enhancer vectors” should likely be “promoter vectors.”

2. Fig. 2B
The sense and α-sense panels are not separated in the same manner as the other figure panels.
3. Line 364
What is “Fig. 6I”? Please clarify or correct the figure reference.

4. Line 369
The authors state that maternal allele–derived Dlk1 expression is slightly increased in ΔM IG-TREΔ550 mice. Although a P-value is provided, the percentage contribution of the maternal allele in WT and ΔM IG-TREΔ550 should be explicitly shown.

5. Line 408
“Fig. 7C” should be “Fig. 6C.”

6. Fig. 7A
Bar graphs for E15.5 and 9w are distinguished by solid versus dotted, but this distinction is difficult to recognize. A clearer visual representation is recommended.

7. Line 767
In PX459, Cas9 is driven by the CAG promoter, and sgRNA expression is controlled by the U6 promoter.

8. Line 812
The bisulfite PCR was performed with 50 cycles, which appears to be excessive and may increase the risk of amplification bias or artifacts. This is particularly relevant for the Gtl2-DMR analysis in Fig. 3D, where the reported methylation differences are in the range of 20–30%, and over-cycling could potentially distort these values. The authors should justify the use of 50 cycles or consider repeating the assay under conditions that minimize over-amplification.

Reviewer #2

(Remarks to the Author)
In this manuscript Weinber-Shukron and colleagues further characterize the imprinting and expression behavior of the fascinating Dlk1-Dio3 domain. Using both ES cell and mouse models the investigators describe the behavior of a transcriptional regulatory element (designated IG-TRE) within the ICR. While this element exhibits unanticipated behavior in ES cells, the use of F1 hybrid mouse models elegantly shows a minimal 550 element (with SOX2 and ZFP281 binding) deletion results in upregulation of Dlk1. Moreover, combining deletion of the minimal 550 IG-TRE deletion with the lethal IG-CGI deletion rescues lethality by balancing (restoring) Dlk1 expression, demonstrating the key role of Dlk1 expression defects in lethality. Moreover, these results re-emphasize the importance of using mouse models to elucidate complex expression and imprinting behavior. The results are very interesting and well described but I have some suggestions to improve the manuscript and make the conclusion regarding the ES cells and imprinting experiments more robust.

Specific Comments
1.The Introduction is rather long and could be tightened. For example, similar information is presented twice on AFF3.

2.Figure 3D: “methylation analysis showed that loss of the maternal 1.3kb IG-TRE, by either maternal or biallelic deletions, resulted in increased methylation at the downstream Gtl2-DMR”. Is there an explanation for gain of methylation at Gtl2-DMR in ES cells? I thought that this region was methylated later in development.

3.The DNA methylation and SOX2 analysis in F1 hybrid ES cells requires more explanation. First the baseline for the IG-Tre Sox2-BS pyrosequencing assay looks to be over 70%, with statistical significance being an extra 15-20% methylation. What are the details of this assay (they are covered briefly in the methods)? How many CpG sites are assayed? Is there a polymorphism to determine which allele is less methylated? It would help in the understanding of SOX2 binding in these cells, which looks to be biallelic (it is considered a pioneer factor and suggested to be immune to DNA methylation but doesn't seem to be the case in mouse). What happens to SOX2 binding in the paternal Sox2 mutant? Is it diminished to the same extent as in Sox2 maternal mutants? Also, in these cells, ZFP281 binding should be assayed in paternal deletion cells (Figure 4D/E) as this would be a good control.

4.Figure 5B/C. Why are there different numbers of animals in the weight and length graphs of 9 week old animals? Additionally, the numbers are not high enough for robust stats (and you can't use n=2 for stats).

5.Figure 5F. It would be helpful for the DNA methylation assay interpretation if the assayed areas were indicated by a schematic. Also, because the paternal deletion was assayed phenotypically and for gene expression, DNA methylation should be performed on these mice too.

6.Figures 6B/8C. The placental length is not a typical measure as investigators routinely use weight to measure general health of the conceptus. In fact, fetal/placental weight ratios are the most informative. Given sexual dimorphism in the placental, this should also be done for males and female separately.

7.Line 450. "We conclude that in vivo, SOX2 binding at the IG-TRE is required for ZFP281 binding and maternal gene regulation in adult brains, but that this is either not required or not
established yet in embryonic brains." Because only the IG-TRE 550 deletion was investigated in mice, this conclusion cannot be stated as definitive without generation of a SOX2 binding site mutation in vivo.

8. Line 531. "Increased Dlk1 expression, however, as in the maternal IG-TREΔ550 model, has no apparent mutant phenotype.." I would substitute apparent with "obvious" as it is difficult to say something has no "apparent" phenotype.

Reviewer #3

(Remarks to the Author)
Weinberg-Shukron et al., use a combination of elegant in vitro and in vivo model systems to dissect mechanisms of imprinting control at the Dlk1-Dio3 domain. They first map the regulatory regions of the Dlk1-Dio3 domain and identify both enhancer and silencer elements and link them to transcription factor interactions in mouse embryonic stem cells. They then go on to show that in adult brain SOX2 and ZFP281 contribute to enhancer function on the maternal chromosome by utilizing different mouse models. Besides refining the regulatory map of the Dlk1-Dio3 domain and defining roles for SOX2 and ZFP281 in the regulation of the locus, the manuscript shows that loss of expression at this domain is more detrimental than gain in vivo.
Major comments
1) Throughout the manuscript the authors use bisulphite pyro sequencing to measure DNA methylation levels. While they refer back to previous papers regarding primers in the material and methods section, it would be highly appreciated if they could somewhere indicate how many CpGs within the DMR (and their location) are covered with their set-up.
2) Lines 417-423. In this section of the manuscript the authors refer to differences in Dlk1 expression between mESCs, embryonic and adult brain. However, in the manuscript, these data are not show in a way that the reader can fully appreciate this. Could the authors provide RT-qPCR data for this?
3) For several of their experiments the authors utilise brains from 9-week old animals. Since brain cell-type composition is highly heterogeneous, can they at least comment on which part of the brain was taken for their analyses and whether this is consistent for all experiments, genotypes and biological replicates? While cell type heterogeneity is less dramatic for the E15.5 samples, it is still a heterogeneous tissue compared to e.g., mESCs. Have the authors considered that cell type heterogeneity could influence some of their findings and their interpretation?
4) My main concerns are with Figure 7, which summarises experiments that address SOX2 and ZFP281 occupancy at the maternal IG-TRE in embryonic (E15.5) and adult (9-weeks old) brain.
They conclude that SOX2 binding is weak in the embryo (Figure 7A). However, they also don't really see SOX2 binding at their positive control locus (Scmh1). Neither in embryonic, nor in adult brain. It would be good to at least clarify if 1) SOX2 is expressed at all (western blot) in the embryonic brain and 2) include a positive control locus where SOX2 binding is observed in both the embryonic and adult brain. Without these additional experiments it is also difficult to interpret the SOX2 ChIP-qPCR result for IG-TRE in adult brain. Further, it appears from Figure 7B that SOX2 binding in adult brain is "primarily maternal" rather than "maternal specific" in the wildtype. Could the authors comment on this?
Regarding ZFP281 occupancy, it looks like there is slightly more ZFP281 binding at the paternal allele in the embryo in the ΔTRE550 strain at site 2 (Figure 7D). This does not seem to be the case in mESCs (Figure 4E). While I acknowledge that the differences are not statistically significant, could the authors comment into this?
Based on the provided data, the authors conclude that "in vivo, SOX2 binding at the IG-TRE is required for ZFP281 binding and maternal gene regulation in adult brains, but that this is either not required or not established yet in embryonic brains. Conceptually, this is very interesting as it highlights that very likely, we are still far from completely understanding gene regulation at imprinting clusters. However, I find this conclusion rather speculative and more suitable for the discussion. In particular, the issues with the data in Figure 7 mentioned above need to be addressed. Further, they mention "other

elements within the 235 bp of the IG-TRE"; have they looked whether there are motifs for other TFs, or would the hypothesis be that some more generic TFs take over in the embryonic brain? And what about the, already above mentioned, cell type heterogeneity?

Minor comments
5) Line 408: Should (Fig. 7C) be (Fig. 6C)?
6) The authors observed reduced liver weight in maternal IF-TREΔ550 animals. What could be the relevance of this?
7) In Figure 6C, is there a reason that Dio3 was omitted? Would be interesting to see how it compares to the adult brain data shown in Figure 5E.

Reviewer #4

(Remarks to the Author)
The manuscript entitled "Regulatory Mechanisms of Maternal Imprinting at the Dlk1-Dio3 Domain" by Weinberg-Shukron et al. investigated the mechanisms by which the unmethylated maternal chromosome at this imprinted locus is regulated. It is firmly established that imprinting and expression of the paternal allele of the Dlk1-Dio3 locus is largely regulated by an imprinting control region (ICR) known as the IG-DMR, a complex regulatory region which harbors two discrete regulatory elements, IG-CGI and IG-TRE. Curiously, studies to define the specific effects of these elements on imprinting, allelic expression, and phenotypes has led to paradoxical findings. Therefore, in the present study the authors performed a comprehensive set of in vitro reporter assays to map the cis-acting sequences within the IG-DMR that positively or negatively regulate transcription. Initially, the authors cloned an extensive series of overlapping fragments of the IG-DMR and in both the sense and antisense orientations into two different luciferase reporters (Basic and Promoter) to define the minimal region(s) with either enhancing or silencing activity. After defining the minimal element with a positive effect on heterologous transcription they deleted this region in mouse ES cells to evaluate its effect on epigenetic modifications at the maternal and paternal alleles. In addition, the authors identified candidate transcription factors (TFs) that bind to the IG-CGI and IG-TRE and their cooperativity on this element. These assays were complemented by generating knockout mice harboring a deletion in the IG-TRE and then examining the effects it had on methylation at these regulatory elements in mutant brains. Overall, this study presents a comprehensive in vitro and in vivo examination of the bipartite IG-DMR at the Dlk1-Dio3 locus. This is a critical imprinting regulatory element whose mutational analyses has revealed profound yet often confounding effects on imprinting and allelic expression in this domain. Thus, the fine-mapping of cis-acting sequences of the IG-DMR is clearly warranted. Indeed, the extent of IG-DMR fragments analyzed and the follow up CRISPR-induced deletions in ES cells and in mice is impressive. However, there are lingering concerns as described below.

Major Concerns:
1. In Fig. 3 and 4 the authors need to show the predicted DNA binding sites of SOX2 and ZFP281 to verify that they are bona fide DNA binding consensus sequences for these two TFs. Also, are these sites conserved in placental mammals (or at the very least in humans) that have the Dlk1-Dio3 locus? This could help reinforce the notion that both are important transcriptional regulators at the IG-DMR of this locus.
2. The ChIP experiments described in Fig 4. B-E with SOX2 and ZFP281 in ES cells are incomplete. The authors conclude that SOX2 binding occurs on the IG-TRE of both maternal and paternal alleles, and that its binding is diminished in ES cells harboring a deletion in the SOX2 binding site of the maternal allele. While this interpretation is appropriate for the maternal mutant allele, to definitively state that SOX2 binds to the paternal allele requires SOX2 ChIP analysis of this deletion in SOX2-BS delta P ES cells. Lastly, have both antibodies been examined for specificity and are ChIP-quality?
3. Relating to the experiments performed in in Fig. 3 and 4, the authors should inhibit SOX2 and ZFP281, individually and in combination, in mESC and evaluate the effects of these TFs on allelic imprinting at the IG-DMR including the effects on expression of Gtl2.
4. Fig. 5 (E,F) and Fig. 6 – no rationale is provided for the detailed molecular analyses in the brain of IG-TRE mutant mice. Why was this tissue examined for changes in gene expression of the noncoding RNAs and methylation status at the IG-DMR and Gtl2-DMR? Interestingly, there was no difference in brain weight, and presumably no phenotypic effect in mutant mice, yet this was the tissue examined for detailed imprinting effects. The authors reported differences in liver weight. Perhaps this tissue should have been given priority. While this locus is abundantly expressed in the brain, knockout mice with a deletion in one of the miRNA clusters in this Dlk1-Dio3 domain has a liver (metabolic) phenotype (Labialle et al 2014). It would make sense to perform these analyses in the liver.
Minor concern:
1. Lines 81-84, the authors describe regulation of noncoding RNAs at the Dlk1-Dio3 locus driven by the Gtl2 promoter. Although not the major thrust of the present study, the authors should consider citing Snyder et al 2013 and/or Pinheiro et al 2024 based on their characterization of the Gtl2 promoter in muscle cells and its effects on expression of these noncoding RNAs. These seem relevant to the introduction.

Version 1:

Reviewer comments:

Reviewer #1

(Remarks to the Author)

I thank the authors for their thorough and constructive responses. Most of my original points have been addressed satisfactorily; my remaining reservations are concentrated on the interpretation of the Rtl1 data, together with a few points of internal consistency.

Points adequately addressed
• Original Major 2 (ZFP281 functional evidence): rather than a Zfp281 knockout/knockdown, the authors provide deletions of the ZFP281 binding site (with a PRDM14 binding-site deletion as control) showing reduced maternal expression. This is a reasonable functional test of the element and satisfies the intent of my request.
• Original Major 3 (numbers of surviving animals): the per-genotype live/dead counts are now provided (Table S7), which addresses the penetrance concern.
• Original Major 4 (Dlk1 overexpression vs reduced body size): the expanded discussion (a non-imprinted, placenta-sparing BAC transgene versus a whole-domain deletion; a possible contribution of reduced Gtl2) is a reasonable explanation.
• The relevant minor points (figure-reference corrections, "promoter vector," and the added allelic-contribution panels) are addressed.

Remaining concerns
1. Rtl1 and the basis of the rescue. While the newly added Rtl1 data are a useful addition, the conclusion drawn from them is not yet fully supported.
First, the Rtl1 argument rests on brain measurements, whereas Rtl1's dosage-sensitive role is placental (the IG-CGIΔP placenta shows reduced weight and a thinner labyrinthine layer). I would therefore suggest tempering the discussion of this point rather than drawing a firm conclusion, unless the authors wish to support it with placental (and strand-specific) Rtl1 data.
Second, the response letter indicates that Rtl1 was measured with a strand-specific approach, but this is not reflected in the Methods (which describe oligo-dT/random-hexamer priming); presumably it was omitted, and a brief description should be added.

2. Consistency of the biallelic claim. The statement that (line 241)"Both maternal and biallelic deletions of the entire 1.3 kb resulted in loss of maternal expression of Gtl2, Rian and Mirg" is retained, but the corresponding pΔBi values and the associated supplementary reference appear to have been removed in this revision. I would suggest either restoring the biallelic statistics (with the number of independent clones) or limiting the statement to the maternal-deletion data.

3. Small residual items.
• The Methods still state that PX459 "expresses Cas9 under the control of a U6 promoter." In this vector the U6 promoter drives the sgRNA, whereas Cas9 is expressed from a CAG/CBh promoter. The response added the plasmid catalogue number but did not correct this statement, so the description remains inaccurate.
• Line 274: "l" appears to be a typographical error for "L."

Overall
The authors have responded conscientiously and the manuscript is improved, and the double-deletion rescue remains a notable result. My principal outstanding concern is that the interpretation of the Rtl1 data — and, relatedly, the molecular basis of the rescue — rests on brain measurements of a gene whose relevant function is placental; I would ask that this be appropriately qualified in the discussion, or supported with placental data.

Reviewer #2

(Remarks to the Author)
The authors did an excellent job addressing my comments and concerns. This paper is an important contribution to the field.

Reviewer #3

(Remarks to the Author)
In this revised manuscript, the authors have addressed all my previous concerns.

Reviewer #4

(Remarks to the Author)
The revised manuscript entitled "Regulatory Mechanisms of Maternal Imprinting at the Dlk1-Dio3 Domain" by Weinberg-Shukron et al. has been substantially improved from their initial submission. In this resubmission, the authors have provided extensive and informative additional experiments which has strengthened the notion that the TFs, SOX2 and ZFP281, are key regulators of the IG-DMR (IG-CGI and IG-TRE). Overall, this revised manuscript is quite strong and provides new and exciting data relating to in genomic imprinting at the Dlk1-Dio3 locus.

Open Access This Peer Review File is licensed under a Creative Commons Attribution 4.0 International License, which permits use, sharing, adaptation, distribution and reproduction in any medium or format, as long as you give appropriate credit to the original author(s) and the source, provide a link to the Creative Commons license, and indicate if changes were made.
In cases where reviewers are anonymous, credit should be given to 'Anonymous Referee' and the source.
The images or other third party material in this Peer Review File are included in the article's Creative Commons license, unless indicated otherwise in a credit line to the material. If material is not included in the article's Creative Commons license and your intended use is not permitted by statutory regulation or exceeds the permitted use, you will need to obtain permission directly from the copyright holder.
To view a copy of this license, visit https://creativecommons.org/licenses/by/4.0/

Point-by-point responses to reviewer comments

We thank the reviewers for their constructive feedback and suggestions for improvements to our manuscript. We have corrected all the minor comments and added substantial new data that we believe strengthen the manuscript and address all major concerns.

Reviewer 1:

Lines 490, 537- Cause of lethality in IG-CGIΔP mice: The authors discuss the rescue of lethality observed in IG-CGI$^{\Delta P}$ mice by the IG-TRE$^{\Delta 550}$;IG-CGI$^{\Delta P}$ double deletion mainly in the context of restored Dlk1 expression. However, Dlk1 knockout mice exhibit fetal growth retardation but are not lethal. In contrast, knockout of Rtl1, another paternally expressed gene within the same imprinted domain, results in fetal lethality. Although Rtl1 expression levels were not measured in IG-CGI$^{\Delta P}$ embryos in a previous study (Weinberg-Shukron et al., Nat Commun. 2022), Saito et al. demonstrated that Rtl1 expression is almost completely abolished in ΔIG-CGI mice. Therefore, it is reasonable to assume that Rtl1 is also substantially downregulated in the IG-CGI$^{\Delta P}$ allele used in the present study. These observations suggest that reduced Rtl1 expression, rather than Dlk1, is the critical driver of lethality in IG-CGI$^{\Delta P}$ embryos. For this reason, the manuscript should include data showing how Rtl1 expression changes in both IG-TRE$^{\Delta 550}$;IG-CGI$^{\Delta P}$ double mutants and IG-CGI$^{\Delta P}$ single mutants.

We thank the reviewer for this suggestion. First, Ito *et al*., have shown that loss of *Rtl1* has reduced viability but is not lethal (*Development*, 2015, PMID: 26138477). Similar to *Dlk1* knockout mice, the viable *Rtl1* knockout viable pups are growth retarded into adulthood. Regardless, *Rtl1* expression was not measured in our previous study because expression levels are very low in embryonic tissues, are largely restricted to the placenta, and are technically challenging to assess due to the presence of the antisense transcript. Nonetheless, we have now measured *Rtl1* expression using an embryonic brain-specific primer set and included this data in Figure 8H.

Both *Dlk1* and *Rtl1* expression are reduced in IG CGI$^{\Delta P}$ embryos. However, unlike *Dlk1*, *Rtl1* expression is not restored by the IG TRE$^{\Delta 550}$ deletion and remains markedly reduced in the rescued IG TRE$^{\Delta 550}$;IG CGI$^{\Delta P}$ double mutants. These findings indicate that reduced *Rtl1* expression alone cannot account for the lethality of IG CGI$^{\Delta P}$ embryos.

This interpretation is further supported by our placental data. As placental defects are thought to underlie the lethality of *Rtl1* mutant mice, we note that placental weights in the rescued double mutants were comparable to those of wild-type controls despite persistently low *Rtl1* expression. While we cannot exclude a contribution of *Rtl1* to the IG CGI$^{\Delta P}$ phenotype, the rescue of viability despite continued *Rtl1* downregulation indicates that additional factors, including restored *Dlk1* expression, contribute to the rescue. We have added the new *Rtl1* data and expanded the Discussion accordingly (lines 718-721).

Line 337- Requirement for ZFP281 functional evidence: The authors state that “together they regulate expression of the genes on the maternal chromosome,” referring to SOX2 and ZFP281. However, the proposed involvement of ZFP281 in gene regulation requires functional evidence. In particular, analysis of Zfp281 knockout or knockdown is necessary to support this conclusion.

Thank you for this suggestion. As requested, we have added experiments deleting the ZFP281 binding site from the 315bp of the IG-TRE and show that depletion of ZFP281 results in reduced maternal gene expression at this locus. These data are now included in the manuscript and in Figures 3, 4, S2, S3, S4 and S5.

Line 490- Number of surviving double-deletion mice: The authors claim that IG-TRE$^{\Delta 550}$;IG-CGI$^{\Delta P}$ double-deletion mice survive; however, the manuscript does not provide the actual number of surviving and non-surviving animals. Since penetrance is a critical issue in assessing viability phenotypes, the numbers of live and dead offspring for each genotype should be explicitly reported.

The numbers of live and dead offspring for each genotype in the double deletion cross are now reported in Table S7. They show that compared to 100% lethality of IG-CGI$^{\Delta P}$ pups, 75% of the double deletion pups survived postnatally.

Lines 404- Inconsistency between Dlk1 overexpression and reduced body size: The authors show that ΔM IG-TRE$^{\Delta 550}$ fetuses exhibit significantly reduced crown-rump length at E15.5. At the same time, Dlk1 expression in the brain is increased approximately twofold. A previous study (da Rocha et al., PLoS Genet 2009) reported that BAC transgenic mice with double doses of Dlk1 consistently displayed overgrowth from E16 to birth. These findings appear contradictory if Dlk1 alone is considered. The authors should address this discrepancy and provide a mechanistic explanation.

We thank the reviewer for this important comment. We do not consider these findings to be contradictory, as the two models differ substantially. The study by da Rocha *et al*. used a non-imprinted BAC transgene that resulted solely in *Dlk1* overexpression though not in the placenta, whereas the IG-TRE$^{\Delta 550}$ deletion causes developmental and tissue-specific changes in gene expression within the whole endogenous imprinted domain. Therefore, the resulting growth phenotype cannot be attributed to *Dlk1* dosage alone. Notably, reduced *Gtl2* expression is associated with growth restriction and may contribute to the phenotype.

Taken together, these observations suggest that the growth restriction observed in IG-TRE$^{\Delta 550}$ embryos reflects the combined consequences of altered regulation across the imprinted domain rather than the isolated effects of increased *Dlk1* expression. We have clarified this point in the Discussion of the revised manuscript, lines 733-740.

Minor points:
Line 221: “Enhancer vectors” should likely be “promoter vectors.”

Corrected.

Fig. 2B: The sense and α-sense panels are not separated in the same manner as the other figure panels.

Corrected as requested and made consistent with other figure panels.

Line 364: What is “Fig. 6I”? Please clarify or correct the figure reference.

Corrected.

Line 369: The authors state that maternal allele-derived Dlk1 expression is slightly increased in ΔM IG-TRE$^{\Delta 550}$ mice. Although a P-value is provided, the percentage contribution of the maternal allele in WT and ΔM IG-TRE$^{\Delta 550}$ should be explicitly shown.

Allelic expression percentages are now explicitly shown alongside relative expression graphs throughout the manuscript, including in Figure 5F.

Line 408: “Fig. 7C” should be “Fig. 6C.”

Corrected.

Fig. 7A: Bar graphs for E15.5 and 9w are distinguished by solid versus dotted, but this distinction is difficult to recognize. A clearer visual representation is recommended.

Figures 7A,C bar graph patterns were changed to better visualize the results.

Line 767: In PX459, Cas9 is driven by the CAG promoter, and sgRNA expression is controlled by the U6 promoter.

We used PX459 V2.0 (addgene plasmid #62988), which drives the gRNA expression under a U6 promoter. We added this catalogue number to our methods, line 866.

Line 812: The bisulfite PCR was performed with 50 cycles, which appears to be excessive and may increase the risk of amplification bias or artifacts. This is particularly relevant for the Gtl2-DMR analysis in Fig. 3D, where the reported methylation differences are in the range of 20-30%, and over-cycling could potentially distort these values. The authors should justify the use of 50 cycles or consider repeating the assay under conditions that minimize over-amplification.

Thank you for pointing this out. The bisulfite PCR was actually performed with 40 cycles, we corrected this typo in the methods, line 921.

<u>Reviewer 2:</u>

The Introduction is rather long and could be tightened. For example, similar information is presented twice on AFF3.

We have shortened the introduction by removing repetitive information.

Figure 3D: "methylation analysis showed that loss of the maternal 1.3kb IG-TRE, by either maternal or biallelic deletions, resulted in increased methylation at the downstream Gtl2-DMR". Is there an explanation for gain of methylation at Gtl2-DMR in ES cells? I thought that this region was methylated later in development.

We agree with the reviewer that *in vivo* methylation at the maternal Gtl2-DMR is normally established after implantation. However, secondary DMRs are sensitive to disruption of the regulatory mechanisms that maintain their epigenetic state, and aberrant methylation can arise in cultured ES cells when these mechanisms are compromised. We therefore interpret the increased methylation at the Gtl2-DMR following maternal deletion of the IG-TRE as evidence that this element contributes to protection of the maternal chromosome from DNA methylation, rather than a recapitulation of the normal developmental timing of Gtl2-DMR methylation acquisition. We have added text to the manuscript to clarify this point, lines 251-252.

The DNA methylation and SOX2 analysis in F1 hybrid ES cells requires more explanation. First the baseline for the IG-TRE Sox2-BS pyrosequencing assay looks to be over 70%, with statistical significance being an extra 15-20% methylation. What are the details of this assay (they are covered briefly in the methods)? How many CpG sites are assayed? Is there a polymorphism to determine which allele is less methylated? It would help in the understanding of SOX2 binding in these cells, which looks to be biallelic (it is considered a pioneer factor and suggested to be immune to DNA methylation but doesn't seem to be the case in mouse).

The target sequences and number of CpGs for each pyro-sequence assay are now included in Table S3, as well as illustrated alongside the location of the gRNAs in Figures 3B and S7E. Furthermore, methylation is now also plotted by CpG site in Figures S3, S4, S7 and S8.

Unfortunately, pyro-sequencing does not recognize polymorphisms for methylation analysis and therefore we could not distinguish between maternal and paternal alleles.

What happens to SOX2 binding in the paternal Sox2 mutant? Is it diminished to the same extent as in Sox2 maternal mutants? Also, in these cells, ZFP281 binding should be assayed in paternal deletion cells (Figure 4D/E) as this would be a good control.

As per the reviewer's suggestion, we have included SOX2 and ZFP281 binding in the paternal SOX2 binding site mutants, as well as SOX2 and ZFP281 binding in newly generated ZFP281 binding site mutants (Figure 4). These results show that SOX2 normally binds on the paternal chromosome in mESCs, and is depleted when the paternal SOX2-binding site is deleted. However, ZFP281 binding is unchanged by this loss of SOX2 binding on the paternal chromosome.

Figure 5B/C: Why are there different numbers of animals in the weight and length graphs of 9 week old animals? Additionally, the numbers are not high enough for robust stats (and you can't use n=2 for stats).

We have increased the number of animals weighed in Figures 5B,C and S6 and data are now shown for the same number of animals/tissues in all panels.

Figure 5F: It would be helpful for the DNA methylation assay interpretation if the assayed areas were indicated by a schematic. Also, because the paternal deletion was assayed phenotypically and for gene expression, DNA methylation should be performed on these mice too.

Methylation assay locations are now illustrated in Figures 3B and S7E and genomic coordinates are listed in Table S3. Furthermore, methylation and expression from brains and liver of animals with the paternal deletion of the IG-TRE$^{\Delta 550}$ is now shown in Figure S7.

Figures 6B/8C: The placental length is not a typical measure as investigators routinely use weight to measure general health of the conceptus. In fact, fetal/placental weight ratios are the most informative. Given sexual dimorphism in the placental, this should also be done for males and female separately.

We have collected the appropriate data and now show placenta weight and embryo/placenta weight ratios in Figures 6E,F and 8F,G. We have also plotted these for male and female embryos separately in Figures S8A-D and S11, but did not observe differences depending on sex, therefore both sexes are shown together in the main figures.

Line 450: "We conclude that in vivo, SOX2 binding at the IG-TRE is required for ZFP281 binding and maternal gene regulation in adult brains, but that this is either not required or not established yet in embryonic brains." Because only the IG-TRE 550 deletion was investigated in mice, this conclusion cannot be stated as definitive without generation of a SOX2 binding site mutation in vivo.

We rephrased the conclusion in lines 506-509 to avoid overclaiming the requirement for SOX2 binding *in vivo* using our IG-TRE$^{\Delta 550}$ deletion. We also added in our discussion that further experiments deleting the SOX2 binding site will aid in testing this model (lines 668-669).

Line 531: "Increased Dlk1 expression, however, as in the maternal IG-TREΔ550 model, has no apparent mutant phenotype" I would substitute apparent with "obvious" as it is difficult to say something has no "apparent" phenotype

As suggested, "apparent" was substituted with "obvious" in line 598.

Reviewer 3:

Throughout the manuscript the authors use bisulphite pyro sequencing to measure DNA methylation levels. While they refer back to previous papers regarding primers in the material and methods section, it would be highly appreciated if they could somewhere indicate how many CpGs within the DMR (and their location) are covered with their set-up.

The genomic coordinates, target sequences and number of CpGs for each pyro-sequence assay is now included in Table S3, as well as illustrated in Figures 3B and S7E. Furthermore, methylation is now also plotted by CpG site in Figures S3, S4, S7 and S8.

Lines 417-423: In this section of the manuscript the authors refer to differences in Dlk1 expression between mESCs, embryonic and adult brain. However, in the manuscript, these data are not show in a way that the reader can fully appreciate this. Could the authors provide RT-qPCR data for this?

Data is now shown for *Dlk1* expression levels between mESCs, embryonic and adult brain in Figure S9, and mentioned in lines 472-476.

For several of their experiments the authors utilise brains from 9-week old animals. Since brain cell-type composition is highly heterogeneous, can they at least comment on which part of the brain was taken for their analyses and whether this is consistent for all experiments, genotypes and biological replicates? While cell type heterogeneity is less dramatic for the E15.5 samples, it is still a heterogeneous tissue compared to e.g., mESCs. Have the authors considered that cell type heterogeneity could influence some of their findings and their interpretation?

To minimize cell type heterogeneity, for both embryonic and adult brains the entire brain was homogenised and taken for analysis rather than a specific brain subregion. We agree with the reviewer that imprinting in specific cell types or areas in the brain can be regulated differently, but by using bulk brain tissue we focus on the majority rather than the outlier populations. We clarified this in our method section (lines 1040-1041) and have added a cautionary sentence to the Discussion (lines 694-699) to emphasize that our observations apply to the measured tissue and should not be extrapolated to all cell types within the tissue, which may exhibit distinct regulatory responses.

My main concerns are with Figure 7, which summarises experiments that address SOX2 and ZFP281 occupancy at the maternal IG-TRE in embryonic (E15.5) and adult (9-weeks old) brain. They conclude that SOX2 binding is weak in the embryo (Figure 7A). However, they also don't really see SOX2 binding at their positive control locus (Scmh1). Neither in embryonic, nor in adult brain. It would be good to at least clarify if 1) SOX2 is expressed at all (western blot) in the embryonic brain and 2) include a positive control locus where SOX2 binding is observed in both the embryonic and adult brain. Without these additional experiments it is also difficult to interpret the SOX2 ChIP-qPCR result for IG-TRE in adult brain.

Thank you for this comment. We changed the positive control for the SOX2 ChIP-qPCR in both Figures 4B and 7A to the H19-ICR, which shows much stronger binding of SOX2 in the brain. We used a well-characterised goat anti-SOX2 antibody (R&D Systems, AF2018), which is ChIP-quality, has been validated by the manufacturer using knockout/knockdown approaches and is widely used to label neural progenitors in the mouse brain (e.g. Ferron *et al*. *Nature*, 2011 (PMID: 21776083) and Domingo-Muelas *et al*. *Cell Mol Life Sci*. 2023 (PMID: 36627412)).

Further, it appears from Figure 7B that SOX2 binding in adult brain is "primarily maternal" rather than "maternal specific" in the wildtype. Could the authors comment on this?

We have rephrased this section, and now describe the binding as "primarily maternal" instead of "maternal specific" (lines 502 and 524).

Regarding ZFP281 occupancy, it looks like there is slightly more ZFP281 binding at the paternal allele in the embryo in the ΔTRE550 strain at site 2 (Figure 7D). This does not seem to be the case in mESCs (Figure 4E). While I acknowledge that the differences are not statistically significant, could the authors comment into this?

A direct comparison cannot be meaningfully made between ZFP281 allelic binding percentages in mutant mESCs and in IG-TRE$^{\Delta550}$ embryonic brain tissue in Figures 4E and 7D, since the total levels of binding and allelic contribution in WT are not the same (very low in embryonic brain and 80:20 split compared to 70:30 in mESCs).

Based on the provided data, the authors conclude that "in vivo, SOX2 binding at the IG-TRE is required for ZFP281 binding and maternal gene regulation in adult brains, but that this is either not required or not established yet in embryonic brains". Conceptually, this is very interesting as it highlights that very likely, we are still far from completely understanding gene regulation at imprinting clusters. However, I find this conclusion rather speculative and more suitable for the discussion.

We rephrased the conclusion in this section, lines 507-510, to avoid overclaiming the requirement for SOX2 binding *in vivo* using our deletion of the IG-TRE$^{\Delta550}$. We also address this in the Discussion section, lines 668-669.

In particular, the issues with the data in Figure 7 mentioned above need to be addressed. Further, they mention "other elements within the 235 bp of the IG-TRE"; have they looked whether there are motifs for other TFs, or would the hypothesis be that some more generic TFs take over in the embryonic brain? And what about the, already above mentioned, cell type heterogeneity?

As suggested by the reviewer, we included an illustration of several other elements within the 315bp of the IG-TRE in Figure S4A,B, including the top scoring transcription factors on JASPAR, and their motifs and conservation. We also include data on deletion of the PRDM14 binding site from within the 315bp of the IG-TRE as a negative control that doesn't change expression or methylation at the region (Figure S4C-L). We have responded re cell-type heterogeneity in the above answer.

Minor comments:
Line 408: Should (Fig. 7C) be (Fig. 6C)?

Corrected.

The authors observed reduced liver weight in maternal IF-TREΔ550 animals. What could be the relevance of this?

The reduced liver weight observed in maternal IG-TRE$^{\Delta550}$ animals is currently descriptive and cannot be assigned a specific mechanistic interpretation based on the available data. This phenotype may reflect broader physiological consequences of altered imprinting regulation at the Dlk1-Dio3 locus, potentially affecting systemic growth or metabolic homeostasis, but further work would be required to determine whether it reflects a direct tissue-specific effect or an indirect consequence of developmental or endocrine changes. We have clarified in the

manuscript that this observation is correlative and does not support a direct functional conclusion at this stage (Discussion, lines 701-705).

In Figure 6C, is there a reason that Dio3 was omitted? Would be interesting to see how it compares to the adult brain data shown in Figure 5E.

Dio3 is now included in Figure 6G-J.

Reviewer 4:

In Fig. 3 and 4 the authors need to show the predicted DNA binding sites of SOX2 and ZFP281 to verify that they are bona fide DNA binding consensus sequences for these two TFs. Also, are these sites conserved in placental mammals (or at the very least in humans) that have the Dlk1-Dio3 locus? This could help reinforce the notion that both are important transcriptional regulators at the IG-DMR of this locus.

Thank you for this suggestion. We have included the motifs and conservation of the SOX2 and ZFP281 binding sites at the IG-TRE in Figure 3C, and show they are both highly conserved in placental mammals. In marsupial mammals, there is no imprinting at this domain and the entire IG-DMR is absent, including the IG-TRE and the SOX2 and ZFP281 transcription factor binding sites. We have also added in comparison the motifs and conservation of several other predicted transcription factors within the 315bp of the IG-TRE in Figure S4A,B, that are not well conserved. Reinforcing the importance of SOX2 and ZFP281 as transcriptional regulators at this locus.

The ChIP experiments described in Fig 4. B-E with SOX2 and ZFP281 in ES cells are incomplete. The authors conclude that SOX2 binding occurs on the IG-TRE of both maternal and paternal alleles, and that its binding is diminished in ES cells harboring a deletion in the SOX2 binding site of the maternal allele. While this interpretation is appropriate for the maternal mutant allele, to definitively state that SOX2 binds to the paternal allele requires SOX2 ChIP analysis of this deletion in SOX2-BS delta P ES cells.

We have now included, as per the reviewer's suggestion, SOX2 and ZFP281 binding in the paternal SOX2 binding site mutants, as well as SOX2 and ZFP281 binding in newly generated ZFP281 binding site mutants (Figure 4). These results show that SOX2 does normally bind on the paternal and maternal chromosome in mESCs and is depleted when the paternal SOX2-binding site is also deleted. However, ZFP281 binding is unchanged by this loss of SOX2 binding on the paternal chromosome.

Lastly, have both antibodies been examined for specificity and are ChIP-quality?

We used a well-characterised goat anti-SOX2 antibody (R&D Systems, AF2018), which is ChIP-quality, has been validated by the manufacturer using knockout/knockdown approaches and is widely used to label neural progenitors in the mouse brain (e.g. Ferron *et al*. *Nature*, 2011 (PMID: 21776083) and Domingo-Muelas *et al*. *Cell Mol Life Sci*. 2023 (PMID: 36627412)).

Relating to the experiments performed in in Fig. 3 and 4, the authors should inhibit SOX2 and ZFP281, individually and in combination, in mESC and evaluate the effects of these TFs on allelic imprinting at the IG-DMR including the effects on expression of Gtl2.

Thank you for suggesting this experiment. We have now deleted the ZFP281 binding site from within the 315bp of the IG-TRE in mESCs and evaluated the effect on imprinting at the region. Deleting the ZFP281 binding site does not influence SOX2 binding, but SOX2 binding is

required for ZFP281. This new data is included in Figures 3J-L, S2I-L, S3G-I, Figure 4 and S5. Since we find that deleting the SOX2 or ZFP281 binding sites individually has similar effects on transcription we do not believe there will be an additive effect on expression by deleting them in combination. Furthermore, our ChIP experiments show the effects of SOX2 and ZFP281 binding in reciprocal mutant binding sites, and these provide evidence of the relationship between the two transcription factors.

Fig. 5 (E,F) and Fig. 6 - no rationale is provided for the detailed molecular analyses in the brain of IG-TRE mutant mice. Why was this tissue examined for changes in gene expression of the noncoding RNAs and methylation status at the IG-DMR and Gtl2-DMR? Interestingly, there was no difference in brain weight, and presumably no phenotypic effect in mutant mice, yet this was the tissue examined for detailed imprinting effects. The authors reported differences in liver weight. Perhaps this tissue should have been given priority. While this locus is abundantly expressed in the brain, knockout mice with a deletion in one of the miRNA clusters in this Dlk1-Dio3 domain has a liver (metabolic) phenotype (Labialle et al 2014). It would make sense to perform these analyses in the liver.

Our lab has been interested in the genes at this locus and their function in the brain for many years, and our studies show that the brain represents a particularly informative tissue for studying regulation at this locus, as several genes within the domain, especially the maternal long non-coding RNAs, are highly expressed in this tissue, and *Dlk1* is known to be important for neurogenesis.

We agree with the reviewer that regulation of imprinting at this locus could be tissue specific and should be considered in a temporal and spatial manner, and therefore we also analyzed other tissues, such as the liver. Interestingly, as opposed to the brain, in livers from adult animals no change in gene expression was observed by deletion of either maternal or paternal IG-TRE$^{\Delta 550}$, and we therefore focused on the brain in the main figures. The results for both adult and embryonic liver are now shown in Figures S7C,D and S8E-H.

Minor comment:
Lines 81-84: the authors describe regulation of noncoding RNAs at the Dlk1-Dio3 locus driven by the Gtl2 promoter. Although not the major thrust of the present study, the authors should consider citing Snyder et al 2013 and/or Pinheiro et al 2024 based on their characterization of the Gtl2 promoter in muscle cells and its effects on expression of these noncoding RNAs. These seem relevant to the introduction.

The papers by Snyder *et al.* and Pinheiro *et al*. are now cited in the introduction, line 87, references#18,19.

Point-by-point responses to reviewer comments

We thank the reviewers for their time and suggestions that improved our manuscript. We have corrected the additional minor comments by reviewer #1, see below:

Reviewer #1:

I thank the authors for their thorough and constructive responses. Most of my original points have been addressed satisfactorily; my remaining reservations are concentrated on the interpretation of the Rtl1 data, together with a few points of internal consistency. Points adequately addressed:
• Original Major 2 (ZFP281 functional evidence): rather than a Zfp281 knockout/knockdown, the authors provide deletions of the ZFP281 binding site (with a PRDM14 binding-site deletion as control) showing reduced maternal expression. This is a reasonable functional test of the element and satisfies the intent of my request.
• Original Major 3 (numbers of surviving animals): the per-genotype live/dead counts are now provided (Table S7), which addresses the penetrance concern.
• Original Major 4 (Dlk1 overexpression vs reduced body size): the expanded discussion (a non-imprinted, placenta-sparing BAC transgene versus a whole-domain deletion; a possible contribution of reduced Gtl2) is a reasonable explanation.
• The relevant minor points (figure-reference corrections, "promoter vector," and the added allelic-contribution panels) are addressed.

Remaining concerns:
1. Rtl1 and the basis of the rescue. While the newly added Rtl1 data are a useful addition, the conclusion drawn from them is not yet fully supported. First, the Rtl1 argument rests on brain measurements, whereas Rtl1's dosage-sensitive role is placental (the IG-CGI∆P placenta shows reduced weight and a thinner labyrinthine layer). I would therefore suggest tempering the discussion of this point rather than drawing a firm conclusion, unless the authors wish to support it with placental (and strand-specific) Rtl1 data. Second, the response letter indicates that Rtl1 was measured with a strand-specific approach, but this is not reflected in the Methods (which describe oligo-dT/random-hexamer priming); presumably it was omitted, and a brief description should be added.

We thank the reviewer for this suggestion, and have clarified in the discussion that since these measurements were made in brain (albeit with primers for an *Rtl1* brain-specific isoform), it does not necessarily reflect the expression of *Rtl1* everywhere else in the embryo, such as in the placenta, and interpreting the results should take this into consideration. This is now included in the discussion, lines 578-583.
Regarding the method for *Rtl1* measurement, qPCRs were performed using an embryonic transcript-specific forward primer (Ex1c) and a common reverse primer in exon 3, which were previously designed and reported by Ito *et al*. (PMID: 26138477). cDNA was synthesized with oligo dT and random hexamer primers, same as in this paper. This reference is now mentioned in the list of qPCR primers in Table S4.

2. Consistency of the biallelic claim. The statement that (line 241)"Both maternal and biallelic deletions of the entire 1.3 kb resulted in loss of maternal expression of Gtl2, Rian and Mirg" is retained, but the corresponding p∆Bi values and the associated supplementary reference appear to have been removed in this revision. I would suggest either restoring the biallelic

statistics (with the number of independent clones) or limiting the statement to the maternal-deletion data.

We have corrected the phrasing and limited the statement to the maternal deletion data.

3. Small residual items.

• The Methods still state that PX459 "expresses Cas9 under the control of a U6 promoter." In this vector the U6 promoter drives the sgRNA, whereas Cas9 is expressed from a CAG/CBh promoter. The response added the plasmid catalogue number but did not correct this statement, so the description remains inaccurate.

We corrected this in the methods, lines 710-711.

• Line 274: "I" appears to be a typographical error for "L."

Corrected.

Overall The authors have responded conscientiously and the manuscript is improved, and the double-deletion rescue remains a notable result. My principal outstanding concern is that the interpretation of the Rtl1 data — and, relatedly, the molecular basis of the rescue — rests on brain measurements of a gene whose relevant function is placental; I would ask that this be appropriately qualified in the discussion, or supported with placental data.

We appreciate the reviewer's concern on *Rtl1*s function being mostly placental and have expressed this reservation in our discussion of our results, as elucidated in our response above.

<u>Reviewer #2:</u>

The authors did an excellent job addressing my comments and concerns. This paper is an important contribution to the field.

<u>Reviewer #3:</u>

In this revised manuscript, the authors have addressed all my previous concerns.

<u>Reviewer #4:</u>

The revised manuscript entitled "Regulatory Mechanisms of Maternal Imprinting at the Dlk1-Dio3 Domain" by Weinberg-Shukron et al. has been substantially improved from their initial submission. In this resubmission, the authors have provided extensive and informative additional experiments which has strengthened the notion that the TFs, SOX2 and ZFP281, are key regulators of the IG-DMR (IG-CGI and IG-TRE). Overall, this revised manuscript is quite strong and provides new and exciting data relating to in genomic imprinting at the Dlk1-Dio3 locus.
